# Synthesis of an A/B-*cis*-Fused Cyclopenta[*b*]fluorene (6/5/6/5) Ring System for Embellicine A via the Eight-Membered Silylene-Tethered IMDA Reaction

**DOI:** 10.1021/acs.orglett.5c04318

**Published:** 2026-01-27

**Authors:** Yuya Sakai, Ryoma Murata, Akihiro Hirakawa, Yusuke Nakatani, Tsubasa Maeda, Yuki Kuwano, Shunya Morita, Hiromi Uchiro

**Affiliations:** Faculty of Pharmaceutical Sciences, 26413 Tokyo University of Science, 6-3-1 Niijuku, Katsushika-ku, Tokyo 125-8585, Japan

## Abstract

The first synthesis of an A/B-*cis*-fused cyclopenta­[*b*]­fluorene (6/5/6/5) ring system was successfully achieved via an intramolecular Diels–Alder (IMDA) reaction of an eight-membered silylene-tethered precursor. In this key step, a significant conformational change in the diene moiety was achieved by introducing an eight-membered silylene tether, and the desired A/B-*cis*-fused tetracyclic compound was obtained with complete stereoselectivity. After several functional group manipulations on the D-ring, a fully elaborate tetracyclic fragment of embellicines was successfully synthesized.

## Graphical abstract

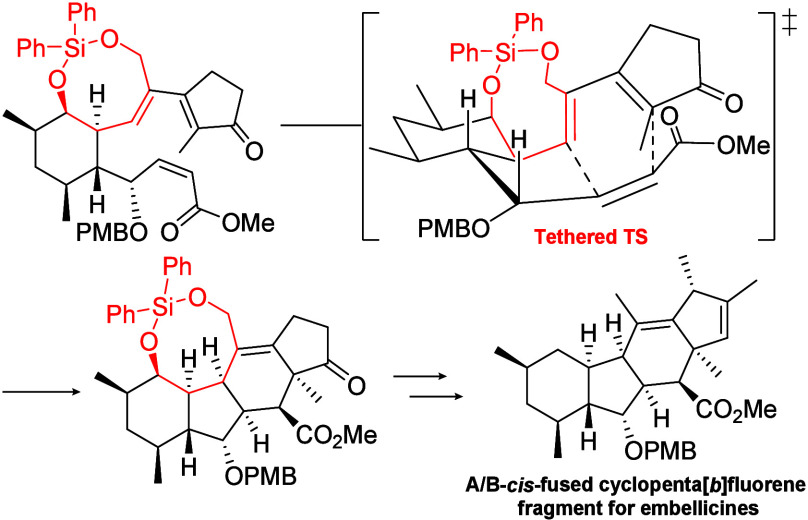

Recently, natural organic compounds containing cyclopenta­[*b*]­fluorene (6/5/6/5) ring systems have been reported. These are classified as A/B-*trans*-fused compounds such as phomapyrrolidones,^1^ ascomylactams,^2^ other related compounds,^3^ and A/B-*cis*-fused embellicines^4^ (Figure 1). Among these compounds, embellicines exhibit potent cytotoxic activity against human cancer cell lines. In particular, embellicines A (**1**) and B (**2**) strongly inhibit the TNFα-induced transcriptional activity of NF-κB and are expected to serve as new leads for anticancer and anti-inflammatory drugs.^4^ Therefore, we undertook the total synthesis of embellicines to elucidate their detailed structure–activity relationships. Herein, we report the first synthesis of an A/B-*cis*-fused cyclopenta­[*b*]­fluorene (6/5/6/5) fragment of embellicines.

**Figure 1:**
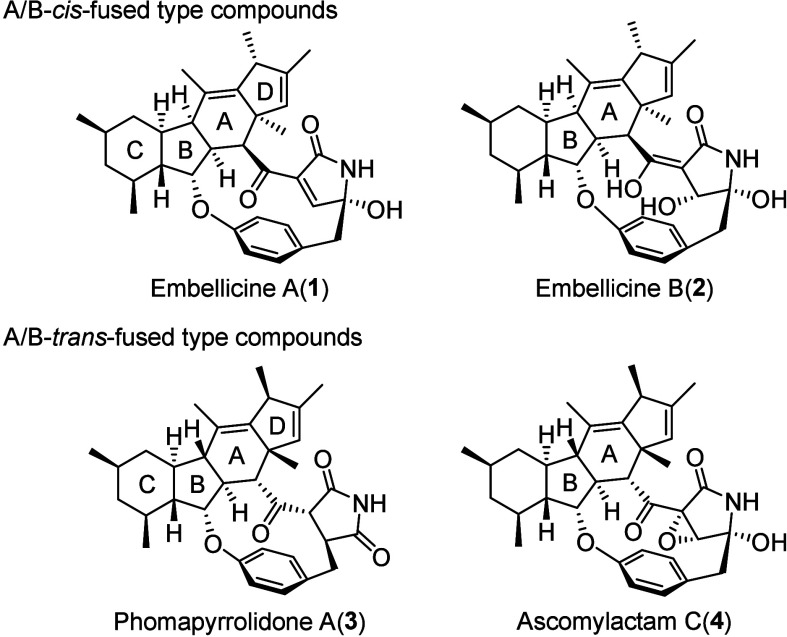
Structures of embellicine A (**1**) and related compounds.

The most challenging task in the synthesis of tetracyclic fragments of embellicines is constructing an A/B-*cis*-fused ring system. In 2012, Kobayashi et al. reported the synthesis of an A/B-*cis-*fused tricyclic decahydrofluorene skeleton of pyrrocidine A^5^ (Figure 2). They utilized a Diels–Alder reaction between five-membered cyclic enone containing the B/C-ring and a Danishefsky–Kitahara type diene to construct the A/B-*cis*-fused tricyclic framework. Although this synthetic strategy is reliable for constructing A/B-*cis*-fused structures, many additional reactions are required for functional group transformations on the A- and C-rings.

**Figure 2:**
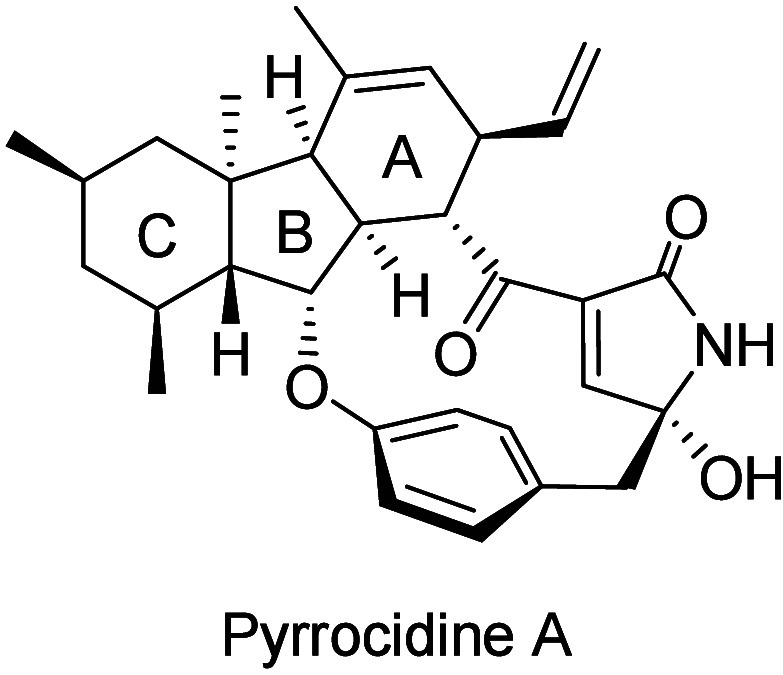
A/B-*cis*-fused type decahydrofluorene compound.

By contrast, we previously reported the efficient synthesis of A/B-*trans*-fused decahydrofluorene skeletons in the total synthesis of hirsutellone B^6^ and GKK1032A_2_.^7^ In this strategy, the intramolecular Diels–Alder (IMDA) reaction was effectively utilized for the stereoselective construction of the A/B-ring system. To apply a strategy similar to that used for the construction of the tetracyclic skeleton of embellicine A, it is necessary to completely reverse the stereoselectivity of the IMDA reaction from A/B-*trans*-selective to A/B-*cis*-selective.

Then we considered the transition state of the IMDA reaction (Scheme 1). In the thermodynamically stable transition state of the IMDA reaction (**TS-1**), the diene moiety would be distributed to the α-side toward the C-ring because, in the alternative transition state where the diene moiety is located on the β-side (**TS-2**), non-negligible steric repulsion between a methyl group of the diene moiety and the axial hydrogen on the C-ring should be generated. Therefore, we introduced a tethered structure into the IMDA precursor to reverse the orientation of the diene moiety toward the β-side of the C-ring. Specifically, two hydroxyl groups were introduced on the β-side of the C-ring and diene moiety. If these groups were connected by a silicon atom, the resulting eight-membered tether would play the desired role in the transition state of the IMDA reaction (**Tethered TS**). Furthermore, introducing a cyclopentanone structure at the terminus of the diene moiety would enable the simultaneous construction of the desired tetracyclic skeleton through the IMDA reaction. Although such Diels–Alder reactions utilizing electron-deficient dienes conjugated with carbonyl groups have rarely been reported,^8^ we dare to employ this type of reaction precursor considering the following modifications of the D-ring.

**Scheme 1:**
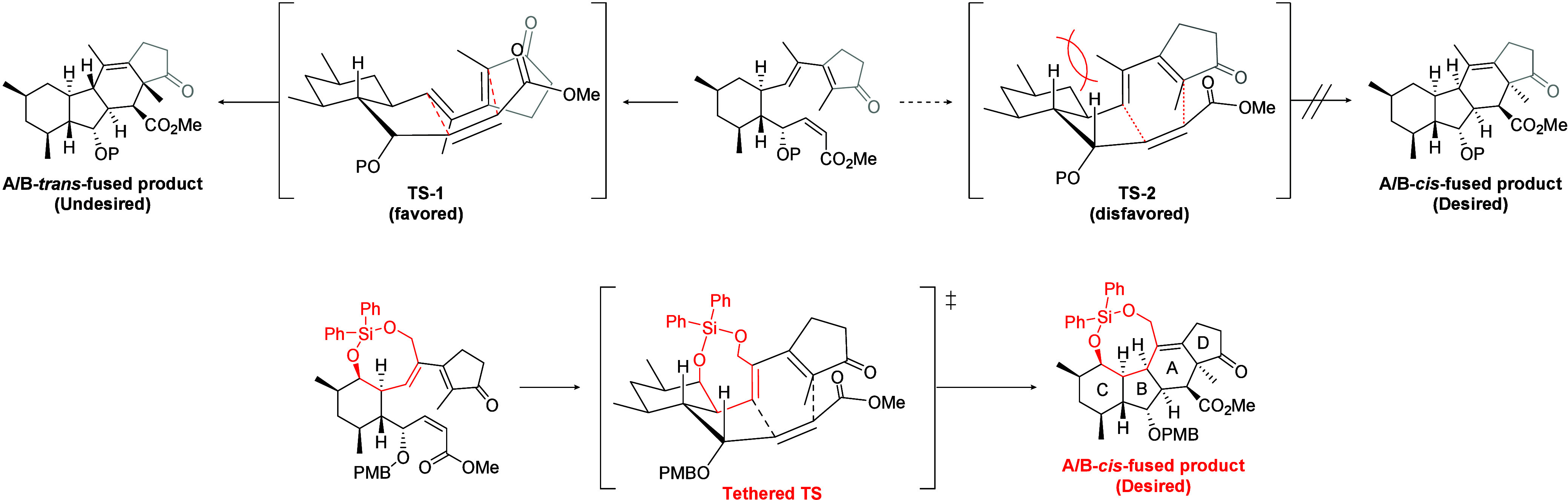
Tethered IMDA Strategy for the Synthesis of an A/B-*cis*-Fused Cyclopenta­[*b*]­fluorene (6/5/6/5) Ring System

Our retrosynthetic analysis of the A/B-*cis*-fused tetracyclic fragment (**5**) of embellicines is shown in Scheme 2. Target compound **5** could be obtained from tetracyclic intermediate **6** via deoxygenation of the silylene moiety and elaboration of the D-ring. The A/B-*cis*-fused tetracyclic skeleton of **6** could be constructed by using the tethered IMDA reaction described above. Cyclization precursor **7** could be prepared via a Stille coupling reaction between vinyl iodide **8** and cyclic vinyl stannane **9**. Silylene-bridged vinyl iodide **8** could be obtained by functional group manipulation of the methyl ketone moiety on C-ring intermediate **10**. Intermediate **10** is expected to be obtained by a Diels–Alder reaction between the previously reported dienophile **11** and siloxydiene **12**, and the subsequent diastereoselective hydrogenation of the olefin moiety.

**Scheme 2:**
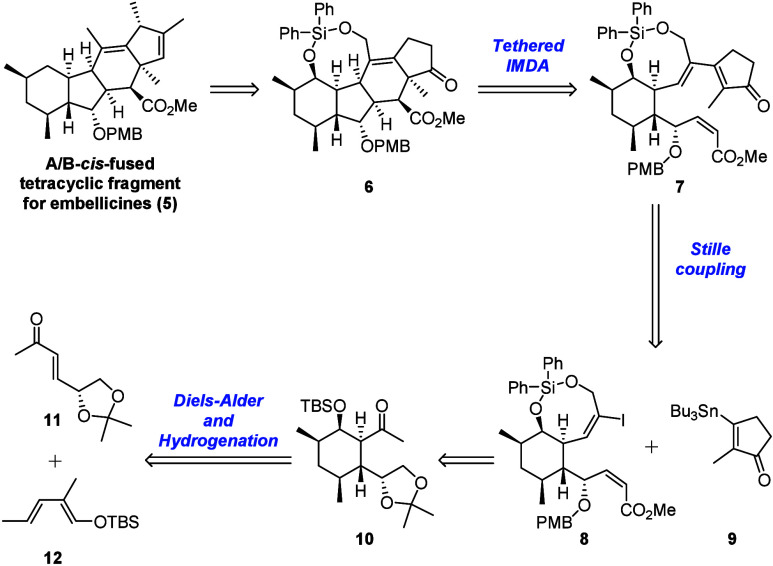
Retrosynthetic Analysis of the A/B-*cis*-Fused Tetracyclic Fragment of Embellicines

The construction of the C-ring was investigated first (Scheme 3). Chiral dienophile **11** was synthesized from l-ascorbic acid in a four-step reaction using a reported procedure.^9^ Siloxydiene **12** was prepared by treating commercially available (*E*)-2-methyl-2-pentenal **13** with TBSCl and NaI in the presence of Et_3_N. Resulting dienophile **11** and diene **12** underwent an asymmetric Diels–Alder reaction promoted by BF_3_·OEt_2_. The reaction proceeded stereoselectively, and cyclohexene **14** possesses a β-configured siloxy group, which is crucial for constructing the designed tethered structure. After the diastereoselective hydrogenation of **14**, the desired C-ring intermediate **10** was successfully obtained.

**Scheme 3:**
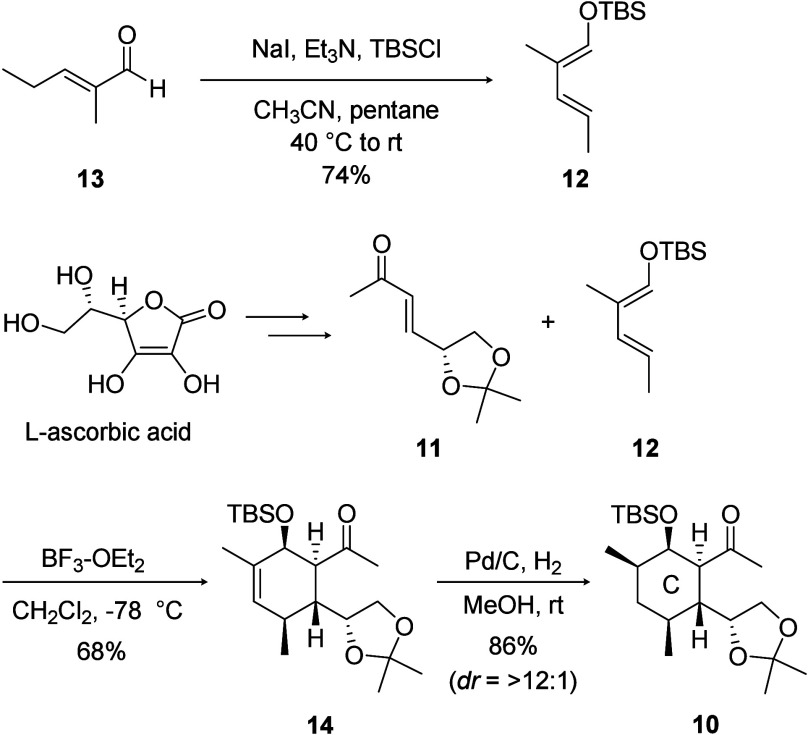
Synthesis of the C-Ring Fragment

Next, silylene-tethered vinyl iodide **8** was prepared (Scheme 4). The methyl ketone moiety of C-ring intermediate **10** was converted into enol phosphate,^10^ and the subsequent elimination^11^ and hydroxymethylation were conducted to afford propargyl alcohol **15**. After removal of the TBS group, hydrostannylation of the alkyne moiety,^12^ and subsequent iodination, nontethered vinyl iodide **18** was obtained. The primary hydroxyl group of **18** was protected with a TBDPS group, and the construction of the dienophile moiety was started. The acetonide group of **19** was removed by a treatment of propanedithiol and BF_3_·OEt_2_,^13^ and the resulting 1,2-diol was once more protected with a *p*-methoxybenzylidene group. The remaining secondary hydroxyl group on the C-ring of acetal **21** was protected with a TES group, and reductive cleavage of the *p*-methoxybenzylidene acetal using DIBAL yielded alcohol **23**. After Swern oxidation of the primary hydroxyl group of **23**, resulting aldehyde **24** was converted into (*Z*)-configured α,β-unsaturated ester **25** by the Horner–Wadsworth–Emmons (HWE) reaction using the Stille–Gennari reagent.^14^

**Scheme 4:**
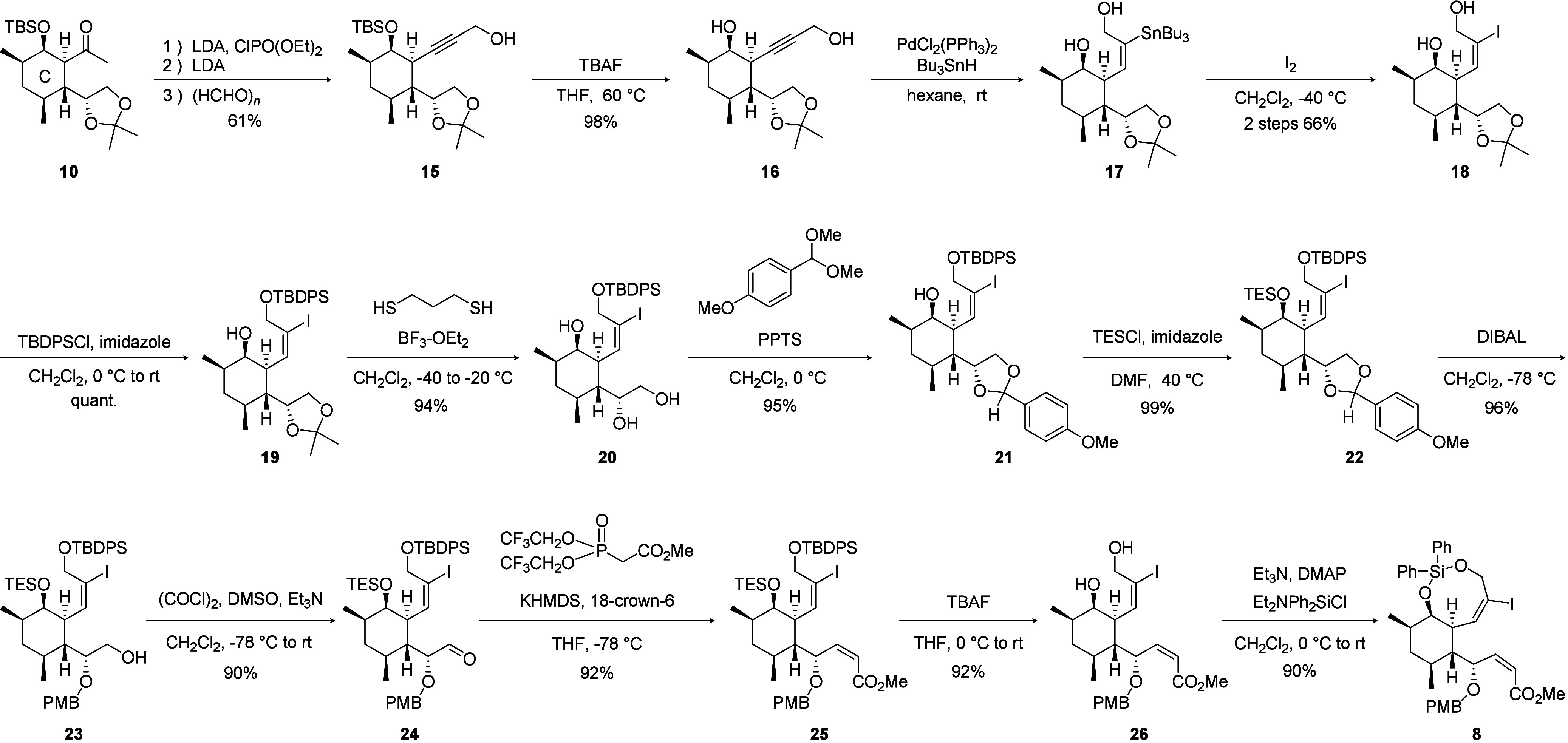
Synthesis of Tethered Vinyl Iodide (**8**)

Finally, an eight-membered silylene tether was introduced. The TES and TBDPS groups of α,β-unsaturated ester **25** were simultaneously removed by treatment with TBAF, giving diol **26**. Diol **26** was then treated with Et_2_NPh_2_SiCl under basic conditions,^15^ followed by the gradual addition of DMAP, resulting in the desired eight-membered silylene-bridged vinyl iodide **8** in high yield.^16^

Thus, the designed eight-membered silylene tether was successfully introduced, and a tetracyclic skeleton was constructed (Scheme 5). A Stille coupling reaction between silylene-bridged vinyl iodide **8** and independently prepared cyclic vinyl stannane **9** was conducted to obtain cyclization precursor **7**.^17^ Upon heating to 140 °C in xylene, the expected IMDA reaction proceeded smoothly, producing the desired A/B-*cis*-fused tetracyclic compound **6** in good yield with complete stereoselectivity. The A/B-*cis*-fused structure of compound **6** was elucidated by the NOESY correlation. It is noteworthy that the carbonyl group conjugated with the diene moiety of precursor **7** did not inhibit the IMDA reaction.

**Scheme 5:**
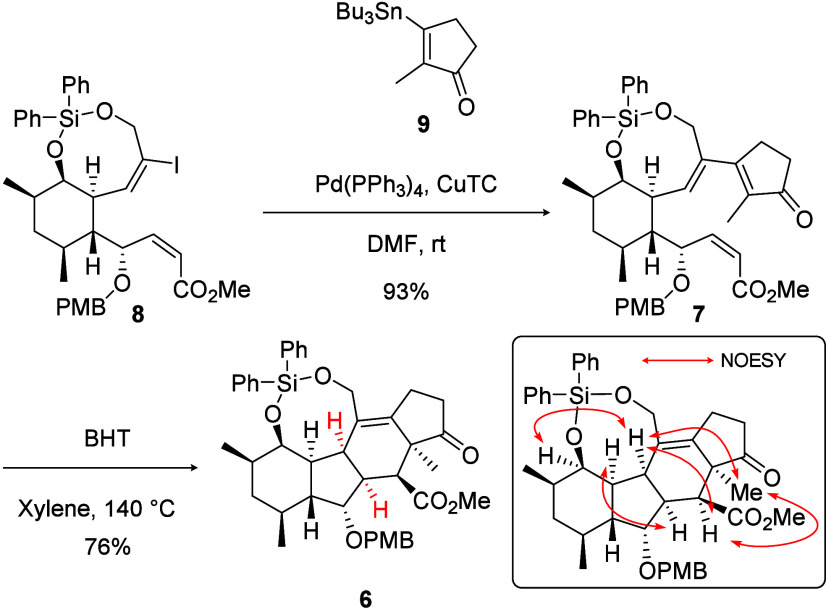
Construction of the A/B-*cis*-Fused Tetracyclic Skeleton

Next, deoxygenation of the silylene moiety and elaboration of the D-ring were investigated (Scheme 6). The cyclopentanone moiety of **6** was converted into enone **27** via α-selenenylation, followed by oxidative elimination. Conjugate addition of a methyl group to enone **27** using the Gilman reagent proceeded with complete stereoselectivity. Resulting β-methylketone **28** was further methylated at the α-position by successive treatment with LDA and methyl iodide. Cleavage of the silylene tether of bismethylated intermediate **29** was achieved using HF-pyridine to obtain diol **30**. The two resulting hydroxyl groups were sequentially removed by repeated Barton–McCombie deoxygenation to afford compound **34**. Finally, the cyclopentanone moiety of this compound was converted into enol triflate **35**, and palladium-catalyzed hydrogenolysis was conducted to obtain the desired tetracyclic fragment **5**. Interestingly, the use of electron-rich phosphine ligands, such as tricyclohexylphosphine or tributylphosphine, was essential for the reaction to proceed.^18^ The NOE correlations observed for the tetracyclic skeleton of compound **5** were consistent with those of embellicine A. In this research field, there has been some confusion about the process of structural elucidation of natural samples. For example, the structure of the cyclopenta­[*b*]­fluorene skeleton of phomapyrrolidone A was revised from the A/B-*cis* type to the A/B-*trans* type.^2^ Our results strongly suggest that embellicines possess an A/B-*cis*-fused tetracyclic skeleton.

**Scheme 6:**
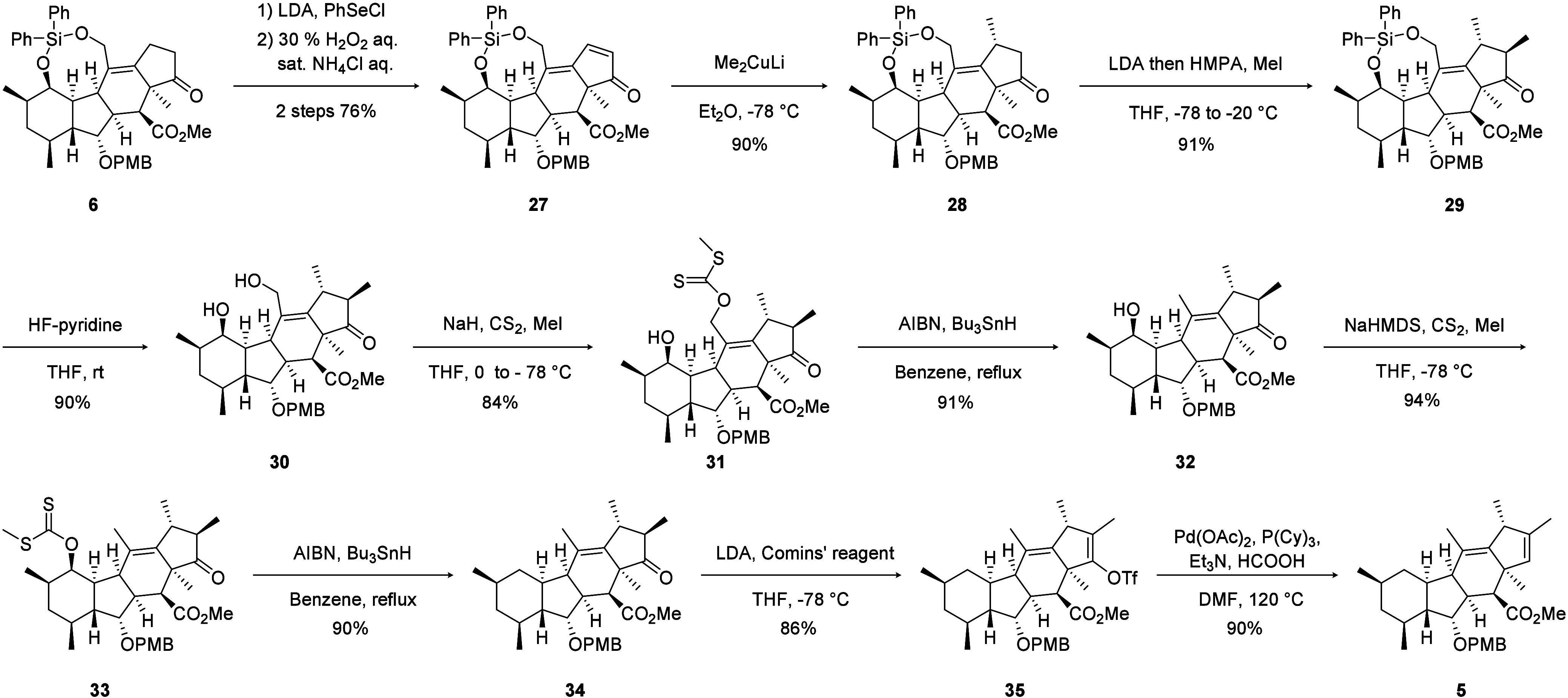
Synthesis of the Tetracyclic Skeleton of Embellicines

In conclusion, we achieved the first synthesis of an A/B-*cis*-fused cyclopenta­[*b*]­fluorene (6/5/6/5) fragment of embellicines via an IMDA reaction of an eight-membered silylene-tethered precursor with complete stereoselectivity. Further investigations of the total synthesis of embellicine A are currently in progress. In addition, we recently achieved the synthesis of an A/B-*trans*-fused tetracyclic ring system for phomapyrrolidones and ascomylactams by the nontethered IMDA reaction, and these results will be reported in the near future.

## Supplementary Material



## Data Availability

The data underlying this study are available in the published article and its Supporting Information.

## Notes

Detailed experimental procedures, characterization, and spectroscopic data (^1^H NMR, ^13^C NMR, NOESY, IR, and HRMS) (PDF)
