# Supplementary material for: Synthesis of an A/B-cis-Fused Cyclopenta[b]fluorene (6/5/6/5) Ring System for Embellicine A via the Eight-Membered Silylene-Tethered IMDA Reaction
Source: Org Lett. 2026 Jan 27;28(5):1510–4. doi: 10.1021/acs.orglett.5c04318 (PMC12888011; doi:10.1021/acs.orglett.5c04318)
Supplement: Supplementary file 1 [file ol5c04318_si_001.pdf]

Supporting Information

Synthesis of an A/B-*cis*-Fused Cyclopenta[*b*]fluorene (6/5/6/5) Ring System for Embellicine A via the Eight-Membered Silylene-Tethered IMDA Reaction

Yuya Sakai, Ryoma Murata, Akihiro Hirakawa, Yusuke Nakatani, Tsubasa Maeda, Yuki Kuwano, Shunya Morita, Hiromi Uchiro*

Faculty of Pharmaceutical Sciences, Tokyo University of Science,
6-3-1 Niijuku, Katsushika-ku, Tokyo 125-8585, Japan

uchiro@rs.tus.ac.jp

Table of Contents

Experimental Section------S2
NMR spectra------S40
NOESY NMR spectra------S69

General Procedures

All non-aqueous reactions were carried out under an atmosphere of argon in flame- or oven-dried glassware with magnetic stirring unless otherwise indicated. Dry diethyl ether (Et_2O) and tetrahydrofuran (THF) were purchased from Wako Pure Chemical Industries, Ltd. in anhydrous grade. Dichloromethane (CH_2Cl_2) was distilled from phosphorous pentoxide and then from calcium hydride before use. Acetonitrile (MeCN), *N*,*N*-dimethylformamide (DMF), dimethyl sulfoxide (DMSO), hexane, toluene, and xylenes were distilled from calcium hydride and stored over MS4A. Methanol (MeOH) was distilled from $Mg(OMe)_2$ and stored over MS3A. Benzene was distilled from sodium/benzophenone and stored over MS4A. Triethylamine (Et_3N), hexamethylphosphoric triamide (HMPA), diisopropylamine (*i*-Pr_2NH), and diisopropylethylamine (DIPEA) were distilled from calcium hydride and stored over potassium hydroxide. Reagents were purchased at the highest commercial quality and used without further purification unless otherwise stated.

Reactions were monitored by thin-layer chromatography (TLC) analysis on 0.25 mm silica gel plates (E. Merck, Silica Gel 60 F254). TLC plates were visualized by exposure to ultraviolet (UV) light (254 nm), and/or by staining with an acidic ethanol solution of *p*-anisaldehyde or basic aqueous potassium permanganate ($KMnO_4$) and then heating. Silica gel 60N (KANTO CHEMICAL CO., INC., spherical, neutral, 63–210 μm) was used for column chromatography. Preparative thin-layer chromatography (PTLC) separations were carried out on Wakogel® B–5F (Wako Pure Chemical Industries, Ltd.).

^1H and ^{13}C NMR spectra were recorded on a Bruker AVANCE600 and a JEOL JNM-

LD400 spectrometer that was operating at 400 or 600 MHz for ^{1}H NMR, 100 or 150 MHz for ^{13}C NMR. Chemical shifts were reported in parts per million (ppm) on the δ scale relative to residual $CDCl_3$ (δ = 7.26 for ^{1}H NMR and δ = 77.0 for ^{13}C NMR) as an internal reference. The following abbreviations are used to explain the multiplicities: s = singlet, d = doublet, t = triplet, q = quartet, m = multiplet, br = broad. Coupling constants (*J*) are reported in Hertz (Hz). Attenuated total reflectance (ATR)-FTIR spectra were recorded on a Perkin-Elmer 100 FTIR spectrometer. Absorbance frequencies are recorded in reciprocal centimeters (cm^{-1}). Optical rotations were measured using a JASCO P-1030 digital polarimeter using a 10 or 50 mm cell and the sodium D line (589 nm). The operated temperature, solvent, and concentration were indicated in each compound.

High-resolution mass spectroscopy (HRMS) was performed on a Varian 910-Fourier-transform mass spectrometer by using electrospray ionization (ESI).

Synthetic Procedures and Characterizations of Compounds

tert-Butyldimethyl(((1*E*,3*E*)-2-methylpenta-1,3-dien-1-yl)oxy)silane (12)

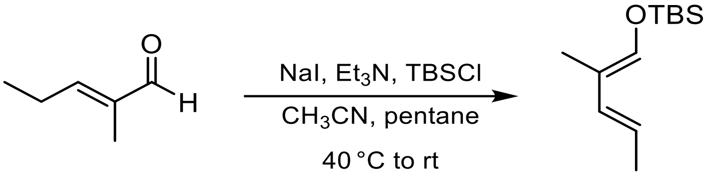

Sodium iodide (14.3 g, 95.4 mmol) was dried under reduced pressure at 120 ℃ for 5 h, then dissolved in anhydrous acetonitrile (10 mL) at 0 ℃. To the mixture, a solution of the starting material (10.0 g, 101.9 mmol) in anhydrous acetonitrile (2 mL) and triethylamine (23.0 mL, 163.7 mmol) were added followed by pentane (16 mL) and TBSCl (15.4 g, 101.9 mmol). The reaction mixture was stirred vigorously at 40 ℃ in a water bath for 2 h and at room temperature overnight. The resulting mixture was filtered through a celite pad and washed with hexane. The filtrate was concentrated under reduced pressure. The residue was purified by column chromatography (silica gel, hexane/EtOAc = 100:1) to give the title compound (16.0 g, 74% yield) as a colorless oil.

IR (ATR, neat) ν_{max} 2956, 2930, 2858, 1697, 1650, 1628, 1472, 1252, 1171, 835, 778, 675 cm^{-1};

^{1}H NMR (600 MHz, $CDCl_3$) δ : 6.33 (1H, t, *J* = 0.6 Hz), 5.98 (1H, dd, *J* = 15, 1.2 Hz), 5.34-5.49 (1H, m), 1.76 (3H, dd, *J* = 7.2, 1.2 Hz), 1.70 (3H, s), 0.93 (9H, s), 0.14 (6H, s);

^{13}C NMR (150 MHz, $CDCl_3$) δ : 139.4, 131.0, 119.6, 117.9, 25.6, 18.3, 18.2, 9.4, -5.3.

ESI-MS(positive) for calcd for $C_{12}H_{25}OSi$ $[M+H]^+$ 213.1669, found 213.1674.

1-((1*S*,2*S*,5*S*,6*R*)-2-((*tert*-Butyldimethylsilyl)oxy)-6-((*R*)-2,2-dimethyl-1,3-dioxolan-4-yl)-3,5-dimethylcyclohex-3-en-1-yl)ethan-1-one (14)

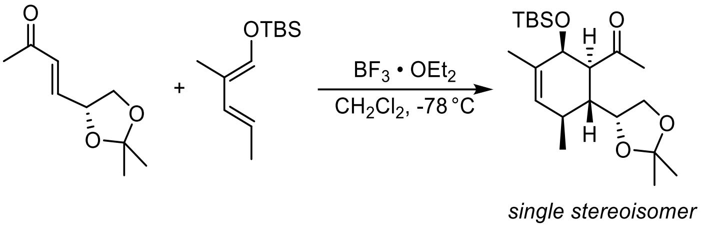

Dienophile (2.77 g, 16.3 mmol) and diene (6.9 g, 32.6 mmol) in anhydrous CH_2Cl_2 (162 mL) were cooled to -78 ℃. After stirring for 30 min, boron trifluoride-diethyl ether complex (2.0 mL, 16.3 mmol) was added dropwise and stirred for 30 min. The reaction was quenched with saturated aqueous $NaHCO_3$ at -78 ℃. The reaction mixture was extracted with CH_2Cl_2. The organic layer was washed with water and brine, dried over anhydrous Na_2SO_4, and concentrated *in vacuo*. The residue was purified by column chromatography (silica gel, hexane/EtOAc = 30:1) to give the titled compound (4.23 g, 68% yield) as a colorless oil with recovered dienophile (20%).

$[\alpha]_D{}^{23}$ = - 1.6 (c = 0.84, $CHCl_3$);

IR (ATR, neat) ν_{max} 2956, 2930, 2858, 1718, 1696, 1472, 1462, 1369, 1251, 1214, 1160, 1085, 1051, 1005, 878, 834, 773, 677 cm^{-1};

^1H NMR (600 MHz, $CDCl_3$) δ : 5.30 (1H, m), 4.34 (1H, d, J = 3.6 Hz), 4.01 (1H, dd, J = 13.2, 7.2 Hz), 3.95 (1H, dd, J = 7.8, 6.0 Hz), 3.62 (1H, t, J = 7.2 Hz), 2.86 (1H, dd, J = 6.6, 4.8 Hz), 2.21 (3H, s), 2.19-2.18 (1H, m), 1.92 (1H, s), 1.78 (3H, s), 1.41 (3H, s), 1.31 (3H, s), 1.05 (3H, d, J = 7.2 Hz), 0.88 (9H, s), 0.07 (3H, s), 0.06 (3H, s);

^{13}C NMR (150 MHz, $CDCl_3$) δ : 210.0, 135.6, 128.5, 108.8, 77.4, 68.8, 68.1, 54.8, 42.7, 31.5, 30.5, 26.6, 25.8, 25.3, 22.0, 20.3, 18.0, -4.71;

ESI-MS(positive) for calcd for $C_{21}H_{39}O_4Si$ $[M+H]^+$ 383.2612, found 383.2613.

1-((1*S*,2*R*,3*R*,5*S*,6*R*)-2-((*tert*-Butyldimethylsilyl)oxy)-6-((*R*)-2,2-dimethyl-1,3-dioxolan-4-yl)-3,5-dimethylcyclohexyl)ethan-1-one (10)

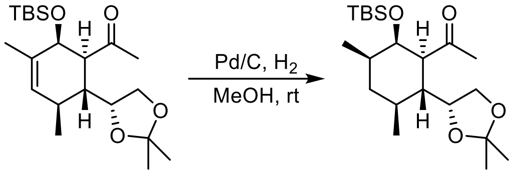

A mixture of the starting material (6.42 g, 16.8 mmol) and 5% Pd/C (1.8 g, 0.84 mmol) in anhydrous MeOH (168 mL) was stirred under a H_2 atmosphere. After stirring for 3 h, the reaction mixture was filtered through a celite pad and washed with EtOAc. The filtrate was concentrated under reduced pressure. The residue was purified by column chromatography (silica gel, hexane/EtOAc = 20:1) to give the titled compound (5.54 g, 86% yield) as a colorless oil.

$[\alpha]_D^{19}$ = + 41.7 (c = 0.25, $CHCl_3$);

IR (ATR, neat) ν_{max} 2955, 2928, 2855, 1718, 1471, 1462, 1377, 1368, 1249, 1216, 1062, 1032, 839, 828, 773, 672 cm^{-1};

^1H NMR (600 MHz, $CDCl_3$) δ : 3.94 (1H, s), 3.90 (1H, t, J = 6.0 Hz), 3.83 (1H, m), 3.64 (1H, t, J = 7.8 Hz), 2.65 (1H, dd, J = 11.4, 1.8 Hz), 2.14 (3H, s), 2.10-2.06 (1H, m), 1.54-1.52 (1H, m), 1.38 (1H, m), 1.32-1.21 (8H, m), 0.89-0.88 (15H, m), -0.03 (3H, s), -0.13 (3H, s);

^{13}C NMR (150 MHz, $CDCl_3$) δ : 206.6, 108.2, 78.9, 71.5, 70.6, 57.8, 39.0, 37.8, 35.9, 29.8, 26.2, 25.9, 25.5, 21.1, 19.7, 18.4, -3.2, -3.7;

ESI-MS(positive) for calcd for $C_{21}H_{44}NO_4Si$ $[M+NH_4]^+$ 402.3034, found 402.3034.

3-((1*R*,2*R*,3*R*,5*S*,6*R*)-2-((*tert*-Butyldimethylsilyl)oxy)-6-((*R*)-2,2-dimethyl-1,3-dioxolan-4-yl)-3,5-dimethylcyclohexyl)prop-2-yn-1-ol (15)

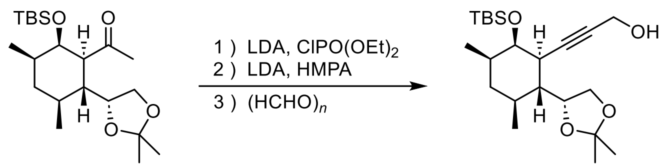

A solution of lithium diisopropylamide (LDA) (1.4 M in THF, 6 mL, 8.45 mmol) was cooled to -78 °C. Then, the solution of material (2.5 g, 6.5 mmol) in anhydrous THF (5 mL) was added dropwise. After stirring for 30 min, diethyl chlorophosphate (1.41 mL, 9.75 mmol) was added. The mixture was stirred at -60 °C for 30 min. The reaction mixture was then cooled to -78 °C, and a solution of lithium diisopropylamide (LDA) (2.8 M in THF, 6 mL, 16.9 mmol) was added dropwise. After stirring for 1 h, hexamethylphosphoric triamide (4 mL) and paraformaldehyde (660 mg, 21.9 mmol) were added, and the mixture was stirred at room temperature for 2 h. The reaction mixture was quenched with saturated aqueous NH_4Cl at 0 °C. The mixture was extracted with EtOAc. The organic layer was washed with water and brine, dried over anhydrous Na_2SO_4, and concentrated *in vacuo*. The residue was purified by column chromatography (silica gel, hexane/EtOAc = 20:1) to give the titled compound (1.57 g, 61% yield) as a yellow oil.

$[\alpha]_D^{22}$ = - 32.3 (c = 0.21, $CHCl_3$);

IR (ATR, $CHCl_3$) ν_{max} 3424, 2954, 2928, 2880, 2856, 1472, 1461, 1380, 1369, 1250, 1211, 1151, 1130, 1060, 1026, 919, 826, 811, 754, 666 cm^{-1};

^{1}H NMR (600 MHz, $CDCl_3$) δ : 4.61 (1H, t, *J* = 7.8 Hz), 4.21-4.14 (3H, m), 3.97 (1H, t, *J* = 7.8 Hz), 3.86 (1H, s), 2.86 (1H, m), 2.54 (1H, m), 1.70 (1H, t, *J* = 10.8 Hz), 1.57 (3H, s), 1.53-1.48 (2H, m), 1.33 (3H, s), 1.31-1.25 (2H, m), 1.00 (3H, d, *J* = 6.0 Hz), 0.93 (9H, s), 0.87 (3H, d, *J* = 7.2 Hz), 0.16 (3H, s), 0.073 (3H, s);

^{13}C NMR (150 MHz, $CDCl_3$) δ : 109.3, 88.9, 80.6, 75.0, 74.8, 67.5, 51.4, 41.7, 37.8, 37.5, 37.0, 34.0, 26.2, 25.8, 24.0, 20.8, 19.3, 18.6, -3.31, -3.74;

ESI-MS(positive) for calcd for $C_{22}H_{40}NaO_4Si$ $[M+Na]^+$ 419.2588, found 419.2588.

(1*R*,2*R*,3*R*,4*S*,6*R*)-3-((*R*)-2,2-Dimethyl-1,3-dioxolan-4-yl)-2-(3-hydroxyprop-1-yn-1-yl)-4,6-dimethylcyclohexan-1-ol (16)

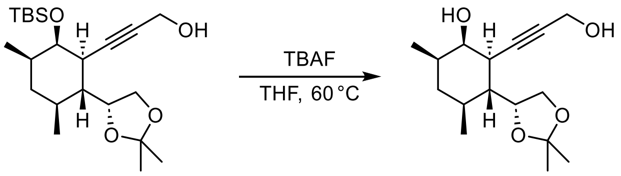

To a stirred solution of the starting material (6.35 g, 16 mmol) in anhydrous THF (160 mL) at 0 ℃ was added tetrabutylammonium fluoride (1.0 M in THF, 80 mL, 80 mmol. The resulting mixture was warmed to 60 ℃ in a water bath and stirred for 24 h. The reaction mixture was quenched with saturated aqueous NH_4Cl at 0 ℃. The mixture was extracted with EtOAc. The organic layer was washed with water and brine, dried over anhydrous Na_2SO_4, and concentrated *in vacuo*. The residue was purified by column chromatography (silica gel, hexane/EtOAc = 10:1) to give the titled compound (4.43 g, 98% yield) as a colorless oil.

$[\alpha]_D^{22}$ = - 72.0 (*c* = 0.58, $CHCl_3$);

IR (ATR, neat) v_{max} 3399, 2955, 2912, 2873, 2242, 1642, 1455, 1369, 1245, 1211, 1151, 1050, 1013, 992, 954, 890, 863, 791, 511 cm^{-1};

^1H NMR (600 MHz, $CDCl_3$) δ : 4.57 (1H, t, *J* = 7.8 Hz), 4.25-4.22 (2H, m), 4.20 (1H, t, *J* = 7.8 Hz), 3.97 (1H, t, *J* = 7.8 Hz), 3.78 (1H, m), 2.61 (1H, m), 2.54 (1H, m), 1.90 (1H, s), 1.65 (1H, t, *J* = 11.4 Hz), 1.59-1.46 (2H, m), 1.53 (3H, s), 1.38-1.32 (1H, m), 1.34 (3H, s), 1.31-1.24 (1H, m), 1.01 (3H, d, *J* = 6.0 Hz), 0.99 (3H, d, *J* = 7.2 Hz);

^{13}C NMR (150 MHz, $CDCl_3$) δ : 109.1, 87.7, 81.5, 74.9, 73.2, 67.5, 51.3, 42.1, 37.1, 36.9, 35.3, 33.9, 26.0, 24.4, 20.6, 18.2;

ESI-MS(positive) for calcd for $C_{16}H_{26}NaO_4$ $[M+Na]^+$ 305.1723, found 305.1726.

(1*R*,2*R*,3*R*,4*S*,6*R*)-3-((*R*)-2,2-Dimethyl-1,3-dioxolan-4-yl)-2-((*E*)-3-hydroxy-2-iodoprop-1-en-1-yl)-4,6-dimethylcyclohexan-1-ol (18)

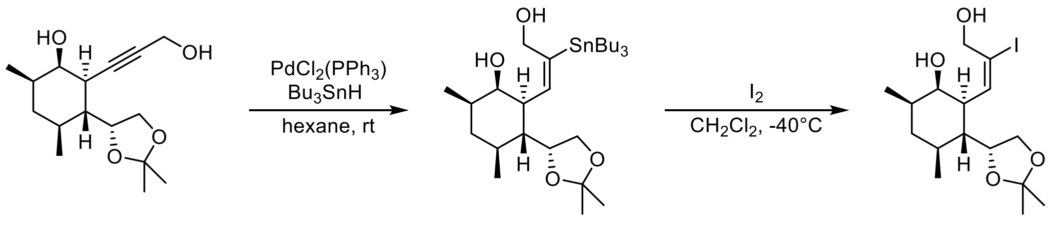

To a stirred solution of the starting material (4.07 g, 14.4 mmol) and $PdCl_2(PPh_3)_2$ (1.0 g, 1.44 mmol) in anhydrous hexane (143.8 mL) was added Bu_3SnH (9.3 mL, 34.6 mmol) dropwise and stirred at room temperature overnight. The reaction mixture was filtered through a celite pad and washed with EtOAc. The filtrate was concentrated under reduced pressure, and the resulting residue was used in the next reaction without purification.

To the stirred clude mixture in anhydrous CH_2Cl_2 (215.8 mL) at -40 °C was added Iodine (3.0 g, 23.7 mmol) and stirred for 30 min. The reaction mixture was quenched with 5% aqueous solution of sodium thiosulfate and 8 M aqueous solution of potassium fluoride, then the mixture was stirred for 30 min at room temperature. The mixture was extracted with CH_2Cl_2. The organic layer was washed with water and brine, dried over anhydrous Na_2SO_4, and concentrated *in vacuo*. The residue was purified by column chromatography (silica gel, hexane/EtOAc = 6:1) to give the titled compound (3.89 g, 66% yield for 2 steps) as a colorless oil.

$[\alpha]_D^{23}$ = - 102.7 (*c* = 0.14, $CHCl_3$);

IR (ATR, neat, tablet) ν_{max} 3323, 2965, 2913, 2873, 1630, 1456, 1370, 1246, 1211, 1154, 1042, 990, 951, 902, 857, 509 cm^{-1};

^1H NMR (600 MHz, $CDCl_3$) δ : 6.52 (1H, d, *J* = 10.8 Hz), 4.33 (1H, d, *J* = 12.6 Hz), 4.30 (1H, dt, *J* = 7.2, 3.0 Hz), 4.04 (1H, dd, *J* = 12.6, 8.4 Hz), 3.87 (2H, m), 3.58 (1H, s), 2.64 (1H, dd, *J* = 9.6, 4.2 Hz), 2.57 (1H, dt, *J* = 10.8, 3.0 Hz), 1.63-1.54 (3H, m), 1.46 (3H, s), 1.41 (1H, d, *J* = 4.2 Hz), 1.31 (3H, s), 1.33-1.26 (2H, m), 1.01 (3H, d, *J* = 6.6 Hz), 0.93 (3H, d, *J* = 6.6 Hz);

^{13}C NMR (150 MHz, $CDCl_3$) δ : 147.8, 107.9, 101.4, 76.3, 75.8, 66.1, 65.7, 45.3, 42.3, 36.2, 36.0, 33.5, 26.1, 24.3, 20.8, 17.8;

ESI-MS(positive) for calcd for $C_{16}H_{27}INaO_4$ $[M+Na]^+$ 433.0846, found 433.0838.

(1*R*,2*R*,3*R*,4*S*,6*R*)-2-((*E*)-*tert*-Butyldiphenylsillyl)oxy)-2-iodoprop-1-en-1-yl)-3-((*R*)-2,2-dimethyl-1,3-dioxolan-4-yl)-4,6-dimethylcyclohexan-1-ol (19)

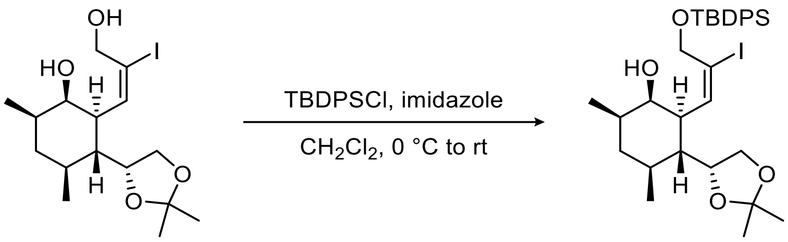

To a stirred solution of diol (3.57 g, 8.7 mmol) and imidazole (1.18 g, 17.4 mmol) in CH_2Cl_2 (29 mL) at 0 °C was added *tert*-butyldiphenylchlorosilane (3.39 mL, 13 mmol) dropwise. The mixture was stirred at room temperature for 1 h. The reaction mixture was quenched with saturated aqueous $NaHCO_3$ at 0 °C. The mixture was extracted with CH_2Cl_2. The organic layer was washed with water and brine, dried over anhydrous Na_2SO_4, and concentrated *in vacuo*. The crude residue was purified by column chromatography (silica gel, hexane/EtOAc = 20:1) to give the titled compound (5.65 g, quantitative yield) as a colorless oil.

$[\alpha]_D{}^{23}$ = - 72.3 (c = 0.20, $CHCl_3$);

IR (ATR, $CHCl_3$) ν_{max} 3478, 3071, 3049, 2955, 2929, 2858, 1626, 1589, 1473, 1461, 1427, 1370, 1211, 1154, 1111, 1055, 992, 824, 741, 702, 615, 504 cm^{-1};

^1H NMR (600 MHz, $CDCl_3$) δ : 7.71-7.69 (4H, m), 7.43-7.38 (6H, m), 6.43 (1H, d, J = 10.2 Hz), 4.28 (1H, dd, J = 12.6, 1.2 Hz), 4.16 (1H, dd, J = 12.6, 1.2 Hz), 4.14 (1H, m), 3.72 (1H, dd, J = 7.8, 6.0 Hz), 3.61 (1H, dd, J = 8.4, 7.8 Hz), 3.42 (1H, brs), 2.23-2.19 (1H, m), 1.71-1.67 (1H, dt, J = 11.4, 3.0 Hz), 1.39-1.36 (2H, m), 1.32 (3H, s), 1.27 (3H, s), 1.29-1.18 (2H, m), 1.08 (9H, s), 0.99 (3H, d, J = 6.0 Hz), 0.86 (3H, d, J = 6.6 Hz);

^{13}C NMR (150 MHz, $CDCl_3$) δ : 146.2, 135.7, 136.6, 133.1, 133.0, 129.9, 127.8, 127.7, 107.9, 100.7, 76.8, 75.2, 66.2, 46.6, 41.1, 36.8, 35.9, 33.6, 26.7, 26.5, 25.0, 21.1, 19.3, 17.7;

ESI-MS(positive) for calcd for $C_{32}H_{49}INO_4Si$ $[M+NH_4]^+$ 666.2470, found 666.2470.

(*R*)-1-((1*R*,2*R*,3*R*,4*R*,6*S*)-2-((*E*)-3-((*tert*-Butyldiphenylsilyl)oxy)-2-iodoprop-1-en-1-yl)-3-hydroxy-4,6-dimethylcyclohexyl)ethane-1,2-diol (20)

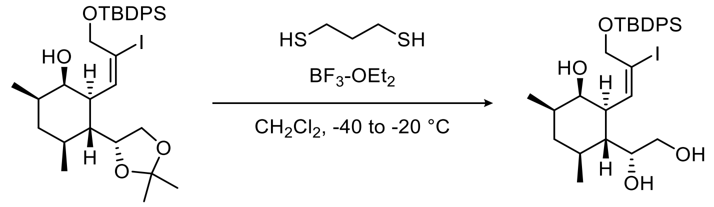

To a stirred solution of starting material (5.65 g, 8.7 mmol) and 1,3-propanedithiol (3.48 mL, 34.8 mmol) in CH_2Cl_2 (87 mL) at -40 ℃ was added $BF_3 \cdot OEt_2$ (0.11 mL, 0.87 mmol) dropwise. The reaction mixture was stirred at -20 ℃ for 1 h. The reaction mixture was quenched with saturated aqueous $NaHCO_3$ at -40 ℃. The mixture was extracted with CH_2Cl_2. The organic layer was washed with water and brine, dried over anhydrous Na_2SO_4, and concentrated *in vacuo*. The crude residue was purified by column chromatography (silica gel, hexane/EtOAc = 2:1) to give the titled compound (4.97 g, 94% yield) as a colorless oil.

$[\alpha]_D{}^{23}$ = - 49.4 (*c* = 2.9, $CHCl_3$);

IR (ATR, neat) ν_{max} 3414, 3071, 3049, 2955, 2929, 2857, 1626, 1589, 1472, 1461, 1427, 1360, 1216, 1109, 1054, 994, 823, 757, 742, 701, 614, 504 cm^{-1};

^1H NMR (600 MHz, $CDCl_3$) δ : 7.72-7.71 (4H, m), 7.41-7.36 (6H, m), 6.39 (1H, d, *J* = 10.8 Hz), 4.32-4.26 (2H, m), 3.94 (1H, d, *J* = 9.0 Hz), 3.68 (1H, t, *J* = 9.6 Hz), 3.42 (1H, s), 3.38 (1H, d, *J* = 10.8 Hz), 2.45 (1H, dt, *J* = 10.8, 2.4 Hz), 1.45-1.37 (3H, m), 1.33-1.29 (1H, m), 1.19 (1H, q, *J* = 9.5 Hz), 1.08 (9H, s), 0.98 (3H, d, *J* = 6.0 Hz), 0.86 (3H, d, *J* = 6.6 Hz);

^{13}C NMR (150 MHz, $CDCl_3$) δ : 146.4, 135.8, 135.7, 133.0, 132.9, 129.9, 129.8, 127.8, 127.7, 101.6, 75.0, 73.1, 66.4, 66.0, 45.3, 43.5, 36.6, 35.9, 33.1, 20.8, 19.3, 17.8;

ESI-MS(positive) for calcd for $C_{29}H_{41}IKO_4Si$ $[M+K]^+$ 647.1450, found 647.1444.

(1*R*,2*R*,3*R*,4*S*,6*R*)-2-((*E*)-*tert*-Butyldiphenylsilyl)oxy)-2-iodoprop-1-en-1-yl)-3-((4*R*)-2-(4-methoxyphenyl)-1,3-dioxolan-4-yl)-4,6-dimethylcyclohexan-1-ol (21)

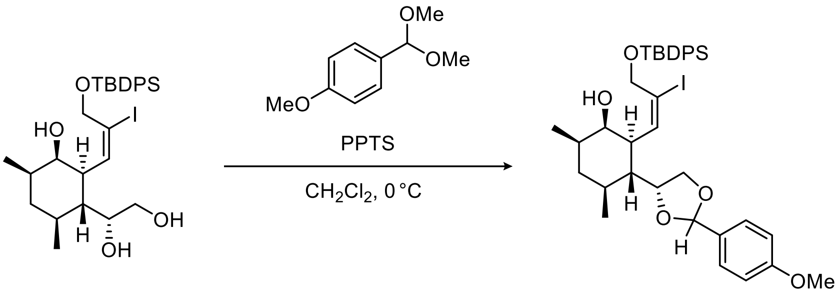

To a stirred solution of triol (4.63 g, 7.6 mmol) in CH_2Cl_2 (76 mL) at 0 ℃ were added *p*-anisaldehyde dimethyl acetal (2.68 mL, 15.2 mmol) and pyridinium *p*-toluenesulfonate (0.19 g, 0.76 mmol). The resulting mixture was stirred for 1 h at the same temperature. The reaction mixture was quenched with saturated aqueous $NaHCO_3$ at 0 ℃. The mixture was extracted with CH_2Cl_2. The organic layer was washed with water and brine, dried over anhydrous Na_2SO_4, and concentrated *in vacuo*. The residue was purified by column chromatography (silica gel, hexane/EtOAc = 10:1) to give the titled compound (5.25 g, 95% yield) as a colorless oil.

$[\alpha]_D^{23}$ = - 51.8 (*c* = 1.04, $CHCl_3$);

IR (ATR, $CHCl_3$) ν_{max} 3496, 3070, 3048, 2955, 2928, 2856, 1724, 1614, 1516, 1462, 1428, 1382, 1304, 1248, 1170, 1111, 1079, 1035, 825, 741, 702, 504 cm^{-1};

^1H NMR (600 MHz, $CDCl_3$) δ : 7.65-7.63 (4H, m), 7.46-7.36 (6H, m), 7.35 (2H, m), 6.81 (2H, m), 6.47 (1H, d, *J* = 10.2 Hz), 5.60 (1H, s), 4.32 (1H, dd, *J* = 12.6, 0.6 Hz), 4.27-4.21

(1H, m), 3.92 (1H, d, J = 12.6 Hz), 3.74 (3H, s), 3.78-3.72 (2H, m), 3.40 (1H, brs), 2.20 (1H, dt, J = 11.1, 2.4 Hz), 1.82 (1H, dt, J = 11.0, 1.8 Hz), 1.49-1.17 (4H, m), 1.04 (9H, s), 1.02 (3H, d, J = 6.6 Hz), 0.85 (3H, d, J = 6.6 Hz);

^{13}C NMR (100 MHz, CDCl$_3$) δ : 160.1, 145.5, 135.6, 135.5, 133.1, 133.0, 129.8, 129.2, 128.0, 127.7, 127.6, 113.6, 102.9, 101.4, 78.4, 75.0, 55.3, 46.7, 40.9, 36.8, 35.9, 33.2, 26.7, 21.2, 19.2, 17.7;

ESI-MS(positive) for calcd for $C_{37}H_{48}IO_5Si$ [M+H]$^+$ 727.2310, found 727.2310.

tert-Butyl((((_E_)-20-ido-3-((1_R_,2_R_,3_S_,5_R_,6_R_)-2-((4_R_)-2-(4-methoxyphenyl)-1,3-dioxolan-4-yl)-3,5-dimethyl-6-((triethylsilyl)oxy)cyclohexyl)allyl)oxy)diphenylsilane (22)

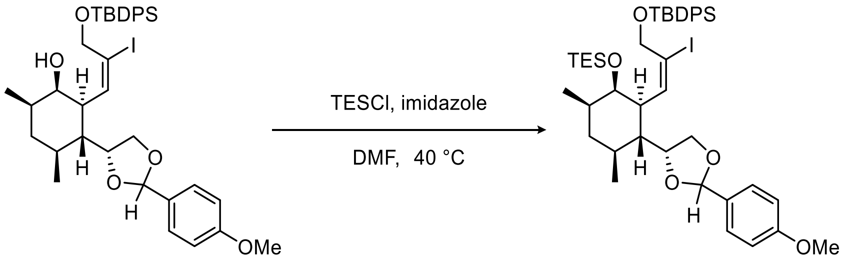

To a stirred solution of the starting material (2.83 g, 3.9 mmol) and imidazole (0.53 g, 7.8 mmol) in DMF (29 mL) at 0 °C was added triethylsilyl chloride (1.03 mL, 5.85 mmol) dropwise. The reaction mixture was stirred at 40 °C in a water bath for 1 h. The reaction mixture was quenched with saturated aqueous $NaHCO_3$ at 0 °C. The mixture was extracted with EtOAc. The organic layer was washed with water and brine, dried over anhydrous Na_2SO_4, and concentrated *in vacuo*. The crude residue was purified by column chromatography (silica gel, hexane/EtOAc = 30:1) to give the titled compound (3.2 g, 99%

yield) as a colorless oil.

$[\alpha]_D{}^{23}$ = - 38.9 (c = 0.12, $CHCl_3$);

IR (ATR, $CHCl_3$) ν_{max} 3313, 3071, 2954, 2928, 2874, 2856, 1736, 1615, 1589, 1510, 1461, 1427, 1380, 1303, 1248, 1169, 1068, 1055, 1006, 969, 824, 804, 737, 700, 614, 502 cm^{-1};

^1H NMR (600 MHz, $CDCl_3$) δ : 7.66-7.63 (4H, m), 7.41-7.36 (6H, m), 7.34 (2H, m), 6.79 (2H, m), 6.32 (1H, d, J = 10.2 Hz), 5.59 (1H, s), 4.26-4.23 (1H, dt, J = 7.8, 1.8 Hz), 4.21 (1H, d, J = 13.2 Hz), 4.29 (1H, d, J = 13.2 Hz), 3.74 (3H, s), 3.73-3.70 (2H, m), 3.61(1H, brs), 2.16 (1H, dt, J = 10.8, 1.8 Hz), 1.89 (1H, dt, J = 10.8, 1.8 Hz), 1.68-1.60 (1H, m), 1.49-1.41 (1H, m), 1.23-1.20 (2H, m), 1.06 (9H, s), 1.03 (3H, d, J = 6.0 Hz), 0.93 (9H, t, J = 7.8 Hz), 0.84 (3H, d, J = 6.6 Hz), 0.66-0.58 (6H, m);

^{13}C NMR (100 MHz, $CDCl_3$) δ : 160.1, 146.5, 135.6, 133.1, 129.8, 129.2, 128.0, 127.7, 113.6, 102.9, 101.0, 78.7, 77.6, 66.4, 65.9, 55.2, 47.8, 40.9, 37.2, 37.1, 33.2, 26.7, 21.4, 19.3, 18.6, 7.2, 5.5;

ESI-MS(positive) for calcd for $C_{43}H_{62}IO_5Si_2$ $[M+H]^+$ 841.3175, found 841.3172.

(*R*)-2-((1*R*,2*R*,3*R*,4*R*,6*S*)-2-((*E*)-3-((*tert*-Butyldiphenylsilyl)oxy)-2-iodoprop-1-en-1-yl)-4,6-dimethyl-3-((triethylsilyl)oxy)cyclohexyl)-2-((4-methoxybenzyl)oxy)ethan-1-ol (23)

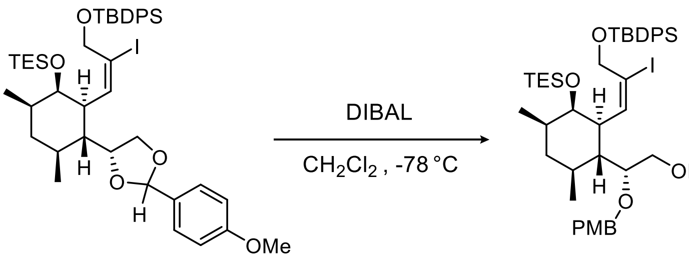

To a stirred solution of the starting material (4.7 g, 5.58 mmol) in THF (55.8 mL) at -

78 ℃ was added a solution of diisobutylaluminum Hydride (1.0 M in hexane, 16.5 mL, 16.5 mmol) dropwise. The resulting mixture was stirred for 1 h at the same temperature. The reaction mixture was quenched with MeOH and saturated aqueous solution of potassium sodium tartrate at -78 ℃. The mixture was extracted with CH_2Cl_2. The organic layer was washed with water and brine, dried over anhydrous Na_2SO_4, and concentrated *in vacuo*. The residue was purified by column chromatography (silica gel, hexane/EtOAc = 15:1) to give the titled compound (4.51 g, 96% yield) as a colorless oil.

$[\alpha]_D^{22}$ = - 35.1 (*c* = 0.82, $CHCl_3$);

IR (ATR, neat) ν_{max} 3483, 3071, 2953, 2931, 2910, 2874, 1739, 1612, 1587, 1512, 1461, 1427, 1371, 1243, 1047, 818, 737, 701, 503 cm^{-1};

^{1}H NMR (600 MHz, $CDCl_3$) δ : 7.67 (4H, m), 7.46-7.38 (6H, m), 7.19 (2H, d, *J* = 8.4 Hz), 6.83 (2H, d, *J* = 8.4 Hz), 6.17 (1H, d, *J* = 10.2 Hz), 4.53 (1H, d, *J* = 11.4 Hz), 4.35 (2H, dd, 15, 5.1 Hz), 4.08 (1H, d, *J* = 12.6 Hz), 3.78 (3H, s), 3.60 (2H, m), 3.53 (1H, m), 3.29 (1H, m), 2.18 (1H, dt, *J* = 11.0, 1.8 Hz), 1.72 (1H, t, *J* = 10.8 Hz), 1.66 (1H, m), 1.25 (4H, m), 1.07 (9H, s), 1.01 (9H, t, *J* = 8.4 Hz), 0.98 (3H, d, *J* = 6.6 Hz), 0.83 (3H, d, *J* = 6.0 Hz), 0.66 (6H, m);

^{13}C NMR (100 MHz, $CDCl_3$) δ : 159.1, 146.3, 135.6, 133.1, 132.9, 127.6, 113.8, 101.6, 80.6, 71.3, 66.3, 62.8, 55.1, 47.6, 40.9, 37.3, 36.9, 33.2, 26.6, 21.3, 19.2, 18.6, 7.2, 5.4;

ESI-MS(positive) for calcd for $C_{43}H_{67}INO_5Si_2$ $[M+NH_4]^+$ 860.3597, found 860.3601.

(*R*)-2-((1*R*,2*R*,3*R*,4*R*,6*S*)-2-((*E*)-3-((*tert*-Butyldiphenylsilyl)oxy)-2-iodoprop-1-en-1-yl)-4,6-dimethyl-3-((triethylsilyl)oxy)cyclohexyl)-2-((4-methoxybenzyl)oxy)acetaldehyde (24)

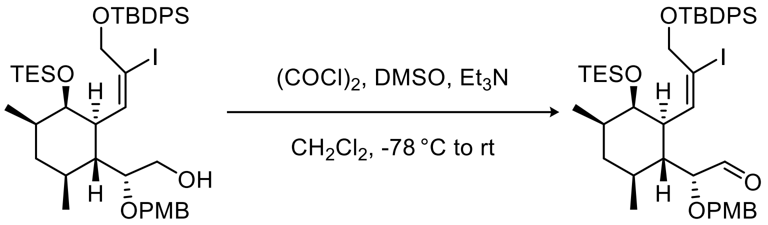

To a solution of oxalyl chloride (0.40 mL, 4.6 mmol) in CH_2Cl_2 (19 mL) at -78 °C was added dimethyl sulfoxide (0.48 mL, 6.9 mmol). After stirring for 5 min, to the mixture were added the solution of starting material (1.93 g, 2.3 mmol) in CH_2Cl_2 (4 mL) and triethylamine (1.92 mL, 13.8 mmol). The resulting mixture was stirred for 30 min at room temperature. The reaction mixture was quenched with saturated aqueous NH_4Cl at 0 °C. The mixture was extracted with CH_2Cl_2. The organic layer was washed with water and brine, dried over anhydrous Na_2SO_4, and concentrated *in vacuo*. The residue was purified by column chromatography (silica gel containing 5 % triethylamine, hexane/EtOAc = 3:1) to give the titled compound (1.75 g, 90%) as a colorless oil.

$[\alpha]_D^{22}$ = - 22.5 (c = 2.1, $CHCl_3$);

IR (ATR, $CHCl_3$) ν_{max} 3070, 3048, 2998, 2954, 2931, 2909, 2874, 2857, 1724, 1612, 1587, 1461, 1427, 1365, 1303, 1248, 1171, 1103, 1062, 1006, 821, 738, 700, 615, 503 cm^{-1};

^1H NMR (600 MHz, $CDCl_3$) δ : 9.62 (1H, s), 7.68-7.64 (4H, m), 7.45-7.35 (6H, m), 7.09 (2H, m), 6.75 (2H, m), 5.96 (1H, d, J = 11.4 Hz), 4.52 (1H, d, J = 11.4 Hz), 4.47 (1H, dd, J = 15.3, 0.6 Hz), 4.30 (1H, d, J = 11.4 Hz), 4.10 (1H, d, J = 13.2 Hz), 3.77 (1H, s), 3.76 (3H, s), 3.50 (1H, brs), 2.37 (1H, dt, J = 11.3, 1.8 Hz), 1.92-1.88 (1H, t, J = 10.8 Hz), 1.41-1.36 (1H, m), 1.28-1.27 (1H, m), 1.23-1.16 (2H, m), 1.06 (9H, s), 0.96 (9H, t, J = 7.8 Hz), 0.81 (3H, d, J = 6.6 Hz), 0.71 (3H, d, J = 6.6 Hz), 0.62-0.57 (6H, m);

^{13}C NMR (150 MHz, $CDCl_3$) δ : 206.7, 159.4, 146.1, 135.6, 133.5, 133.4, 129.7, 127.7, 127.6, 113.8, 105.1, 82.3, 75.1, 73.0, 66.4, 55.2, 47.1, 46.5, 36.8, 36.3, 32.5, 26.7, 20.2, 19.3, 18.6,

7.11, 5.43;

ESI-MS(positive) for calcd for $C_{43}H_{65}INO_5Si_2$ $[M+NH_4]^+$ 858.3441, found 858.3440.

Methyl (*S*,*Z*)-4-((1*R*,2*R*,3*R*,4*R*,6*S*)-2-((*E*)-3-((*tert*-butyldiphenylsilyl)oxy)-2-iodoprop-1-en-1-yl)-4,6-dimethyl-3-((triethylsilyl)oxy)cyclohexyl)-4-((4-methoxybenzyl)oxy)but-2-enoate (25)

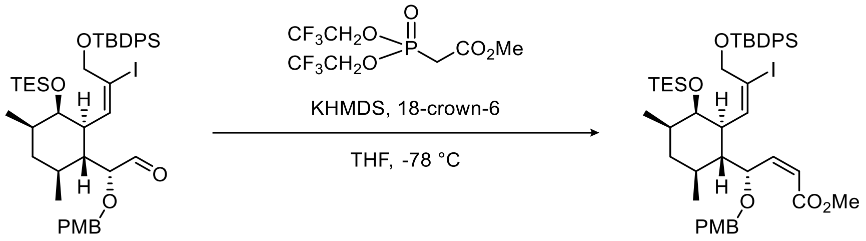

To a solution of bis(2,2,2-trifluoromethyl)(methoxy carbonylmethyl)phosphonate (0.55 g, 1.712 mmol) and 18-Crown-6 (1.52 g, 5.75 mmol) in THF (15 mL) at -78 ℃ was added a solution of potassium bis(trimethylsilyl)amide (0.6 M in toluene, 2.87 mL, 1.72 mmol). The reaction was stirred at -78 ℃ for 75 min, then was added a solution of the starting material (0.97 g, 1.15 mmol) in THF (8 mL). The mixture was stirred for 1 h at -60 ℃. The reaction mixture was quenched with saturated aqueous NH_4Cl at -78 ℃. The mixture was extracted with EtOAc. The organic layer was washed with water and brine, dried over anhydrous Na_2SO_4, and concentrated *in vacuo*. The residue was purified by column chromatography (silica gel, hexane/EtOAc = 5:1) to give the titled compound (0.95 g, 92% yield) as a colorless oil.

$[\alpha]_D^{24}$ = + 56.5 (c = 0.85, $CHCl_3$);

IR (ATR, neat) ν_{max} 3071, 2953, 2932, 2910, 2874, 1720, 1613, 1513, 1461, 1428, 1245, 1194, 1177, 1108, 1055, 1005, 822, 737, 700, 503 cm^{-1};

^{1}H NMR (600 MHz, $CDCl_3$) δ : 7.69-7.65 (4H, m), 7.42-7.40 (2H, m), 7.37-7.34 (4H, m), 7.05 (2H, m), 6.71 (2H, m), 6.36 (1H, dd, *J* = 12.0, 7.2 Hz), 6.14 (1H, d, *J* = 10.8 Hz), 5.84 (1H, dd, *J* = 12.0, 1.8 Hz), 5.10 (1H, d, *J* = 7.2 Hz), 4.43 (1H, d, *J* = 12.6 Hz), 4.34 (1H, d, *J* = 11.4 Hz), 4.11 (2H, t, *J* = 12.0 Hz), 3.75 (3H, s), 3.67 (3H, s), 3.53 (1H, s), 2.51 (1H, td, *J* = 10.8, 1.8 Hz), 1.79 (1H, t, *J* = 10.8 Hz), 1.55 (1H, m), 1.38-1.20 (3H, m), 1.06 (9H, s), 0.92 (9H, t, *J* = 7.8 Hz), 0.87 (3H, d, *J* = 6.6 Hz), 0.80 (3H, d, *J* = 6.6 Hz), 0.61-0.51 (6H, m);

^{13}C NMR (150 MHz, $CDCl_3$) δ : 166.1, 158.8, 155.7, 149.1, 135.5, 133.2, 133.1, 130.3, 129.5, 129.1, 127.5, 117.7, 113.4, 101.9, 76.6, 76.5, 71.0, 66.1, 55.0, 51.0, 48.3, 46.4, 36.8, 36.6, 32.9, 26.6, 19.7, 19.1, 18.5, 6.9, 5.3;

ESI-MS(positive) for calcd for $C_{46}H_{69}INO_6Si_2$ $[M+NH_4]^+$ 914.3703, found 914.3703.

Methyl (*S*,*Z*)-4-((1*R*,2*R*,3*R*,4*R*,6*S*)-3-hydroxy-2-((*E*)-3-hydroxy-2-iodoprop-1-en-1-yl)-4,6-dimethylcyclohexyl)-4-((4-methoxybenzyl)oxy)but-2-enoate (26)

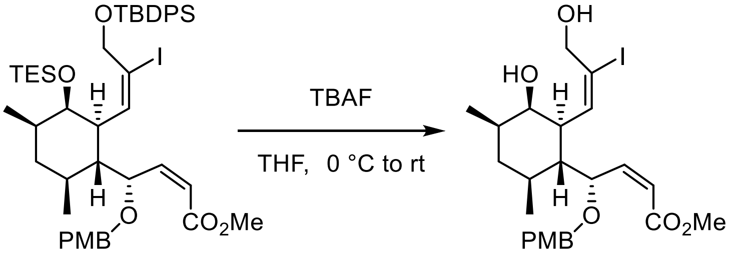

To a stirred solution of the starting material (0.45 g, 0.5 mmol) in anhydrous THF (10 mL) at 0 ℃ was added tetrabutylammonium fluoride (1.0 M in THF, 1.25 mL, 1.25 mmol). The resulting mixture was warmed to room temperature and stirred for 4 h. The reaction mixture was quenched with saturated aqueous NH_4Cl at 0 ℃. The mixture was

extracted with EtOAc. The organic layer was washed with water and brine, dried over anhydrous Na_2SO_4, and concentrated *in vacuo*. The residue was purified by column chromatography (silica gel, hexane/EtOAc = 4:1) to give the titled compound (0.25 g, 92% yield) as a colorless oil.

$[\alpha]_D^{23}$ = + 25.1 (c = 0.41, $CHCl_3$);

IR (ATR, neat) ν_{max} 3449, 2953, 2931, 2911, 2871, 1719, 1612, 1513, 1459, 1438, 1401, 1385, 1302, 1247, 1198, 1178, 1037, 990, 822, 755, 513 cm^{-1};

^1H NMR (600 MHz, $CDCl_3$) δ : 7.21 (2H, m), 6.87 (2H, m), 6.47 (1H, dd, J = 12, 7.2 Hz), 6.36 (1H, dd, J = 11.4, 0.6 Hz), 5.87 (1H, dd, J = 11.4, 1.6 Hz), 5.30 (1H, d, J = 7.2 Hz), 4.56 (1H, ddd, J = 13.2, 4.2, 0.6 Hz), 4.51 (1H, d, J = 11.4 Hz), 4.27 (1H, d, J = 10.8 Hz), 4.01 (1H, dd, J = 13.8, 10.2 Hz), 3.80 (3H, s), 3.70 (3H, s), 3.57 (1H, s), 3.17 (1H, dd, J = 9.6, 4.2 Hz), 2.76 (1H, td, J = 11.4, 2.4 Hz), 1.79 (1H, t, J = 10.8 Hz), 1.62 (2H, m), 1.33 (1H, m), 1.26 (1H, m), 1.21 (1H, m), 0.93 (3H, d, J = 6.6 Hz), 0.90 (3H, d, J = 6.6 Hz);

^{13}C NMR (150 MHz, $CDCl_3$) δ : 166.1, 159.7, 154.3, 148.3, 130.1, 129.2, 118.5, 113.8, 101.6, 75.2, 74.8, 72.2, 67.5, 55.2, 51.3, 48.4, 45.5, 36.6, 36.2, 33.4, 19.9, 17.8;

ESI-MS(positive) for calcd for $C_{24}H_{33}IKO_6[M+K]^+$ 583.0953, found 583.0952.

Methyl (*S,Z*)-4-((6a*R*,7*R*,8*S*,10*R*,10a*R*,*E*)-5-iodo-8,10-dimethyl-2,2-diphenyl-6a,7,8,9,10,10a-hexahydro-4*H*-benzo[d][1,3,2]dioxasilocin-7-yl)-4-((4-methoxybenzyl)oxy)but-2-enoate (8)

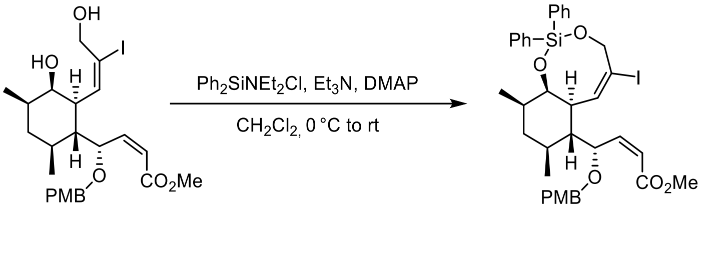

To a solution of 1-Chloro-*N,N*-diethyl-1,1-diphenylsilanamine (156 mg, 0.539 mmol) and triethylamine (0.1 mL, 0.69 mmol) in CH_2Cl_2 (8.5 mL) at 0 °C were added a solution of the starting material (270 mg, 0.49 mmol) in CH_2Cl_2 (14.8 mL) and 4-dimethylaminopyridine (9.0 mg, 0.07 mmol). The reaction was stirred at room temperature for 30 min, then added 4-dimethylaminopyridine (158 mg, 1.3 mmol). The resulting mixture was stirred at the same temperature overnight. The reaction mixture was quenched with saturated aqueous $NaHCO_3$ at 0 °C. The mixture was extracted with CH_2Cl_2. The organic layer was washed with water and brine, dried over anhydrous Na_2SO_4, and concentrated *in vacuo*. The residue was purified by column chromatography (silica gel, hexane/EtOAc = 20:1) to give the titled compound (324.5 mg, 90% yield) as a colorless oil.

$[\alpha]_D^{23}$ = + 143.4 (c = 0.81, $CHCl_3$);

IR (ATR, neat) ν_{max} 2954, 1719, 1613, 1513, 1458, 1430, 1248, 1198, 1179, 1125, 1116, 1069, 1022, 823, 781, 741, 717, 700, 579, 515, 406 cm^{-1};

^1H NMR (600 MHz, $CDCl_3$) δ : 7.72 (4H, m), 7.39 (2H, m), 7.37 (4H, m), 7.20 (2H, m), 6.87 (2H, m), 6.65 (1H, d, J = 10.2 Hz), 6.49 (1H, dd, J = 12.0, 7.8 Hz), 5.80 (1H, dd, J = 12.0, 1.2 Hz), 5.23 (1H, d, J = 7.8 Hz), 4.84 (1H, d, J = 12.6 Hz), 4.51 (1H, d, J = 12.0 Hz), 4.24 (1H, d, J = 12.0 Hz), 4.10 (1H, d, J = 12.6 Hz), 3.80 (3H, s), 3.70 (3H, s), 3.69 (1H, s), 2.43 (1H, t, J = 11.4 Hz), 2,23 (1H, t, J = 10.8 Hz), 1.68-1.36 (4H, m), 1.00 (3H, d, J = 6.6 Hz), 0.89 (3H, d, J = 6.6 Hz);

^{13}C NMR (150 MHz, $CDCl_3$) δ : 166.1, 159.2, 155.0, 146.8, 135.7, 134.5, 134.0, 131.7, 130.5, 130.4, 130.2, 129.4, 127.8, 127.7, 117.8, 113.7, 105.3, 76.1, 74.3, 71.4, 71.2, 55.3, 51.4, 48.1, 47.5, 37.3, 37.3, 34.1, 20.3, 18.8;

ESI-MS(positive) for calcd for $C_{36}H_{45}INO_6Si[M+NH_4]^+$ 742.2055, found 742.2055.

2,5-Dimethyl-3-(tributylstannyl)cyclopent-2-en-1-one (9)

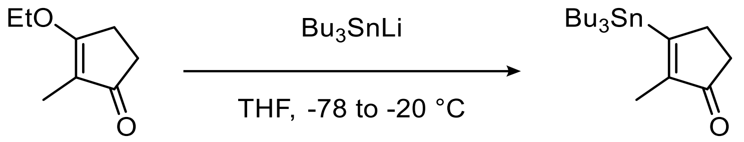

A solution of hexabutylditin (3.66 mL, 7.3 mmol) in THF (52 mL) at -78 ℃ was treated with *n*-Butyl lithium (1.45 M solution in hexanes, 4.8 mL, 7.0 mmol). The reaction mixture was stirred at -60 ℃ for 1 h and then cooled to -78 ℃. The mixture was treated with a solution of the starting material (818 mg, 5.84 mmol) in THF, and the reaction mixture was stirred at -20 ℃ for 1 h. The reaction mixture was quenched with saturated aqueous NH_4Cl at 0 ℃. The mixture was extracted with EtOAc. The organic layer was washed with water and brine, dried over anhydrous Na_2SO_4, and concentrated *in vacuo*. The residue was purified by column chromatography (silica gel, hexane/EtOAc = 40:1) to give the titled compound (1.75 g, 78% yield) as a yellow oil.

IR (ATR, neat) ν_{max} 2955, 2921, 2871, 2853, 1698, 1461, 1376, 1282, 1075, 959, 872, 799, 667, 597, 508 cm^{-1};

^1H NMR (600 MHz, $CDCl_3$) δ : 2.69-2.67 (2H, m), 2.35-2.33 (2H, m), 1.82 (3H, m), 1.55-1.48 (6H, m), 1.35-1.29 (6H, m), 1.11-1.01 (6H, m), 0.90 (9H, t, *J* = 7.2 Hz);

^{13}C NMR (150 MHz, $CDCl_3$) δ : 209.3, 181.3, 150.1, 35.1, 33.8, 29.1, 27.3, 13.6, 12.2, 9.5;

ESI-MS(positive) for calcd for $C_{18}H_{35}O^{120}Sn[M+H]^+$ 387.1704, found 387.1706.

Methyl (*S,Z*)-4-((6a*S*,7*R*,8*S*,10*R*,10a*R,Z*)-8,10-dimethyl-5-(2-methyl-3-oxocyclopent-1-en-1-yl)-2,2-diphenyl-6a,7,8,9,10,10a-hexahydro-4*H*-benzo[d][1,3,2]dioxasilocin-7-yl)-4-((4-methoxybenzyl)oxy)but-2-enoate (7)

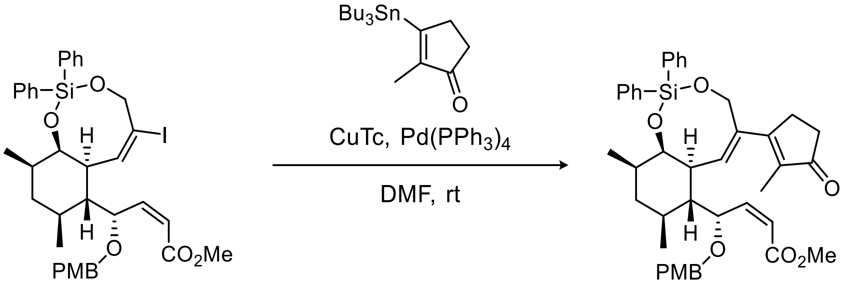

To a stirred suspension of copper (I) 2-thiophenecarboxylate (33 mg, 0.17 mmol) and tetrakis(triphenyl)phosphine palladium (24 mg, 0.02 mmol) in DMF (0.5 mL) at room temperature were added a solution of vinyl iodide (50 mg, 0.069 mmol) and vinyl stannane (66 mg, 0.17 mmol) in DMF (0.5 mL). The resulting mixture was stirred at room temperature overnight. The reaction mixture was diluted with Et_2O (5 mL) and quenched with 5% aqueous NH_4OH (6 mL) and brine (1.2 mL) at 0 ℃. The mixture was extracted with hexane/EtOAc = 3:1. The organic layer was washed with water and brine, dried over anhydrous Na_2SO_4, and concentrated *in vacuo*. The residue was purified by column chromatography (silica gel, hexane/EtOAc = 5:1) to give the titled compound (44.5 mg, 93% yield) as a colorless oil.

$[\alpha]_D{}^{23}$ = + 40.1 (c = 0.48, $CHCl_3$);

IR (ATR, $CHCl_3$) ν_{max} 2954, 2919, 2872, 1720, 1694, 1612, 1589, 1513, 1430, 1382, 1327, 1302, 1247, 1198, 1179, 1124, 1116, 1063, 1040, 998, 825, 743, 717, 701, 583, 515, 402 cm^{-1};

^1H NMR (600 MHz, $CDCl_3$) δ : 7.69-7.68 (2H, m), 7.42-7.35 (6H, m), 7.25-7.23 (2H, m), 7.19-7.17 (2H, m), 6.85-6.83 (2H, m), 6.36 (1H, d, J = 10.2 Hz), 6.31 (1H, dd, J = 11.4, 7.8 Hz), 5.65-5.63 (1H, dd, J = 11.4, 1.8 Hz), 5.26-5.23 (1H, d, J = 7.8 Hz), 4.64 (1H, d, J = 11.4 Hz), 4.48 (1H, d, J = 11.4 Hz), 4.23 (2H, t, J = 10.8 Hz), 3.81 (1H, brs), 3.79 (3H, s), 3.68 (3H, s), 2.82-2.78 (1H, m), 2.73-2.71 (1H, m), 2.59-2.53 (1H, m), 2.49-2.43 (2H, m),

2.37 (1H, m), 1.90 (3H, t, J = 1.8 Hz), 1.68-1.58 (2H, m), 1.42-1.33 (2H, m), 1.04 (3H, d, J = 6.0 Hz), 0.94 (3H, d, J = 6.0 Hz);

^{13}C NMR (150 MHz, $CDCl_3$) δ : 210.2, 167.6, 165.9, 159.2, 153.7, 141.3, 139.1, 136.6, 135.0, 134.4, 134.2, 132.0, 130.4, 130.3, 130.2, 129.4, 127.8, 127.6, 118.8, 113.6, 76.2, 74.7, 71.2, 60.1, 55.2, 51.3, 47.7, 45.5, 37.8, 37.5, 34.3, 33.7, 28.3, 20.5, 18.9, 10.2;

ESI-MS(positive) for calcd for $C_{42}H_{48}KO_7Si[M+K]^+$ 731.2801, found 731.2802.

Methyl (1*R*,3*S*,3a*R*,3a¹*S*,4*R*,4a*S*,4a¹*S*,5*S*,5a*S*,12a*R*)-4-((4-methoxybenzyl)oxy)-1,3,5a-trimethyl-6-oxo-11,11-diphenyl-2,3,3a,3a1,4,4a,4a¹,5,5a,6,7,8,9,12a-tetradecahydro-1*H*-cyclopenta[6,7]fluoreno[4,5-def][1,3,2]dioxasilocine-5-carboxylate (6)

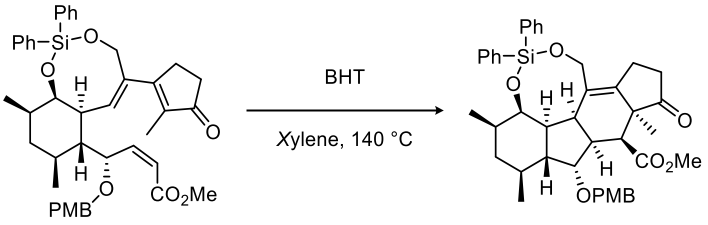

A solution of cyclization precursor (170 mg, 0.24 mmol) and BHT (27 mg, 0.12 mmol) in xylene (122 mL) was stirred at 140°C in an oil bath for 24 h. The reaction mixture was cooled to room temperature. Then, the organic solvent was removed by evaporation. The residue was purified by column chromatography (silica gel, hexane/EtOAc = 8:1) to give the IMDA adduct (128.6 mg, 76% yield) as a colorless oil.

$[\alpha]_D{}^{23}$ = + 40.2 (c = 0.66, $CHCl_3$);

IR (ATR, $CHCl_3$) ν_{max} 2952, 2926, 1740, 1611, 1588, 1513, 1455, 1430, 1370, 1301, 1247, 1195, 1175, 1125, 1113, 1075, 1040, 910, 826, 787, 764, 740, 715, 700, 581, 510, 412 cm-

1;

^{1}H NMR (600 MHz, $CDCl_3$) δ : 7.70-7.69 (2H, m), 7.55-7.54 (2H, m), 7.37-7.26 (8H, m), 6.88 (2H, m), 4.84 (1H, d, *J* = 12.0 Hz), 4.68 (1H, s), 4.56-4.49 (3H, m), 4.24 (1H, d, *J* = 3.0 Hz), 3.80 (3H, s), 3.21 (3H, s), 3.08-3.05 (1H, m), 3.00-2.97 (1H, m), 2.89 (1H, d, *J* = 7.8 Hz), 2.67 (1H, t, *J* = 9.0 Hz), 2.53-2.45 (1H, m), 2.42-2.36 (1H, m), 2.30-2.24 (1H, m), 1.83 (1H, dt, *J* = 19.2, 10.2 Hz), 1.65 (1H, m), 1.43 (1H, m), 1.21-1.19 (1H, m), 1.16-1.04 (2H, m), 0.93 (3H, s), 0.80 (3H, d, *J* = 6.0 Hz), 0.22 (3H, d, *J* = 6.0 Hz);

^{13}C NMR (150 MHz, $CDCl_3$) δ : 221.5, 171.7, 159.2, 144.1, 136.3, 135.2, 134.7, 133.5, 131.1, 129.8, 129.3, 127.6, 127.4, 127.2, 113.8, 80.6, 71.7, 71.3, 64.8, 55.3, 51.1, 50.2, 49.2, 47.5, 47.1, 41.4, 39.9, 38.5, 37.1, 36.4, 31.0, 23.3, 22.1, 19.9, 18.9;

ESI-MS(positive) for calcd for $C_{42}H_{48}KO_7Si[M+K]^+$ 731.2801, found 731.2795.

Methyl (1*R*,3*S*,3a*R*,3a^1*S*,4*R*,4a*S*,4a^1*S*,5*S*,5a*S*,12a*R*)-4-((4-methoxybenzyl)oxy)-1,3,5a-trimethyl-6-oxo-11,11-diphenyl-2,3,3a,3a1,4,4a,4a^1,5,5a,6,9,12a-dodecahydro-1*H*-cyclopenta[6,7]fluoreno[4,5-def][1,3,2]dioxasilocine-5-carboxylate (27)

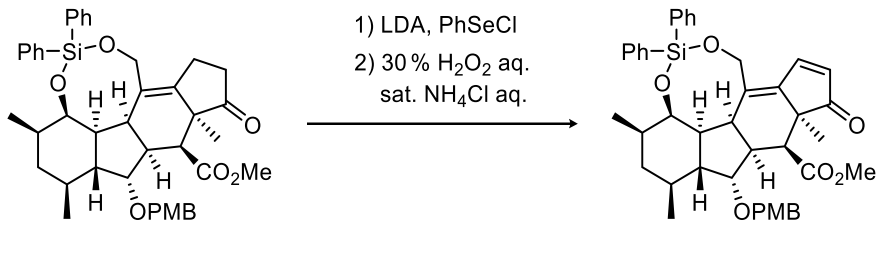

To a stirred solution of diisopropylethylamine (0.031 mL, 0.223 mmol) in THF (2 mL) at 0 ℃ was added a solution of *n*-butyl lithium (1.42 M in hexane, 0.144 mL, 0.204 mmol) dropwise. After stirring for 30 min, a solution of the starting material (128.6 mg, 0.186

mmol) in THF (1.0 mL) was added dropwise at -78 °C. The resulting mixture was stirred at the same temperature for 30 min, then added a solution of phenylselenenyl chloride (53.3 mg, 0.278 mmol) in THF (0.7 mL) dropwise and stirred for 30 min. The reaction mixture was quenched with saturated aqueous NH_4Cl at -78 °C. The mixture was extracted with EtOAc. The organic layer was washed with water and brine, dried over anhydrous Na_2SO_4, and concentrated *in vacuo*. The residue was used in the next step without further purification.

To a stirred solution of the crude residue in CH_2Cl_2 (4.8 mL) at 0 °C was added saturated aqueous NH_4Cl (0.04 mL) and 30% aqueous hydrogen peroxide (0.805 mL, 0.644 mmol) dropwise. The resulting mixture was stirred at the same temperature for 30 min. The reaction mixture was quenched with saturated aqueous $Na_2S_2O_3$ at 0 °C. The mixture was extracted with CH_2Cl_2. The organic layer was washed with water and brine, dried over anhydrous Na_2SO_4, and concentrated *in vacuo*. The residue was purified by column chromatography (silica gel, hexane/EtOAc = 3:1) to give the desired enone (97.4 mg, 76% yield for 2 steps) as a colorless oil.

$[\alpha]_D^{23}$ = + 125.4 (*c* = 0.69, $CHCl_3$);

IR (ATR, $CHCl_3$) ν_{max} 3417, 3070, 3010, 2950, 2927, 2870, 1705, 1611, 1588, 1537, 1512, 1456, 1429, 1374, 1301, 1246, 1198, 1173, 1126, 1112, 1069, 1029, 998, 823, 789, 751, 716, 700, 584, 515, 491cm^{-1};

^1H NMR (600 MHz, $CDCl_3$) δ : 8.08 (1H, d, *J* = 6.0 Hz), 7.64 (2H, d, *J* = 6.6 Hz), 7.59 (2H, d, *J* = 6.6 Hz), 7.39-7.28 (6H, m), 7.30-7.27 (2H, d, *J* = 9.0 Hz), 6.89 (2H, d, *J* = 9.0 Hz), 5.97 (1H, dd, *J* = 6.0, 1.2 Hz), 5.01 (1H, dd, *J* = 12.0, 1.2 Hz), 4.75 (1H, d, *J* = 12.0 Hz), 4.72 (1H, s), 4.53 (1H, d, *J* = 5.4 Hz), 4.49 (1H, d, *J* = 5.4 Hz), 4.25 (1H, d, *J* = 3.6 Hz),

3.80 (3H, s), 3.25 (1H, t, J = 9.6 Hz), 2.95 (1H, d, J = 7.8 Hz), 2.91 (3H, s), 2.78 (1H, t, J = 9.0 Hz), 2.50 (1H, td, J = 10.8, 1.8 Hz), 1.71-1.59 (1H, m), 1.41-1.48 (1H, m), 1.21 (1H, dt, J = 13.2, 3.6 Hz), 1.14-1.09 (1H, m), 1.08 (3H, s), 1.07-1.05 (1H, m), 0.81 (3H, d, J = 6.6 Hz), 0.30 (3H, d, J = 6.6 Hz);

^{13}C NMR (150 MHz, $CDCl_3$) δ : 210.2, 171.2, 159.2, 145.2, 135.3, 135.2, 132.8, 132.6, 131.0, 130.1, 130.0, 129.32, 129.30, 127.6, 127.4, 113.8, 80.5, 72.3, 71.4, 63.0, 55.3, 50.9, 50.4, 47.7, 47.6, 46.3, 42.5, 40.9, 38.7, 36.7, 31.6, 36.4, 31.0, 23.9, 19.2;

ESI-MS(positive) for calcd for $C_{42}H_{46}NaO_7Si$[M+Na]$^+$ 713.2905, found 713.2896.

Methyl (1*R*,3*S*,3a*R*,3a1*S*,4*R*,4a*S*,4a1*S*,5*S*,5a*S*,8*R*,12a*R*)-4-((4-methoxybenzyl)oxy)-1,3,5a,8-tetramethyl-6-oxo-11,11-diphenyl-2,3,3a,3a1,4,4a,4a1,5,5a,6,7,8,9,12a-tetradecahydro-1*H*-cyclopenta[6,7]fluoreno[4,5-def][1,3,2]dioxasilocine-5-carboxylate (28)

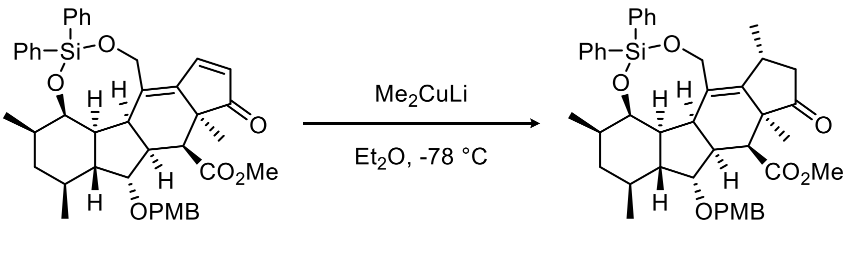

To a suspension of CuI (7.4 mg, 0.37 mmol) in Et_2O (1.7 mL) at 0 ℃ was added MeLi (1.16 M in Et_2O, 0.63 mL, 0.74 mmol), and the reaction mixture turned a colorless. After stirring for 15 min at 0 ℃, the mixture was cooled to -78 ℃. The reaction mixture was added a solution of enone (127.7 mg, 0.185 mmol) in Et_2O (2 mL) dropwise and stirred at the same temperature for 30 min. The reaction mixture was quenched with saturated aqueous NH_4Cl at -78 ℃. The mixture was extracted with EtOAc. The organic layer was

washed with water and brine, dried over anhydrous Na_2SO_4, and concentrated *in vacuo*. The residue was purified by column chromatography (silica gel, hexane/EtOAc = 8:1) to give the titled compound (117.7 mg, 90% yield) as a yellow oil.

$[\alpha]_D^{23}$ = + 95.8 (c = 1.37, $CHCl_3$);

IR (ATR, $CHCl_3$) ν_{max} 3005, 2950, 2928, 2910, 2870, 1737, 1611, 1512, 1457, 1429, 1371, 1301, 1246, 1196, 1171, 1112, 1085, 1063, 1034, 997, 825, 753, 714, 700, 580, 510, 492 cm^{-1};

^1H NMR (600 MHz, $CDCl_3$) δ : 7.69-7.68 (2H, m), 7.55-7.54 (2H, m), 7.37-7.26 (8H, m), 6.88-6.87 (2H, m), 5.02 (1H, d, J = 12.0 Hz), 4.71 (1H, s), 4.61 (1H, d, J = 12.0 Hz), 4.55-4.51 (2H, m), 4.24 (1H, d, J = 3.6 Hz), 3.80 (3H, s), 3.67-3.62 (1H, m), 3.21 (3H, s), 3.05-3.02 (1H, m), 2.82 (1H, d, J = 7.8 Hz), 2.69 (1H, t, J = 8.4 Hz), 2.41 (1H, t, J = 13.2 Hz), 2.25 (1H, dd, J = 19.2, 9.6 Hz), 1.98 (1H, dd, J = 19.2, 1.8 Hz), 1.66 (1H, m), 1.43 (1H, m), 1.28 (3H, d, J = 4.8 Hz), 1.22-1.06 (3H, m), 1.05 (3H, s), 0.82 (3H, d, J = 6.6 Hz), 0.24 (3H, d, J = 6.6 Hz);

^{13}C NMR (150 MHz, $CDCl_3$) δ : 222.3, 171.5, 159.2, 148.0, 136.2, 135.1, 134.6, 133.4, 131.1, 129.8, 129.3, 128.8, 127.4, 127.1, 113.8, 80.7, 71.9, 71.4, 63.6, 55.2, 51.0, 49.7, 49.6, 49.0, 47.2, 45.6, 41.5, 39.9, 38.5, 37.0, 31.0, 30.3, 24.3, 21.4, 19.9, 19.0;

ESI-MS(positive) for calcd for $C_{43}H_{54}NO_7Si[M+NH_4]^+$ 724.3664, found 724.3664.

Methyl (1*R*,3*S*,3a*R*,3a^1*S*,4*R*,4a*S*,4a^1*S*,5*S*,5a*S*,7*R*,8*R*,12a*R*)-4-((4-methoxybenzyl)oxy)-1,3,5a,7,8-pentamethyl-6-oxo-11,11-diphenyl-2,3,3a,3a1,4,4a,4a1,5,5a,6,7,8,9,12a-tetradecahydro-1*H*-cyclopenta[6,7]fluoreno[4,5-def][1,3,2]dioxasilocine-5-carboxylate (29)

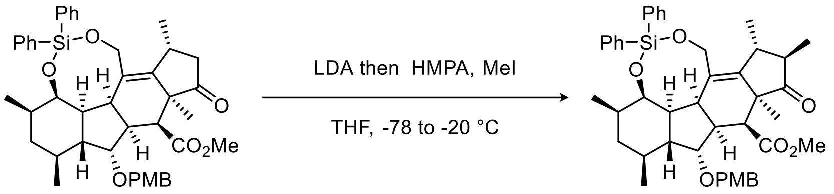

To a stirred solution of diisopropylethylamine (0.04 mL, 0.25 mmol) in THF (1.5 mL) at 0 ℃ was added a solution of *n*-butyl lithium (1.46 M in hexane, 0.170 mL, 0.24 mmol) dropwise. After stirring for 30 min, a solution of the starting material (57.3 mg, 0.08 mmol) in THF (1.1 mL) was added dropwise at -78 ℃. The resulting mixture was stirred at the same temperature for 30 min, then added hexamethylphosphoric triamide (0.65 mL) and MeI (0.01 mL, 0.16 mmol) dropwise. The resulting mixture was warmed to -20 ℃ and stirred for 1 h. The reaction mixture was quenched with saturated aqueous NH_4Cl at -78 ℃. The mixture was extracted with EtOAc. The organic layer was washed with water and brine, dried over anhydrous Na_2SO_4, and concentrated *in vacuo*. The residue was purified by column chromatography (silica gel, hexane/EtOAc = 8:1) to give the titled compound (145.2 mg, 91% yield) as a colorless oil.

$[\alpha]_D^{23}$ = + 77.7 (*c* = 1.23, $CHCl_3$);

IR (ATR, $CHCl_3$) ν_{max} 2952, 2929, 2870, 1738, 1612, 1513, 1456, 1430, 1372, 1301, 1246, 1195, 1172, 1126, 1112, 1084, 1066, 1036, 998, 822, 755, 715, 700, 514, 491 cm^{-1};

^1H NMR (600 MHz, $CDCl_3$) δ : 7.70-7.69 (2H, m), 7.54-7.53 (2H, m), 7.37-7.26 (8H, m), 6.88-6.87 (2H, m), 5.06 (1H, d, *J* = 12.6 Hz), 4.71 (1H, s), 4.64 (1H, d, *J* = 12.6 Hz), 4.53-4.47 (2H, m), 4.21 (1H, d, *J* = 3.6 Hz), 3.79 (3H, s), 3.15-3.13 (1H, m), 3.06 (3H, s), 3.06-3.02 (1H, t, *J* = 10.8 Hz), 2.83 (1H, d, *J* = 7.8 Hz), 2.67 (1H, dd, *J* = 9.0, 7.8 Hz), 2.39 (1H, m), 2.19-2.13 (1H, m), 1.66 (1H, m), 1.43 (1H, m), 1.42 (3H, d, *J* = 7.2 Hz), 1.22-1.19 (1H, m), 1.16-1.04 (2H, m), 1.07 (3H, s), 0.82 (3H, d, *J* = 6.0 Hz), 0.76 (3H, d, *J* = 7.2 Hz), 0.25

(3H, d, J = 7.2 Hz);

^{13}C NMR (150 MHz, $CDCl_3$) δ : 223.1, 171.8, 159.1, 145.7, 136.2, 135.2, 134.8, 133.2, 131.1, 129.9, 129.8, 129.3, 129.2, 127.4, 127.2, 113.8, 80.7, 72.1, 71.3, 63.1, 55.2, 52.0, 51.0, 49.7, 49.6, 48.2, 47.2, 41.3, 40.2, 39.0, 38.7, 37.0, 31.0, 24.6, 20.8, 19.9, 19.0, 15.2;

ESI-MS(positive) for calcd for $C_{44}H_{53}O_7Si[M+H]^+$ 721.3555, found 721.3553.

Methyl (2*R*,3*R*,4a*R*,4b*S*,5*R*,6*R*,8*S*,8a*R*,9*R*,9a*S*,10*S*,10a*S*)-5-hydroxy-4-(hydroxymethyl)-9-((4-methoxybenzyl)oxy)-2,3,6,8,10a-pentamethyl-1-oxo-1,2,3,4a,4b,5,6,7,8,8a,9,9a,10,10a-tetradecahydrocyclopenta[*b*]fluorene-10-carboxylate (30)

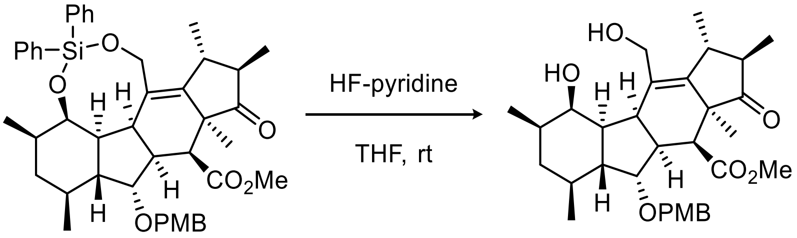

To a stirred solution of the starting material (180.8 mg, 0.25 mmol) in anhydrous THF (25 mL) at 0 ℃ was added hydrogen fluoride pyridine complex (0.73 mL). The resulting mixture was warmed to room temperature and stirred for 1 h. The reaction mixture was quenched with saturated aqueous $NaHCO_3$ at 0 ℃. The mixture was extracted with EtOAc. The organic layer was washed with water and brine, dried over anhydrous Na_2SO_4, and concentrated *in vacuo*. The residue was purified by column chromatography (silica gel, hexane/EtOAc = 1:1) to give the titled compound (122.0 mg, 90% yield) as a colorless oil.

$[\alpha]_D^{24}$ = + 18.7 (c = 0.93, $CHCl_3$);

IR (ATR, $CHCl_3$) ν_{max} 3462, 2951, 2929, 2908, 2870, 1736, 1716, 1611, 1512, 1455, 1373,

1302, 1246, 1202, 1173, 1069, 1032, 989, 821, 752, 666, 577 cm^{-1};

^{1}H NMR (600 MHz, CDCl$_3$) δ : 7.26 (2H, m), 6.89-6.87 (2H, m), 4.50 (3H, m), 4.19 (1H, s), 4.13 (1H, d, *J* = 12.0 Hz), 3.98 (1H, d, *J* = 3.6 Hz), 3.80 (3H, s), 3.60 (3H, s), 3.23-3.22 (1H, m), 3.13-3.02 (1H, m), 2.81 (1H, d, *J* = 6.6 Hz), 2.62 (1H, dd, *J* = 8.4, 7.2 Hz), 2.47 (1H, m), 2.36 (1H, brs), 2.31-2.28 (1H, m), 1.69-1.61 (1H, m), 1.53-1.50 (1H, m), 1.42 (1H, m), 1.42 (3H, d, *J* = 7.2 Hz), 1.15 (3H, s), 1.09 (3H, d, *J* = 8.4 Hz), 0.95 (3H, d, *J* = 6.6 Hz), 0.81 (3H, d, *J* = 6.6 Hz) 0.88-0.86 (2H, m);

^{13}C NMR (150 MHz, CDCl$_3$) δ : 222.6, 176.4, 159.3, 143.1, 130.7, 130.4, 129.3, 113.9, 81.1, 71.5, 71.3, 61.7, 55.3, 53.2, 52.1, 49.1, 48.9, 47.6, 47.3, 41.5, 39.2, 38.3, 38.1, 38.0, 31.7, 25.7, 21.0, 19.8, 18.0, 15.5;

ESI-MS(positive) for calcd for $C_{32}H_{45}O_7[M+H]^+$ 541.3160, found 541.3160.

Methyl (2*R*,3*R*,4a*R*,4b*S*,5*R*,6*R*,8*S*,8a*R*,9*R*,9a*S*,10*S*,10a*S*)-5-hydroxy-9-((4-methoxybenzyl)oxy)-2,3,6,8,10a-pentamethyl-4-((((methylthio)carbonothioyl)oxy)methyl)-1-oxo-1,2,3,4a,4b,5,6,7,8,8a,9,9a,10,10a-tetradecahydrocyclopenta[*b*]fluorene-10-carboxylate (31)

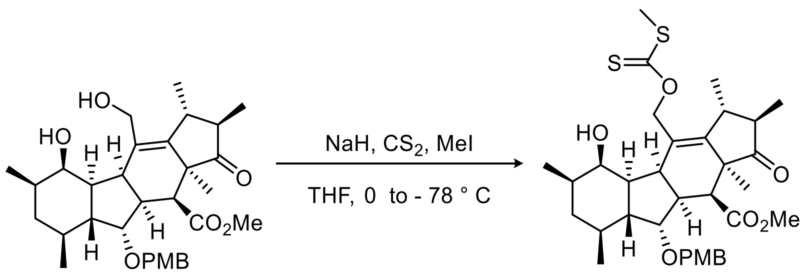

To a stirred solution of the starting material (27 mg, 0.05 mmol) in anhydrous THF (3.3 mL) and carbon disulfide (0.01 mL, 0.2 mmol) at 0 ℃ was added a suspension of sodium hydride (60% in oil, 0.2 mmol). After stirring for 1 h at the same temperature, the

resulting mixture was cooled to -78 ℃ and added MeI (0.025 mL, 0.04 mmol) dropwise. The resulting mixture was stirred for 30 min at the same temperature. The reaction mixture was quenched with saturated aqueous NH_4Cl at -78 ℃. The mixture was extracted with EtOAc. The organic layer was washed with water and brine, dried over anhydrous Na_2SO_4, and concentrated *in vacuo*. The residue was purified by column chromatography (silica gel, hexane/EtOAc = 8:1) to give the titled compound (25.8 mg, 84% yield) as a colorless oil.

$[\alpha]_D^{24}$ = + 45.0 (*c* = 1.01, $CHCl_3$);

IR (ATR, $CHCl_3$) ν_{max} 3549, 2959, 2926, 2870, 1738, 1717, 1611, 1512, 1455, 1373, 1301, 1247, 1205, 1173, 1064, 1033, 992, 803, 752, 666, 580 cm^{-1};

^1H NMR (600 MHz, $CDCl_3$) δ : 7.28 (2H, m), 6.90-6.89 (2H, m), 5.48 (2H, m), 4.50 (2H, m), 4.02 (2H, m), 3.80 (3H, s), 3.59 (3H, s), 3.28 (1H, m), 2.85 (1H, d, *J* = 7.2 Hz), 2.79-2.75 (1H, m), 2.66 (1H, dd, *J* = 8.4, 7.2 Hz), 2.58 (3H, s), 2.38-2.30 (2H, m), 1.79 (1H, brs), 1.66-1.60 (1H, m), 1.49-1.44 (1H, m), 1.39 (3H, d, *J* = 7.2 Hz), 1.38 (1H, m), 1.17 (3H, s), 1.10 (3H, d, *J* = 7.2 Hz), 0.98-0.84 (2H, m), 0.92 (3H, d, *J* = 6.6 Hz), 0.82 (3H, d, *J* = 6.6 Hz);

^{13}C NMR (150 MHz, $CDCl_3$) δ : 221.8, 215.5, 175.4, 159.3, 145.2, 130.7, 129.3, 125.8, 113.9, 81.4, 72.4, 71.6, 71.3, 55.3, 53.3, 52.2, 49.7, 49.2, 47.5, 47.2, 41.5, 40.5, 38.2, 37.9, 36.8, 31.7, 25.5, 22.4, 19.8, 19.2, 18.2, 15.7;

ESI-MS(positive) for calcd for $C_{34}H_{46}KO_7S_2[M+K]^+$ 669.2317, found 669.2312.

Methyl (2*R*,3*R*,4a*R*,4b*S*,5*R*,6*R*,8*S*,8a*R*,9*R*,9a*S*,10*S*,10a*S*)-5-hydroxy-9-((4-methoxybenzyl)oxy)-2,3,4,6,8,10a-hexamethyl-1-oxo-1,2,3,4a,4b,5,6,7,8,8a,9,9a,10,10a-tetradecahydrocyclopenta[*b*]fluorene-10-carboxylate (32)

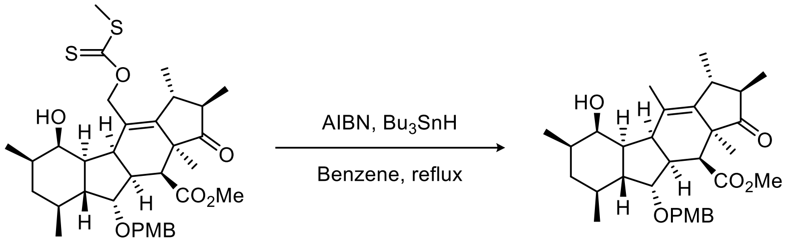

To a stirred solution of the starting material (5.0 mg, 0.0079 mmol) in Benzene (0.79 mL) were added Bu_3SnH (0.007 mL, 0.023 mmol) and AIBN (0.13 mg, 0.0007 mmol). The reaction mixture was stirred at 100 ℃ in an oil bath for 2 h. The reaction mixture was cooled to room temperature. Then, the organic solvent was removed by evaporation. The residue was purified by column chromatography (silica gel, hexane/EtOAc = 20:1) to give the titled compound (3.8 mg, 91% yield) as a colorless oil.

$[\alpha]_D{}^{24}$ = + 45.6 (c = 0.76, $CHCl_3$);

IR (ATR, $CHCl_3$) ν_{max} 3554, 2954, 2928, 2905, 2869, 1738, 1720, 1611, 1586, 1512, 1455, 1372, 1301, 1247, 1201, 1171, 1070, 1029, 951, 915, 803, 752, 701, 666, 578, 516, 409 cm^{-1};

^1H NMR (600 MHz, $CDCl_3$) δ : 7.29-7.26 (2H, m), 6.90-6.86 (2H, m), 4.51 (1H, d, J = 11.4 Hz), 4.48 (1H, d, J = 11.4 Hz), 4.19 (1H, s), 4.00 (1H, d, J = 4.2 Hz), 3.80 (3H, s), 3.57 (3H, s), 3.04 (1H, dd, J = 10.2 Hz), 2.80 (1H, d, J = 6.6 Hz), 2.70-2.64 (1H, m), 2.62 (1H, dd, J = 8.4, 7.2 Hz), 2.35 (1H, dd, J = 12.6 Hz), 2.26 (1H, qd, J = 7.8, 4.2 Hz), 1.97 (3H, s), 1.80 (1H, s), 1.66-1.58 (1H, m), 1.50-1.44 (1H, m), 1.37-1.35 (1H, m) 1.34 (3H, d, J = 7.2 Hz), 1.12 (3H, s), 1.10 (3H, d, J = 7.2 Hz), 0.98-0.92 (1H, m), 0.94 (3H, d, J = 7.2 Hz), 0.90-0.86 (1H, m), 0.82 (2H, d, J = 7.2 Hz);

^{13}C NMR (150 MHz, $CDCl_3$) δ : 223.1, 175.2, 159.2, 138.1, 130.9, 129.3, 126.9, 113.8, 81.8,

71.5, 71.2, 55.3, 52.7, 52.0, 49.8, 49.2, 47.8, 46.8, 41.5, 40.8, 40.7, 38.1, 38.0, 31.8, 25.7, 21.3, 19.8, 18.3, 17.4, 15.9;

ESI-MS(positive) for calcd for $C_{32}H_{45}O_6[M+H]^+$ 525.3211, found 525.3211.

Methyl (2*R*,3*R*,4a*R*,4b*S*,5*R*,6*R*,8*S*,8a*R*,9*R*,9a*S*,10*S*,10a*S*)-9-((4-methoxybenzyl)oxy)-2,3,4,6,8,10a-hexamethyl-5-(((methylthio)carbonothioyl)oxy)-1-oxo-1,2,3,4a,4b,5,6,7,8,8a,9,9a,10,10a-tetradecahydrocyclopenta[*b*]fluorene-10-carboxylate (33)

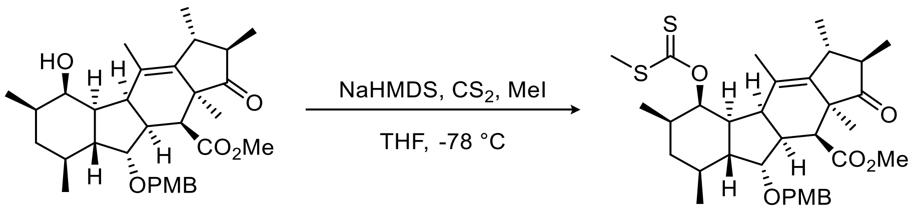

To a stirred solution of the starting material (8.3 mg, 0.016 mmol) in anhydrous THF (1.6 mL) and carbon disulfide (0.005 mL, 0.063 mmol) at -78 ℃ was added a solution of sodium bis(trimethylsilyl)amide (1.9 M in THF, 0.018mL, 0.035mmol). After stirring for 1 h, the resulting mixture was added MeI (0.008 mL, 0.128 mmol) dropwise and stirred for 30 min at the same temperature. The reaction mixture was quenched with saturated aqueous NH_4Cl at -78 ℃. The mixture was extracted with EtOAc. The organic layer was washed with water and brine, dried over anhydrous Na_2SO_4, and concentrated *in vacuo*. The residue was purified by column chromatography (silica gel, hexane/EtOAc = 11:1) to give the titled compound (9.1 mg, 94% yield) as a colorless oil.

$[\alpha]_D^{24}$ = + 37.3 (c = 0.61, $CHCl_3$);

IR (ATR, $CHCl_3$) ν_{max} 2957, 2928, 2268, 1737, 1612, 1585, 1512, 1456, 1372, 1301, 1247, 1301, 1247, 1228, 1210, 1170, 1097, 1069, 1045, 805, 754, 665, 577, 515 cm^{-1};

^{1}H NMR (600 MHz, $CDCl_3$) δ : 7.30-7.27 (2H, m), 6.91-6.87 (2H, m), 6.50 (1H, s), 4.54 (1H, d, *J* = 5.4 Hz), 4.48 (1H, d, *J* = 5.4 Hz), 4.16 (1H, d, *J* = 3.6 Hz), 3.80 (3H, s), 3.61 (3H, s), 3.04 (1H, dd, *J* = 9.6, 10.2 Hz), 2.80 (1H, d, *J* = 7.2 Hz), 2.66 (1H, dd, *J* = 7.2 Hz), 2.61-2.56 (1H, m), 2.53 (3H, s), 2.49 (1H, dd, *J* = 6.0 Hz), 2.20 (1H, qd, *J* = 4.2, 7.2 Hz), 1.90 (3H, s), 1.88-1.82 (1H,m), 1.82-1.75 (1H, m), 1.41 (1H, dt, *J* = 3.6, 13.2 Hz), 1.26 (3H, d, *J* = 7.2 Hz), 1.25-1.19 (1H, m), 1.17 (3H, d, *J* = 7.2 Hz), 1.06 (3H, s), 1.00-0.94 (1H, q, *J* = 13.2 Hz), 0.94 (3H, d, *J* = 7.2 Hz), 0.86 (3H, d, *J* = 7.2 Hz);

^{13}C NMR (150 MHz, $CDCl_3$) δ : 223.7, 218.2, 172.0, 159.2, 137.1, 131.0, 129.3, 126.0, 113.8, 83.4, 81.0, 71.4, 55.3, 52.0, 51.5, 49.8, 48.5, 48.4, 47.8, 41.4, 41.3, 41.0, 40.7, 39.0, 31.6, 26.3, 22.7, 21.0, 20.3, 19.7, 19.6, 19.3, 16.5;

ESI-MS(positive) for calcd for $C_{34}H_{46}NaO_6S_2$[M+Na]$^+$ 637.2628, found 637.2630.

Methyl (2*R*,3*R*,4a*R*,4b*S*,6*R*,8*S*,8a*R*,9*R*,9a*S*,10*S*,10a*S*)-9-((4-methoxybenzyl)oxy)-2,3,4,6,8,10a-hexamethyl-1-oxo-1,2,3,4a,4b,5,6,7,8,8a,9,9a,10,10a-tetradecahydrocyclopenta[*b*]fluorene-10-carboxylate (34)

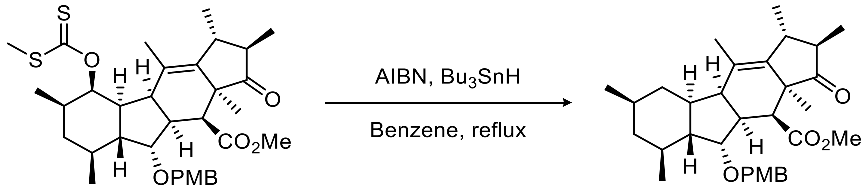

To a stirred solution of the starting material (9.1 mg, 0.0148 mmol) in Benzene (1.5 mL) were added Bu_3SnH (0.02 mL, 0.07 mmol) and AIBN (0.24 mg, 0.0015 mmol). The reaction mixture was stirred at 100 ℃ in an oil bath for 2 h. The reaction mixture was cooled to room temperature. Then, the organic solvent was removed by evaporation. The residue was purified by column chromatography (silica gel, hexane/EtOAc = 20:1) to give

the titled compound (6.7 mg, 90% yield) as a colorless oil.

$[\alpha]_D^{24}$ = + 78.4 (c = 1.72, $CHCl_3$);

IR (ATR, $CHCl_3$) ν_{max} 2948, 2926, 2868, 1732, 1612, 1586, 1512, 1455, 1371, 1301, 1246, 1192, 1168, 1084, 1070, 1033, 994, 960, 823, 756, 579 cm^{-1};

^1H NMR (600 MHz, $CDCl_3$) δ : 7.29-7.27 (2H, m), 6.89-6.87 (2H, m), 4.50 (2H, m), 4.05 (1H, d, J = 3.6 Hz), 3.80 (3H, s), 3.50 (3H, s), 2.97 (1H, t, J = 9.6 Hz), 2.80 (1H, d, J = 6.6 Hz), 2.67-2.66 (2H, m), 2.27-2.25 (1H, m), 2.15 (1H, m), 2.04 (1H, m),1.81 (3H, s), 1.62 (2H, m), 1.39 (1H, m), 1.32 (3H, d, J = 7.2 Hz), 1.15 (3H, d, J = 7.8 Hz), 1.09 (3H, s), 0.87 (3H, d, J = 6.6 Hz), 0.79 (3H, d, J = 6.6 Hz), 0.69 (1H, m), 0.44-0.36 (2H, m);

^{13}C NMR (150 MHz, $CDCl_3$) δ : 174.0, 159.1, 136.7, 131.2, 129.2, 127.7, 113.7, 82.0, 71.3, 55.2, 51.3, 49.1, 48.2, 44.7, 44.5, 42.5, 42.5, 40.4, 39.4, 33.9, 31.5, 25.3, 25.1, 22.5, 21.4, 19.9, 18.3, 15.6;

ESI-MS(positive) for calcd for $C_{32}H_{44}KO_5[M+K]^+$ 547.2820, found 547.2820.

Methyl (3*S*,4a*R*,4b*S*,6*R*,8*S*,8a*R*,9*R*,9a*S*,10*S*,10a*S*)-9-((4-methoxybenzyl)oxy)-2,3,4,6,8,10a-hexamethyl-1-(((trifluoromethyl)sulfonyl)oxy)-3,4a,4b,5,6,7,8,8a,9,9a,10,10a-dodecahydrocyclopenta[*b*]fluorene-10-carboxylate) (35)

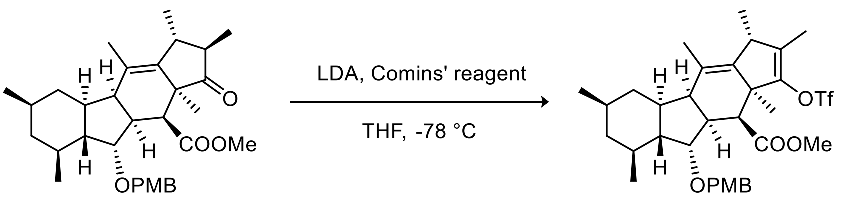

To a stirred solution of the starting material (3.5 mg, 0.007 mmol) in anhydrous THF

(0.5 mL) at -78 °C was added a solution of LDA (0.3 M in THF, 0.45 mL, 0.126 mmol). After stirring for 40 min, the resulting mixture was added a solution of Comins' reagent (27.4 mg, 0.07 mmol) in THF (0.2 mL) dropwise and stirred for 30 min. The reaction mixture was quenched with saturated aqueous NH_4Cl at -78 °C. The mixture was extracted with EtOAc. The organic layer was washed with water and brine, dried over anhydrous Na_2SO_4, and concentrated *in vacuo*. The residue was purified by column chromatography (silica gel, hexane/EtOAc = 50:1) to give the titled compound (3.8 mg, 86% yield) as a colorless oil.

$[\alpha]_D^{24}$ = - 16.9 (*c* = 0.79, $CHCl_3$);

IR (ATR, $CHCl_3$) ν_{max} 2950, 2868, 1741, 1612, 1513, 1456, 1408, 1374, 1248, 1212, 1164, 1140, 1079, 1037, 892, 837cm^{-1};

^{1}H NMR (600 MHz, $CDCl_3$) δ : 7.28 (2H, m), 6.89-6.87 (2H, m), 4.50 (2H, m), 3.87 (1H, d, *J* = 4.2 Hz), 3.80 (3H, s), 3.51 (3H, s), 3.13 (1H, q, *J* = 7.2 Hz), 2.91 (1H, m), 2.75 (1H, d, *J* = 7.2 Hz), 2.69 (1H, dd, *J* = 8.4, 7.2 Hz), 2.20-2.03 (1H, m), 2.08-2.03 (1H, m), 1.78 (3H, s), 1.73 (3H, s), 1.64-1.59 (2H, m), 1.42-1.34 (1H, m), 1.23 (3H, s), 1.22 (3H, d, *J* = 7.2 Hz), 0.93-0.82 (1H, m), 0.88 (3H, d, *J* = 6.6 Hz), 0.79 (3H, d, *J* = 6.0 Hz), 0.66-0.62 (1H, m), 0.51-0.45 (1H, m);

^{13}C NMR (150 MHz, $CDCl_3$) δ : 172.5, 159.2, 145.4, 136.0, 131.9, 131.1, 129.2, 127.5, 113.8, 82.3, 71.4, 55.3, 54.4, 51.0, 47.9, 47.6, 45.3, 44.2, 43.6, 43.3, 41.5, 39.7, 33.8, 31.3, 25.4, 22.6, 20.0, 18.1, 17.7, 10.8;

ESI-MS(positive) for calcd for $C_{33}H_{43}F_3NaO_7S$[M+Na]$^+$ 663.2574, found 663.2574.

Methyl (3*R*,4a*R*,4b*S*,6*R*,8*S*,8a*R*,9*R*,9a*S*,10*S*,10a*S*)-9-((4-methoxybenzyl)oxy)-2,3,4,6,8,10a-hexamethyl-3,4a,4b,5,6,7,8,8a,9,9a,10,10a-

dodecahydrocyclopenta[*b*]fluorene-10-carboxylate (5)

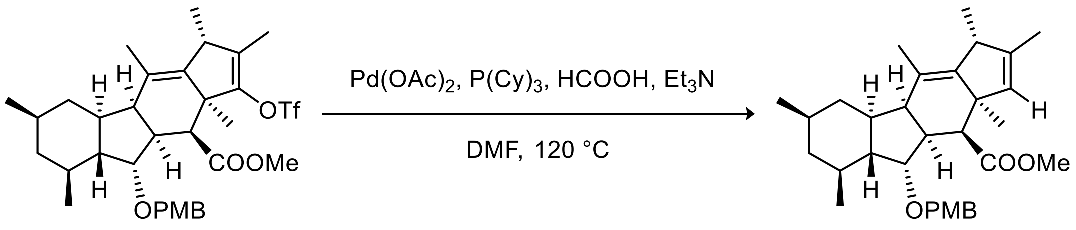

Formic acid (0.052 mL, 0.372 mmol) was added to a solution of enol triflate (120 mg, 0.186 mmol), palladium acetate (2.0 mg, 0.0088mmol), tricyclohexcylphosphine (0.6 M in toluene, 0.03 mL, 0.0176 mmol), and triethylamine (0.02 mL, 0.558 mmol) in DMF (18.6 mL), and the resulting mixture was heated at 120 ℃ in an oil bath for 1.5 h. The reaction mixture was cooled to room temperature and quenched with water. The mixture was extracted with EtOAc. The organic layer was washed with water and brine, dried over anhydrous Na_2SO_4, and concentrated *in vacuo*. The residue was purified by column chromatography (silica gel, hexane/EtOAc = 50:1) to give the titled compound (80 mg, 90% yield) as a colorless oil.

$[\alpha]_D^{24}$ = - 18.5 (*c* = 1.26, $CHCl_3$);

IR (ATR, $CHCl_3$) ν_{max} 2951, 2924, 2865, 1742, 1612, 1513, 1455, 1370, 1247, 1154, 1037, 891, 832 cm^{-1};

^1H NMR (600 MHz, $CDCl_3$) δ : 7.26 (2H, m), 6.87 (2H, m), 5.15 (1H, s), 4.52 (1H, d, *J* = 11.4 Hz), 4.35 (1H, d, *J* = 11.4 Hz), 3.80 (3H, s), 3.65 (1H, d, *J* = 4.8 Hz) 3.45 (3H, s), 3.00 (1H, brq, *J* = 7.2 Hz), 2.90 (1H, m), 2.85 (1H, d, *J* = 7.8 Hz), 2.75 (1H, dd, *J* = 8.4, 7.8 Hz), 2.12 (1H, m), 2.05 (1H, brd, *J* = 12.6 Hz), 1.81 (3H, s), 1.66 (3H, s), 1.66 (1H, m), 1.59 (1H, m), 1.35 (1H, m), 1.13 (3H, d, *J* = 7.2 Hz), 1.10 (3H, s), 1.07 (1H, ddd, *J* = 12.0, 12.0, 12.0 Hz), 0.94 (1H, m), 0.90 (3H, d, *J* = 6.6 Hz), 0.78 (3H, d, *J* = 6.6 Hz), 0.53 (1H, ddd, *J* =

12.0, 12.0, 12.0 Hz);

^{13}C NMR (150 MHz, $CDCl_3$) δ : 173.5, 159.0, 143.2, 141.6, 131.2, 130.0, 129.2, 126.5, 113.7, 83.0, 70.8, 55.3, 53.9, 52.0, 50.5, 49.4, 45.8, 45.7, 44.8, 44.2, 41.7, 39.8, 33.9, 31.3, 26.7, 22.7, 20.1, 18.7, 18.4, 14.7;

ESI-MS(positive) for calcd for $C_{32}H_{45}O_4[M+H]^+$ 493.3312, found 493.3311.

^{1}H NMR spectrum of compound **12** (600 MHz, $CDCl_3$)

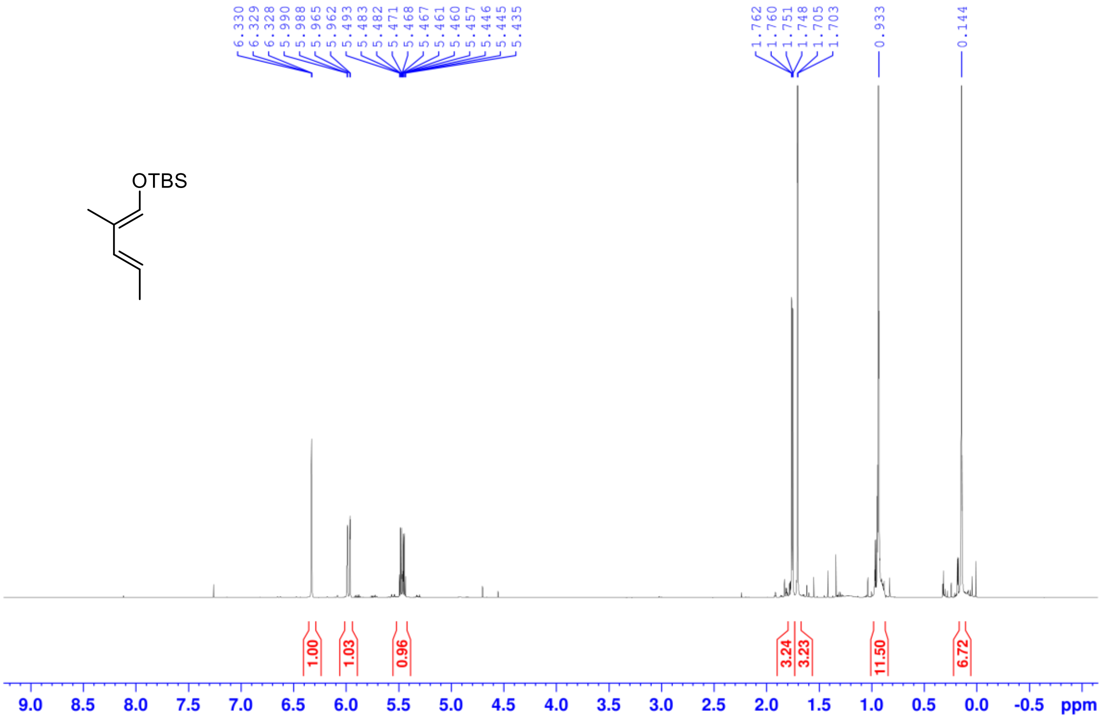

^{13}C NMR spectrum of compound **12** (150 MHz, $CDCl_3$)

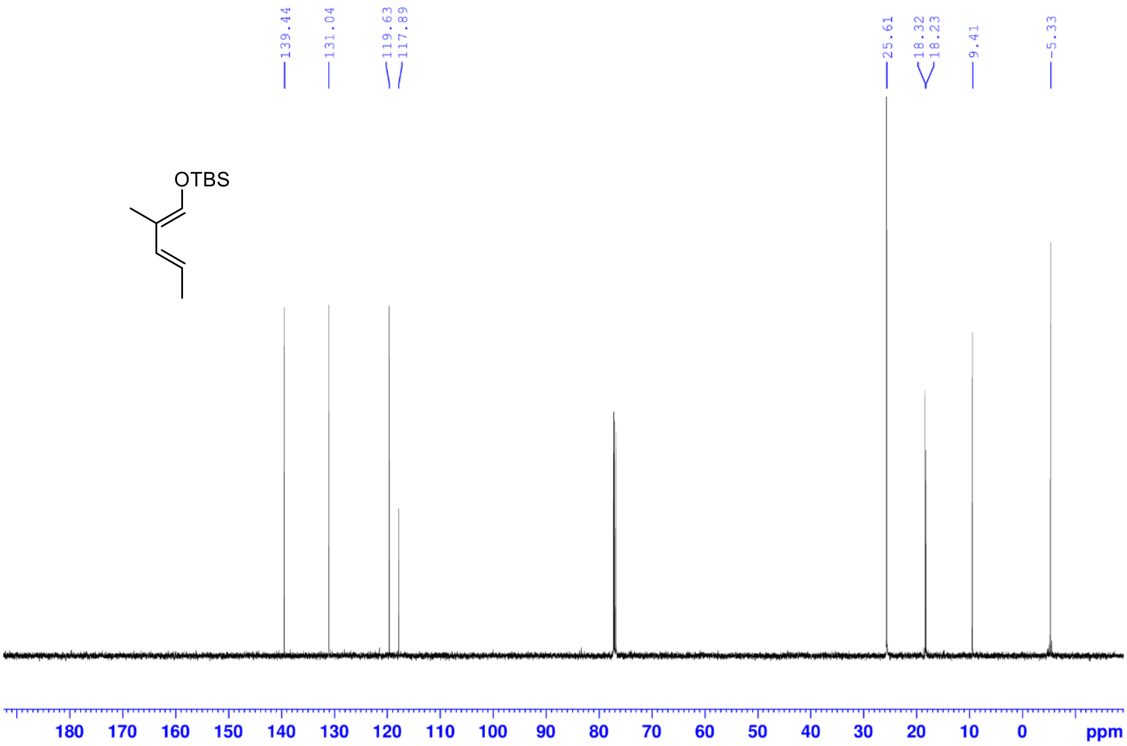

^{1}H NMR spectrum of compound **14** (600 MHz, $CDCl_3$)

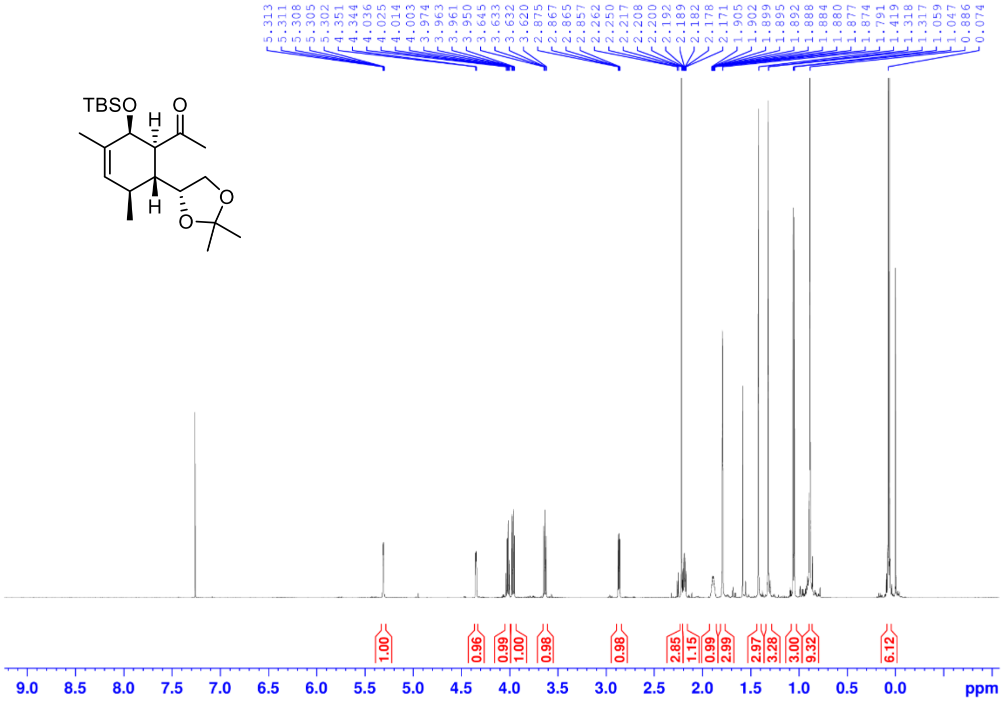

^{13}C NMR spectrum of compound **14** (150 MHz, $CDCl_3$)

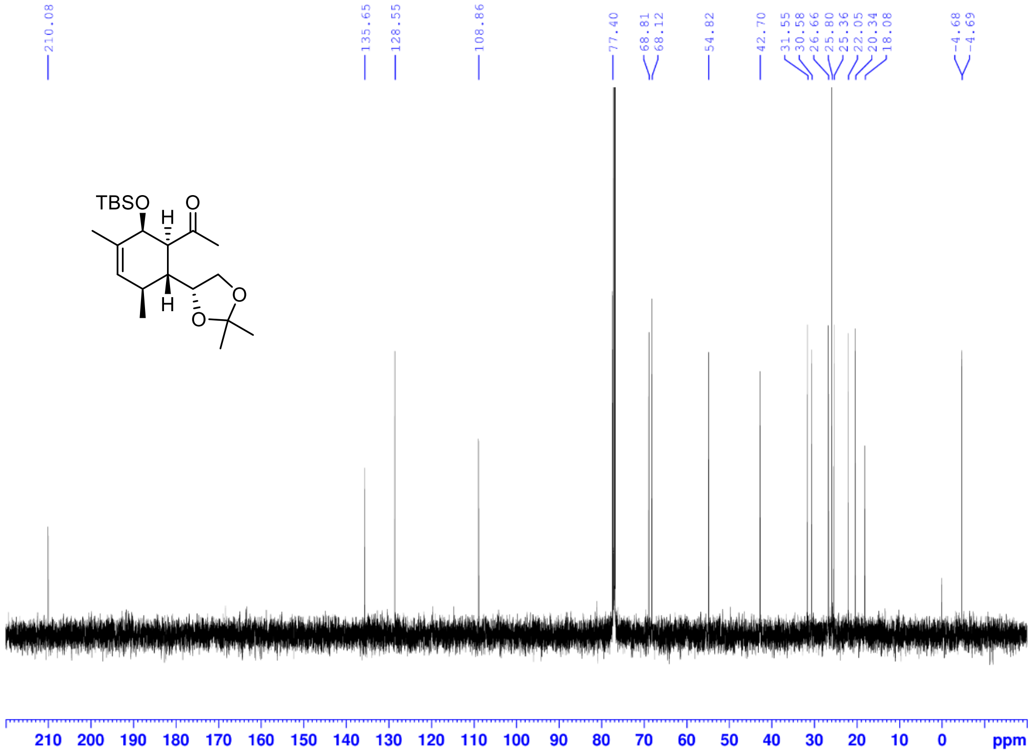

^{1}H NMR spectrum of compound **10** (600 MHz, $CDCl_3$)

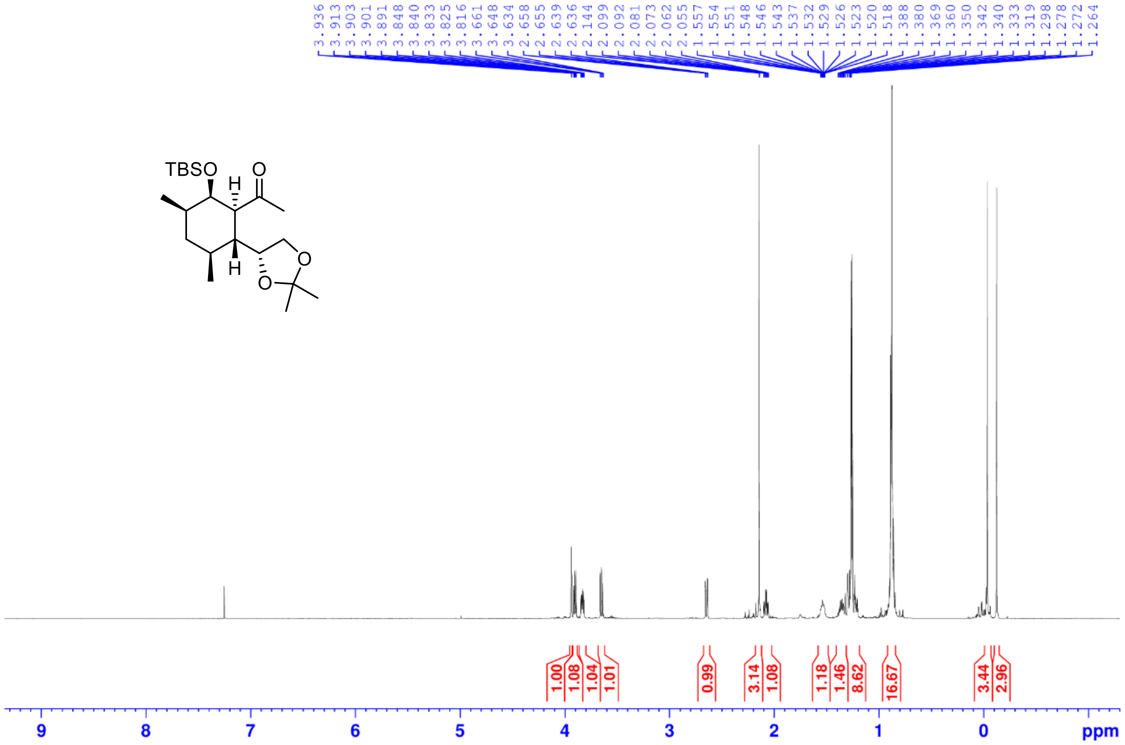

^{13}C NMR spectrum of compound **10** (150 MHz, $CDCl_3$)

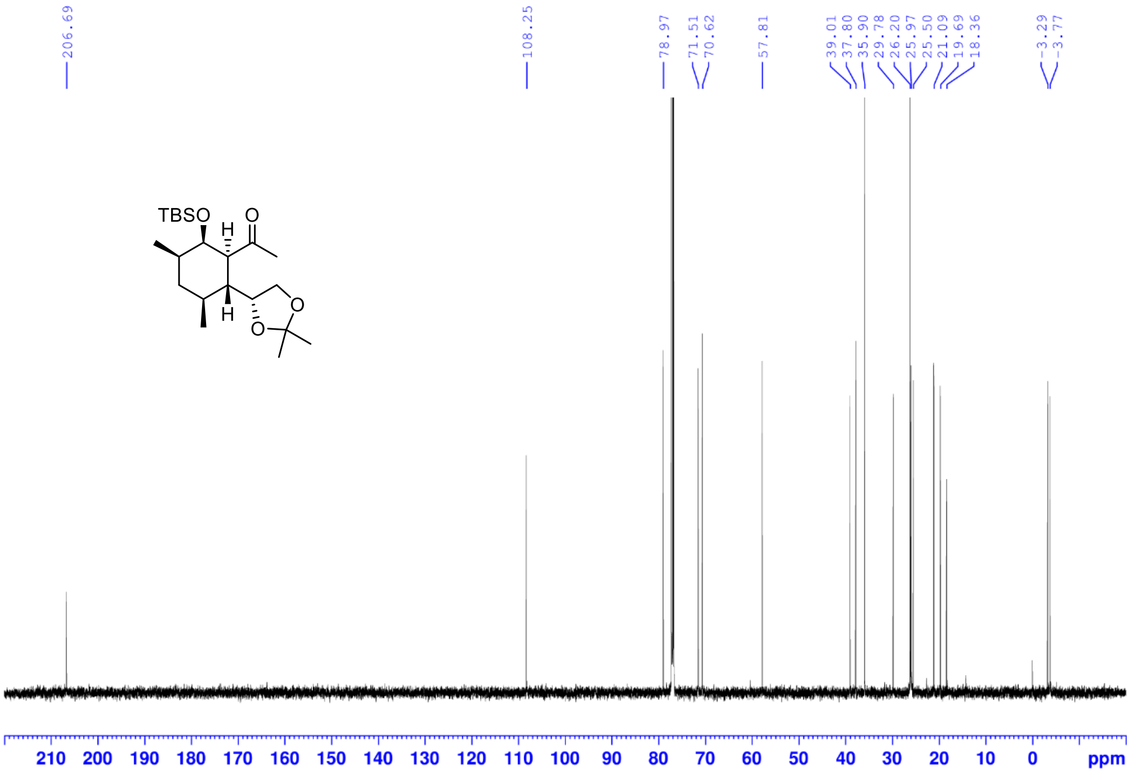

^{1}H NMR spectrum of compound **15** (600 MHz, $CDCl_3$)

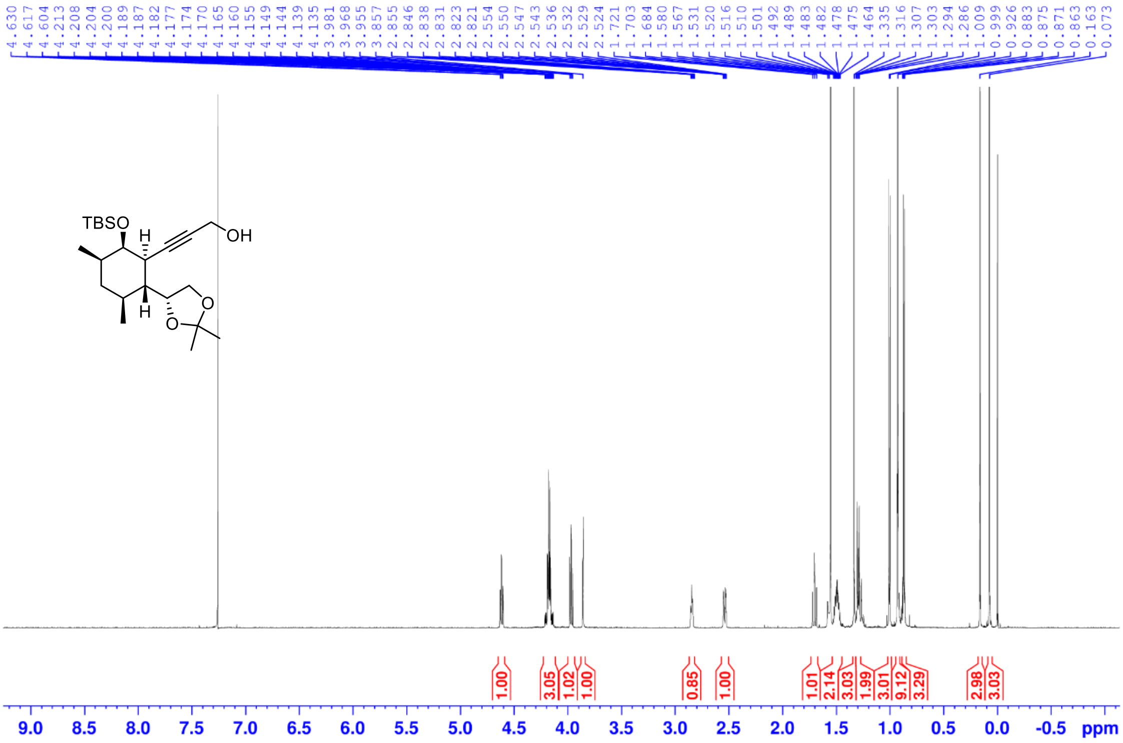

^{13}C NMR spectrum of compound **15** (150 MHz, $CDCl_3$)

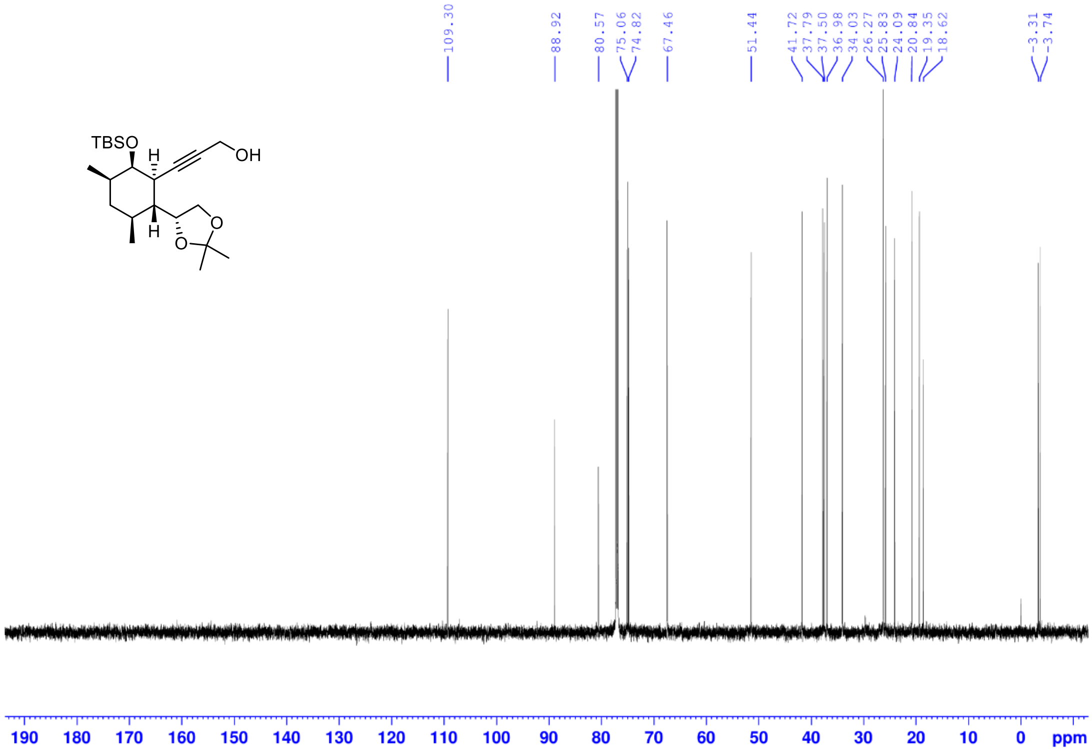

^{1}H NMR spectrum of compound **16** (600 MHz, $CDCl_3$)

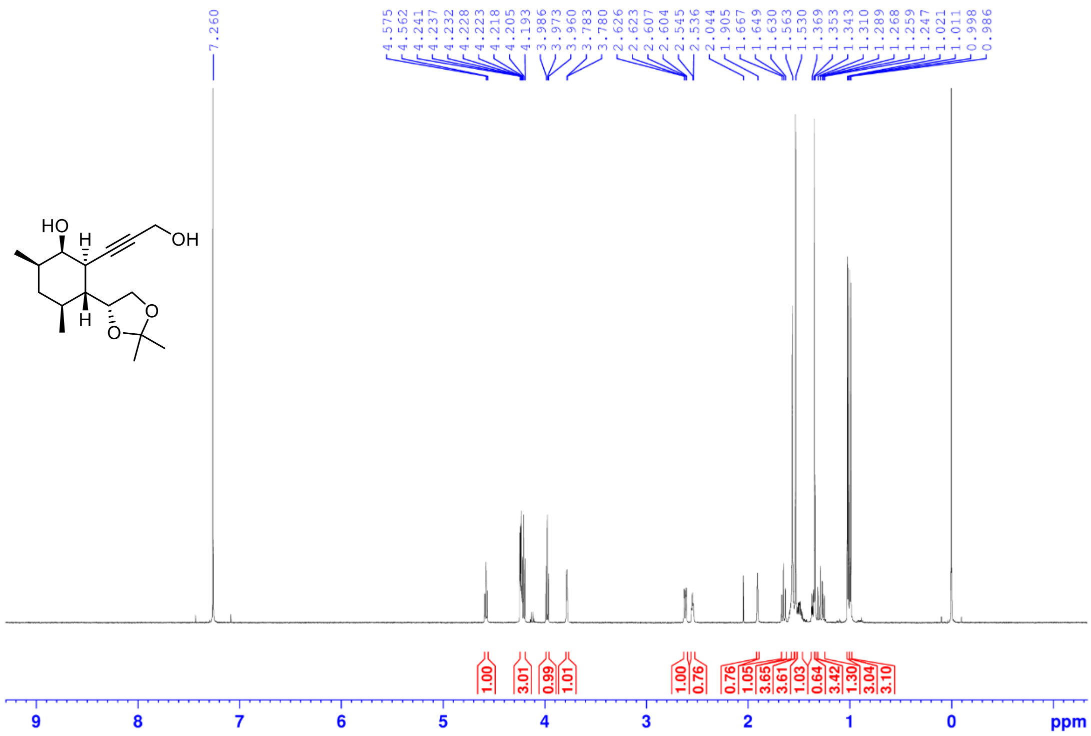

^{13}C NMR spectrum of compound **16** (150 MHz, $CDCl_3$)

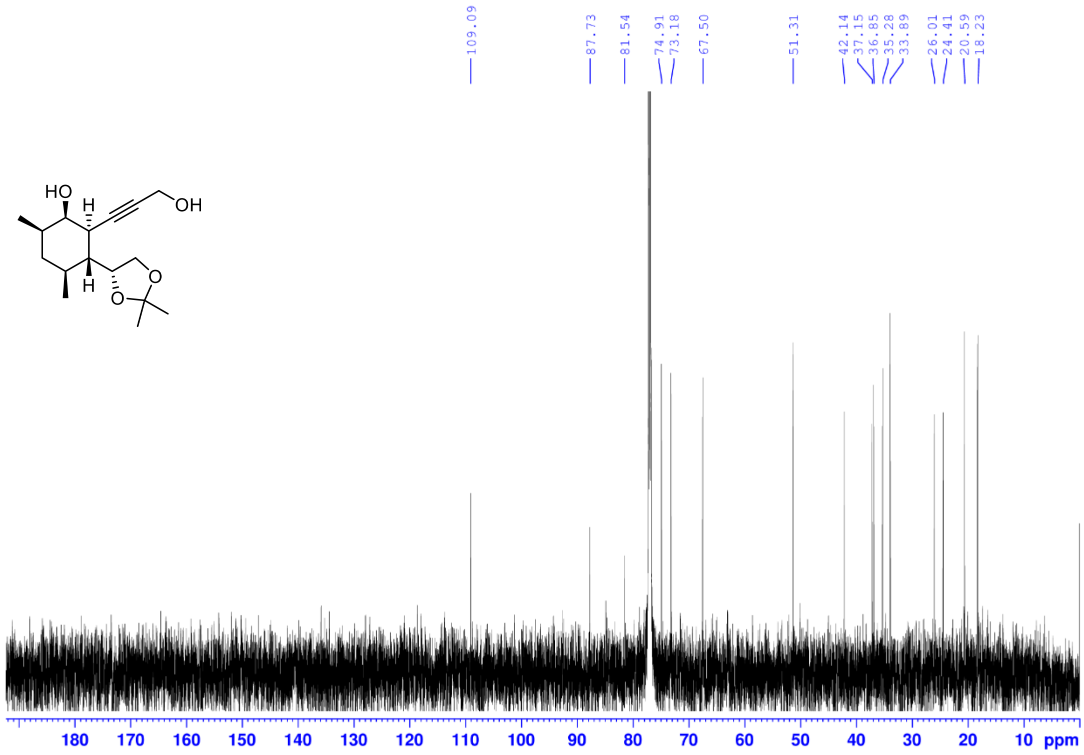

^{1}H NMR spectrum of compound **17** (600 MHz, $CDCl_3$)

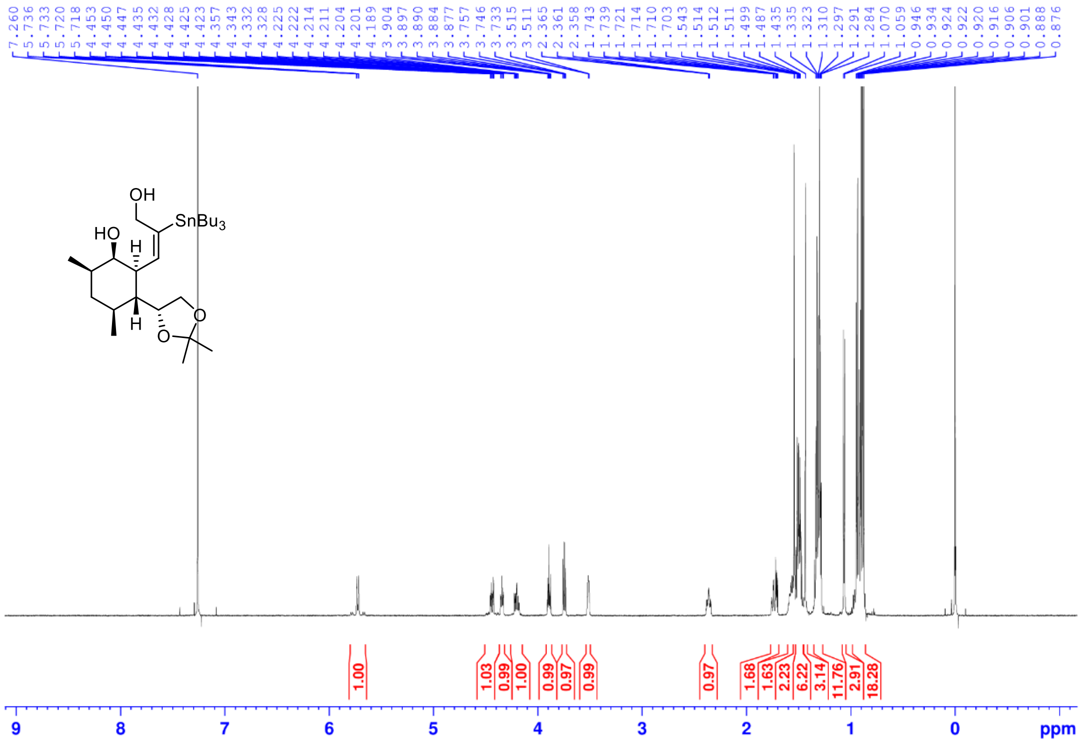

^{13}C NMR spectrum of compound **17** (150 MHz, $CDCl_3$)

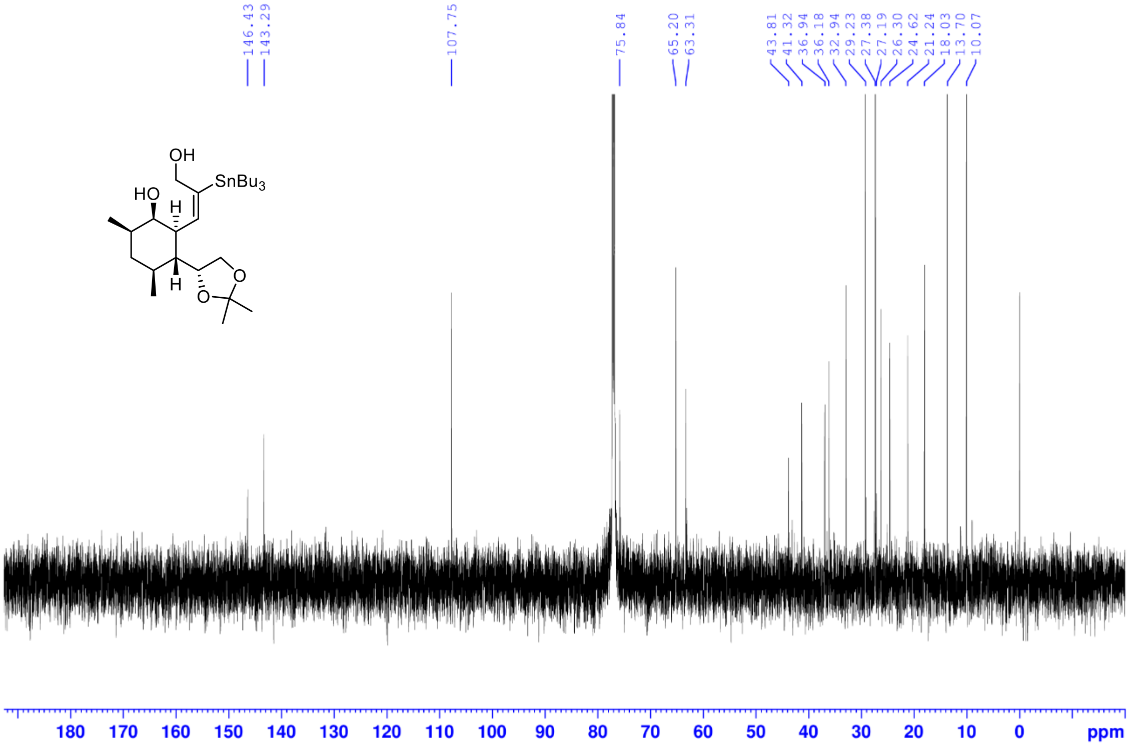

^{1}H NMR spectrum of compound **18** (600 MHz, $CDCl_3$)

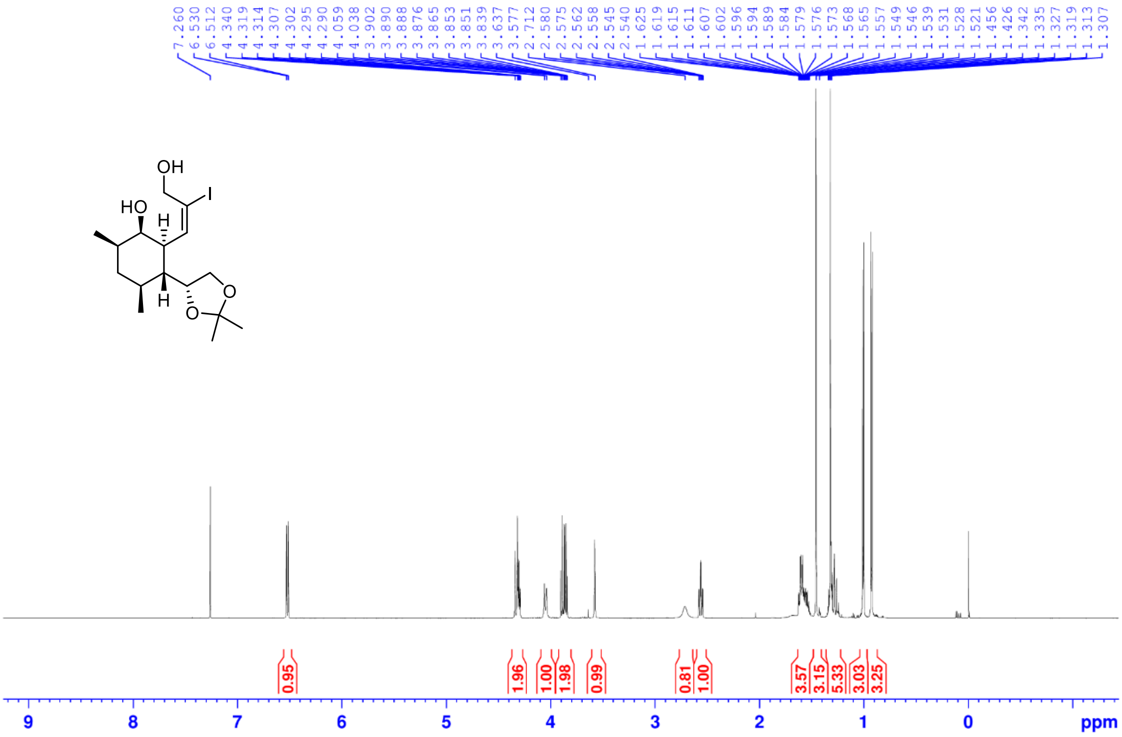

^{13}C NMR spectrum of compound **18** (150 MHz, $CDCl_3$)

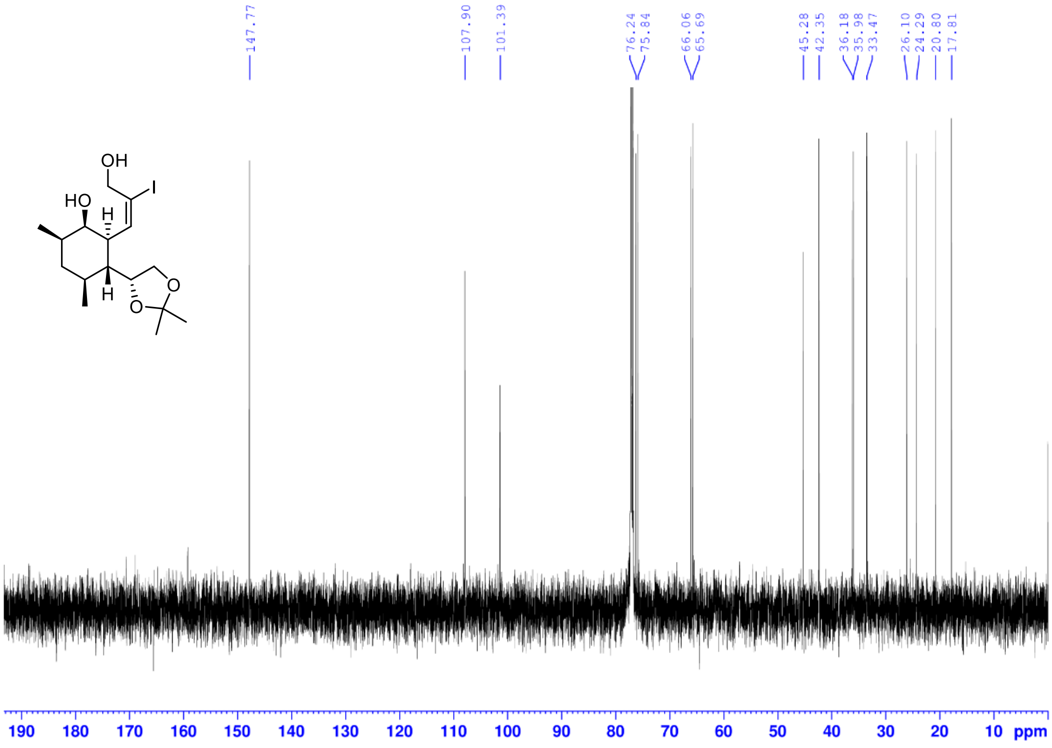

^{1}H NMR spectrum of compound **19** (600 MHz, $CDCl_3$)

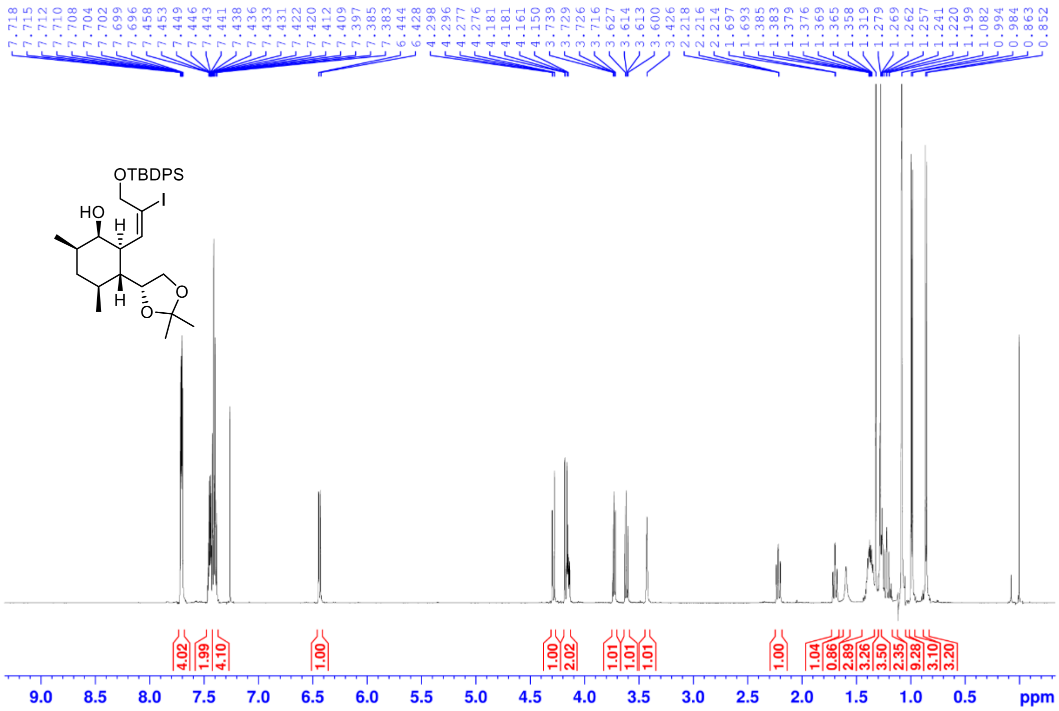

^{13}C NMR spectrum of compound **19** (150 MHz, $CDCl_3$)

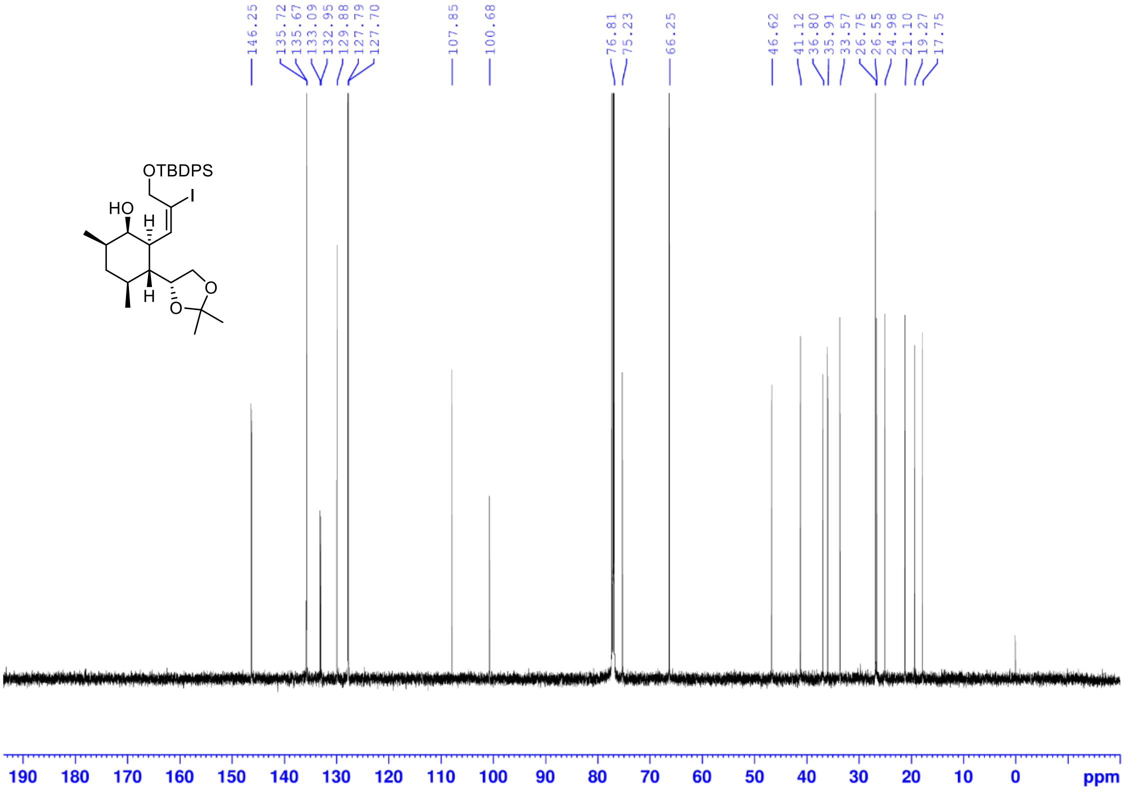

^{1}H NMR spectrum of compound **20** (600 MHz, $CDCl_3$)

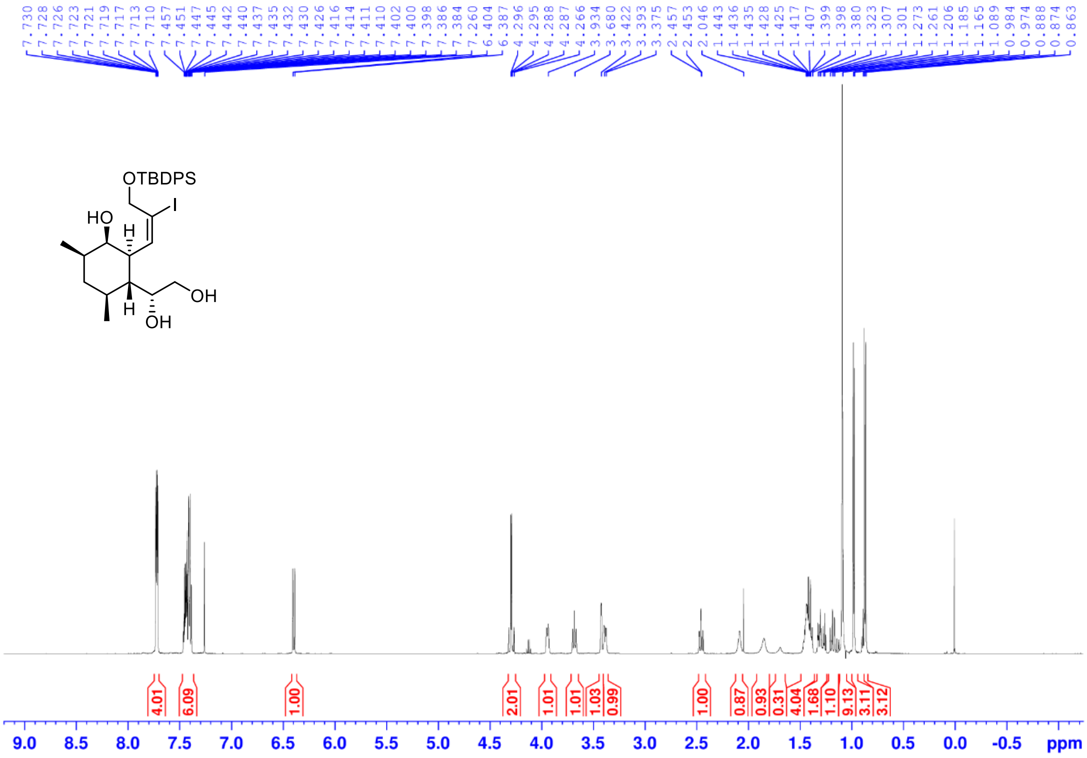

^{13}C NMR spectrum of compound **20** (150 MHz, $CDCl_3$)

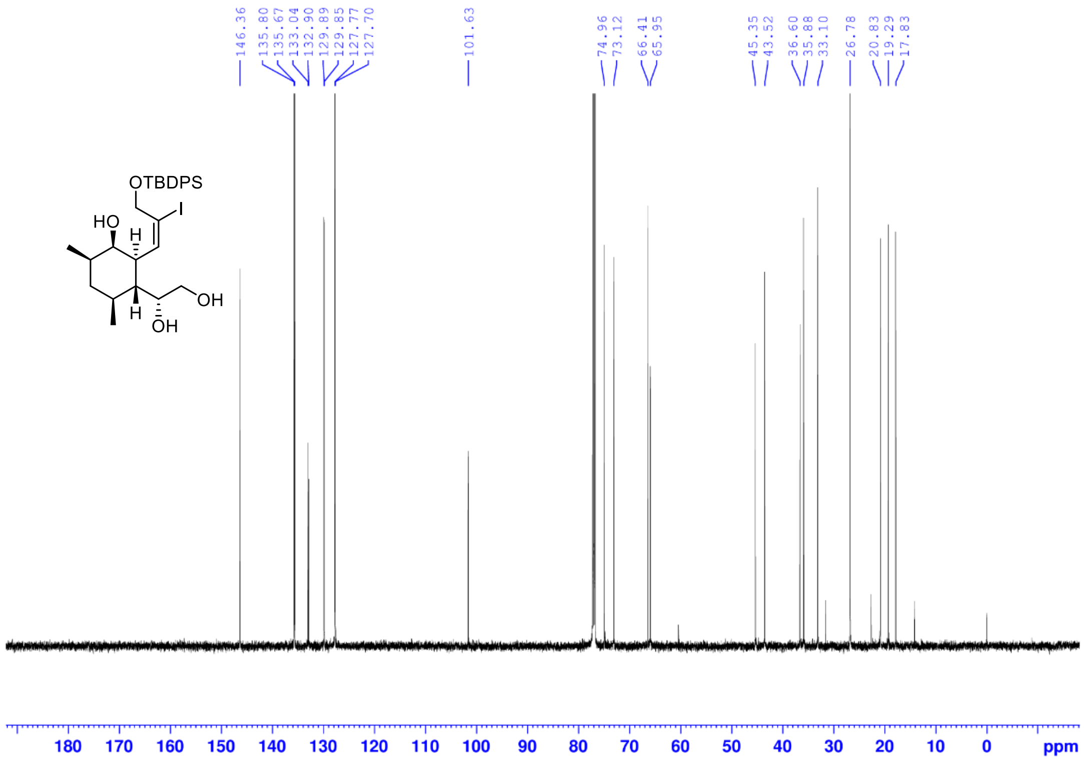

^{1}H NMR spectrum of compound **21** (600 MHz, $CDCl_3$)

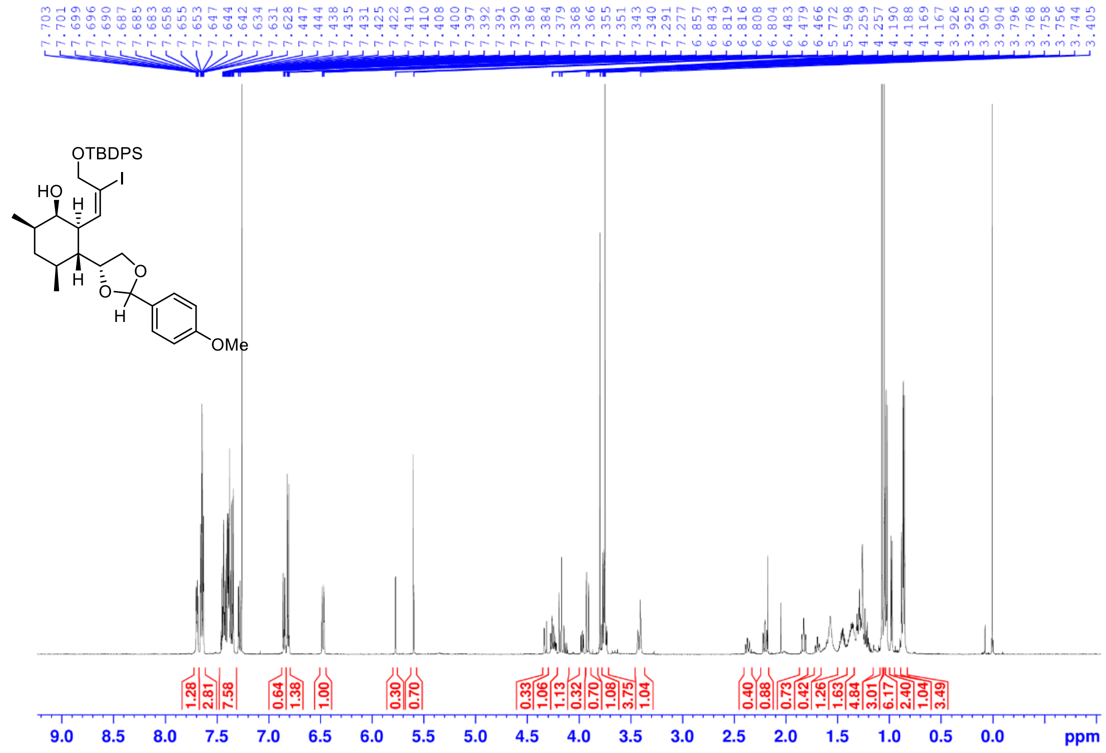

^{13}C NMR spectrum of compound **21** (150 MHz, $CDCl_3$)

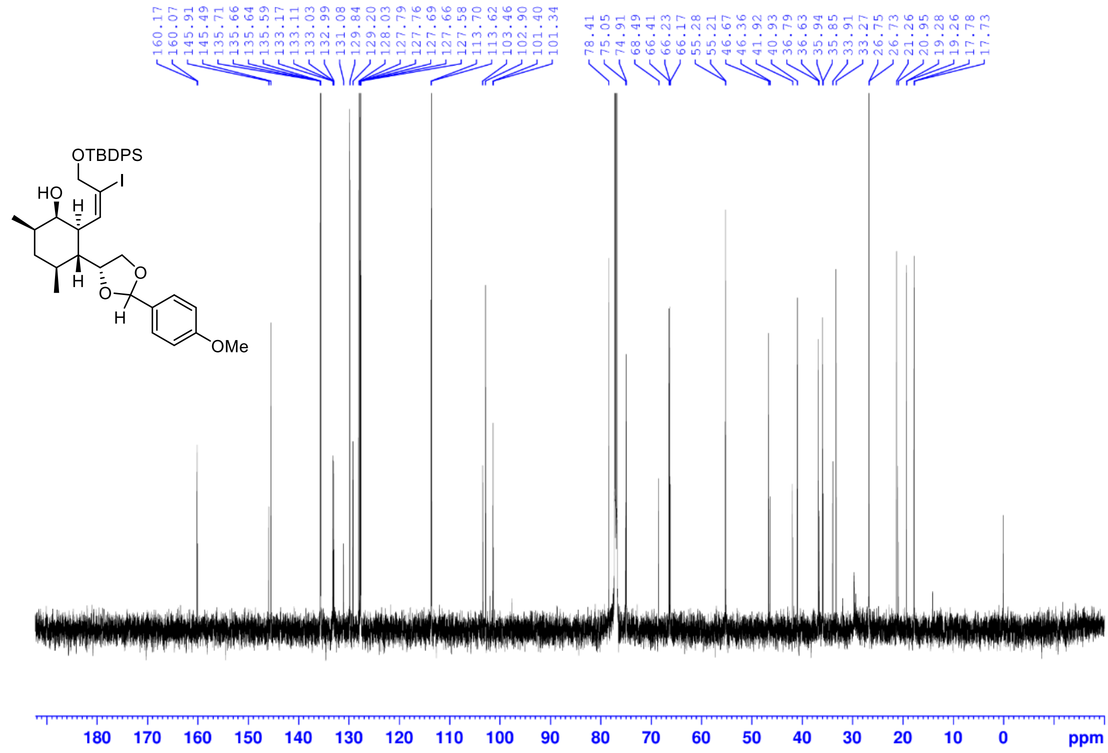

Mixture of two isomers at the benzylic position (dr = 2.33 : 1)

^{1}H NMR spectrum of compound **22** (600 MHz, $CDCl_3$)

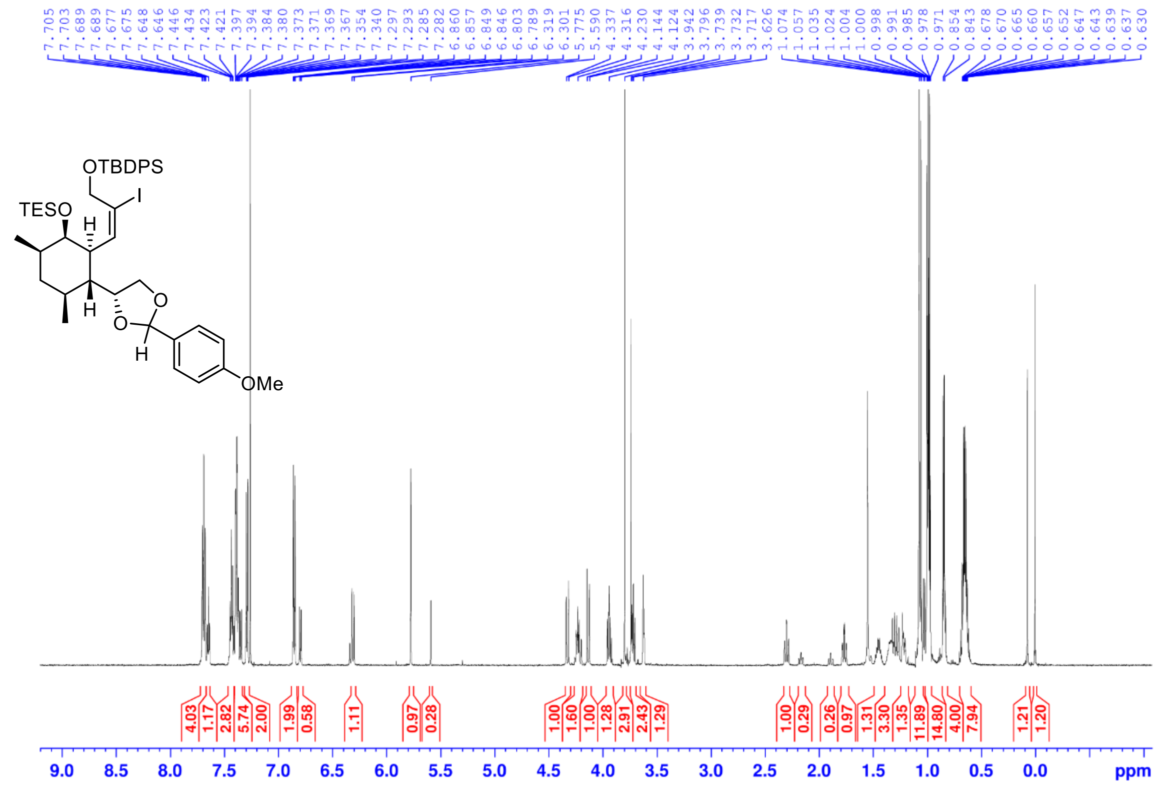

^{13}C NMR spectrum of compound **22** (150 MHz, $CDCl_3$)

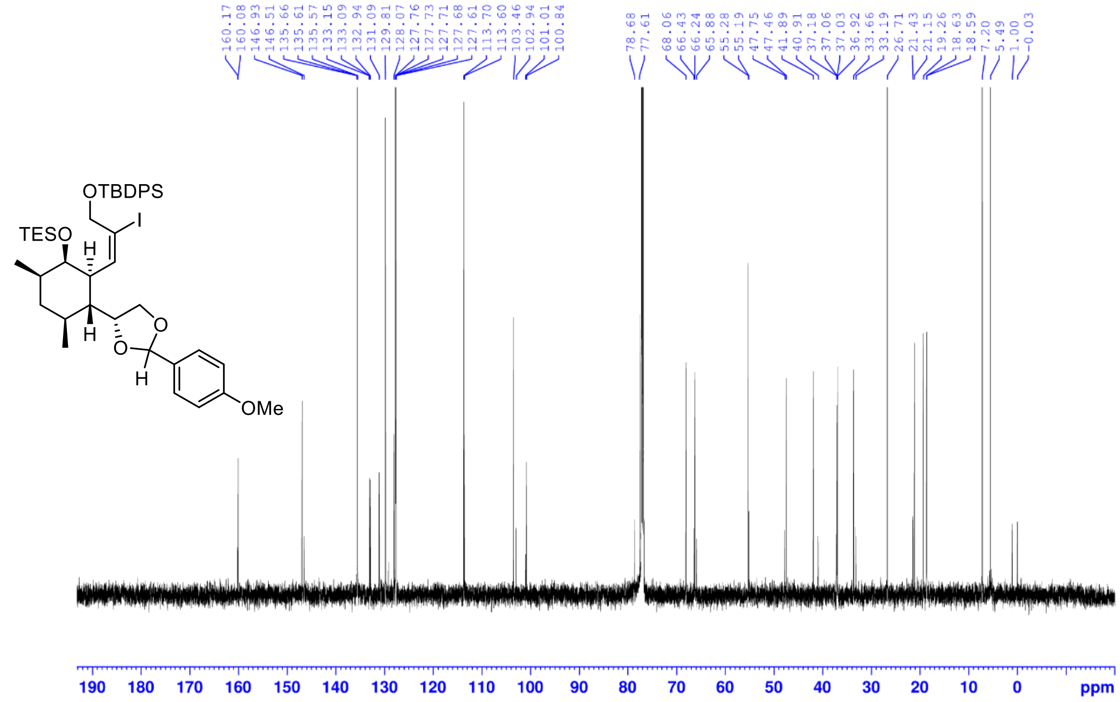

Mixture of two isomers at the benzylic position (dr = 2.55 : 1)

^{1}H NMR spectrum of compound **23** (400 MHz, $CDCl_3$)

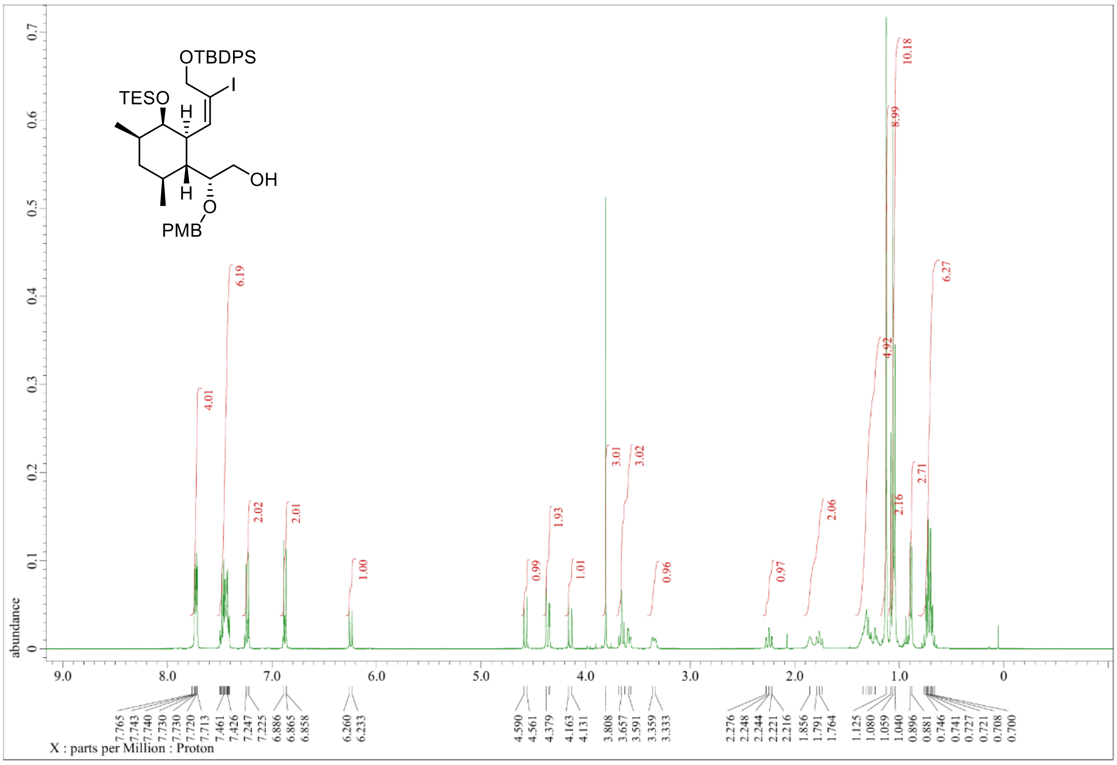

^{13}C NMR spectrum of compound **23** (100 MHz, $CDCl_3$)

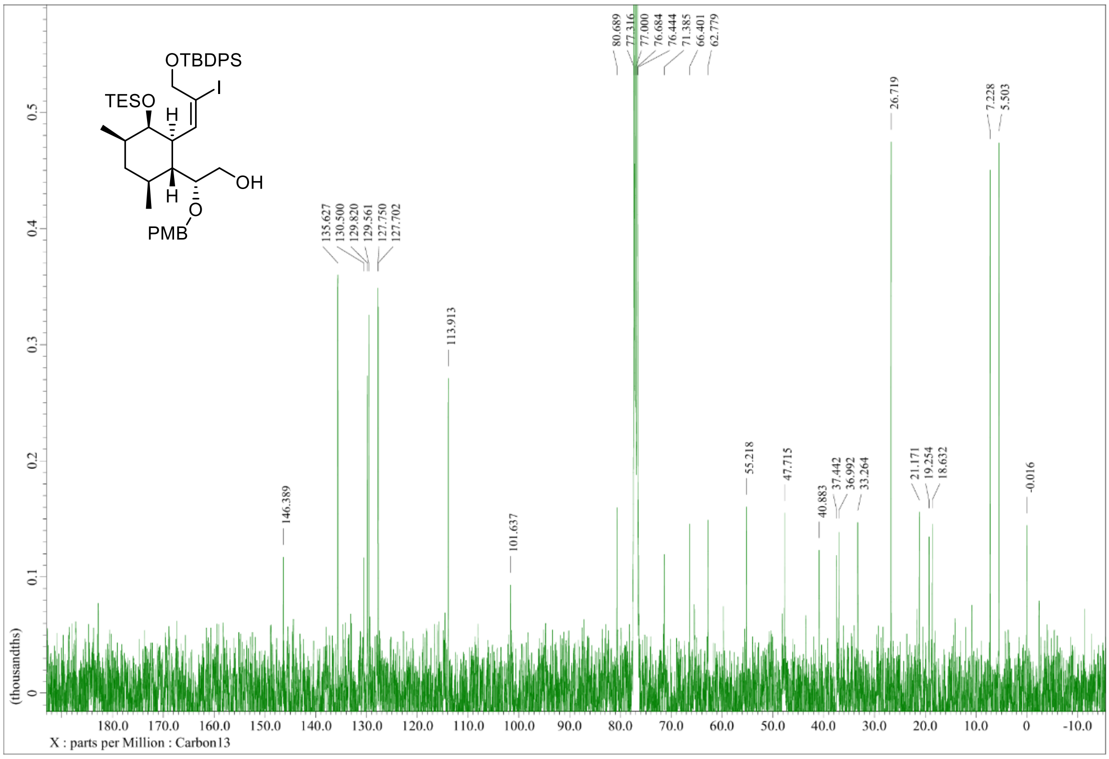

^{1}H NMR spectrum of compound **24** (600 MHz, $CDCl_3$)

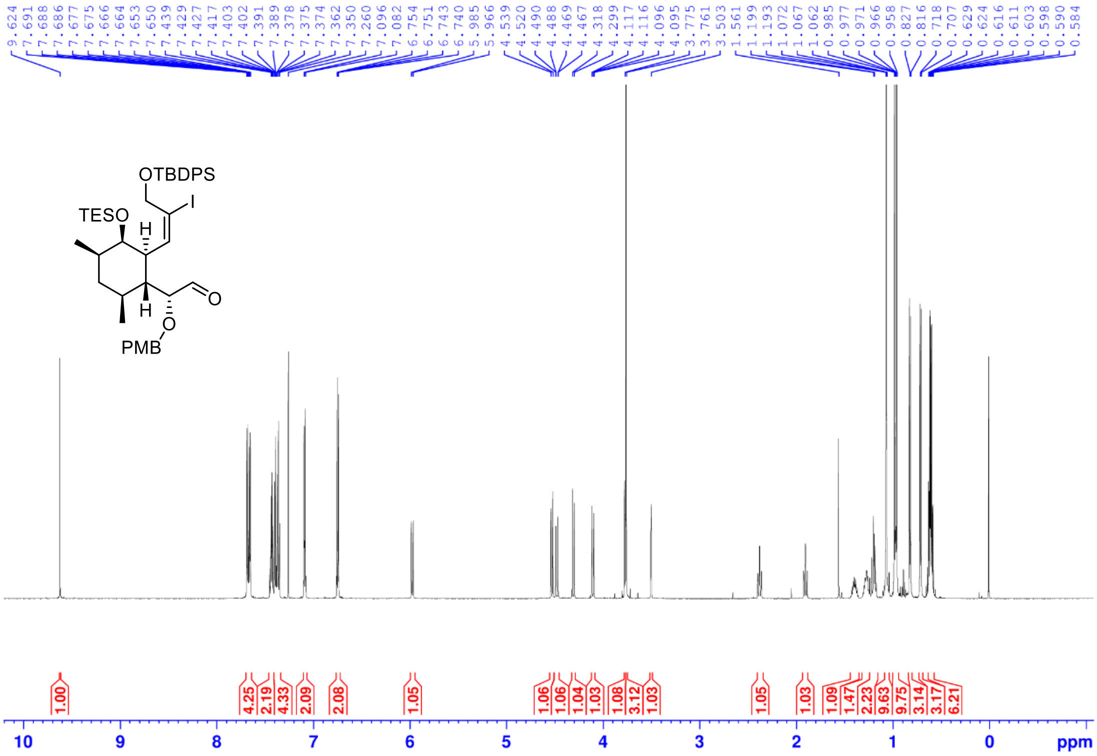

^{13}C NMR spectrum of compound **24** (150 MHz, $CDCl_3$)

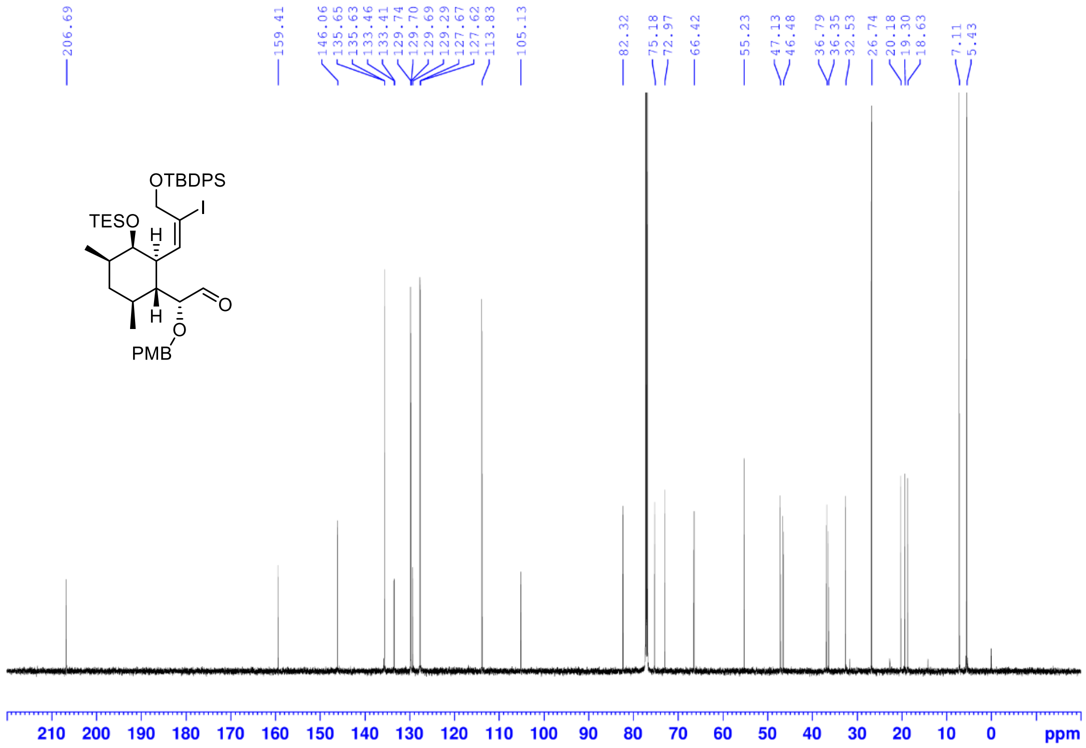

^{1}H NMR spectrum of compound **25** (600 MHz, $CDCl_3$)

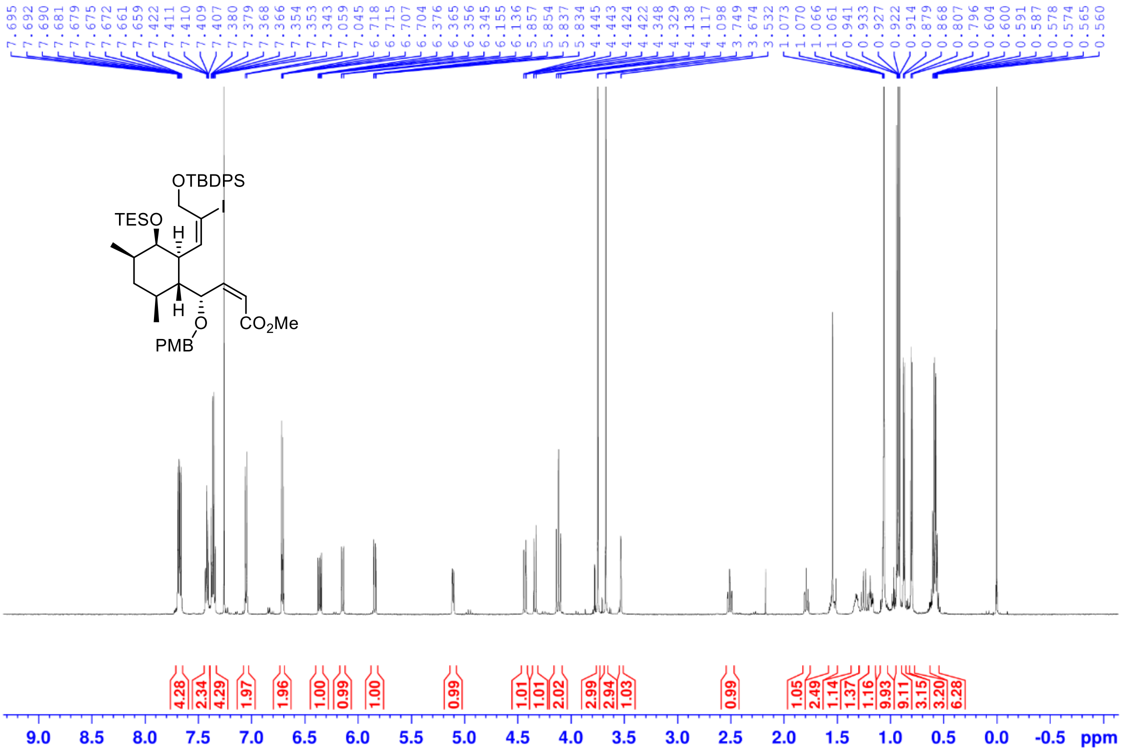

^{13}C NMR spectrum of compound **25** (150 MHz, $CDCl_3$)

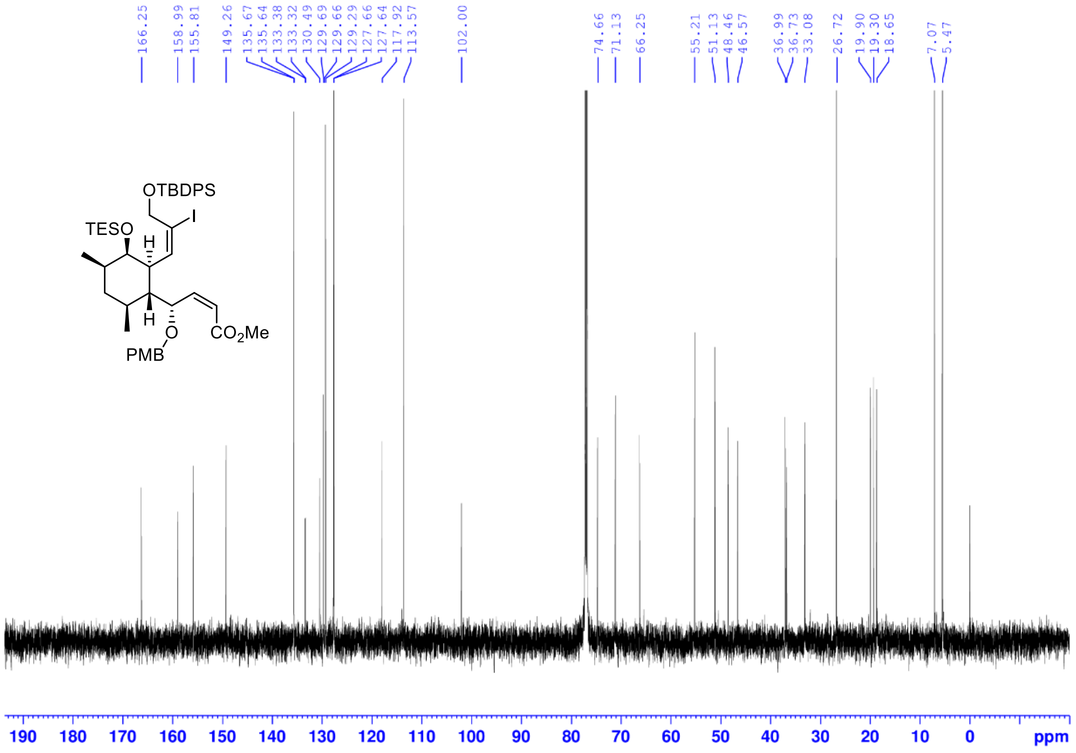

^{1}H NMR spectrum of compound **26** (600 MHz, $CDCl_3$)

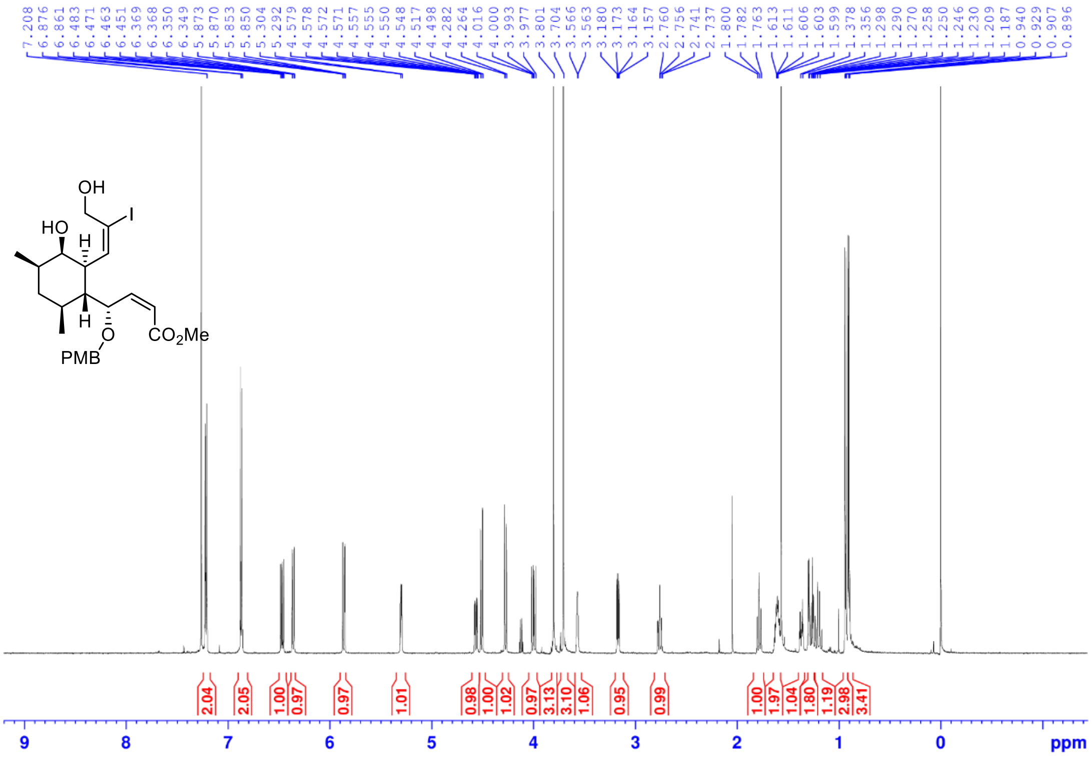

^{13}C NMR spectrum of compound **26** (150 MHz, $CDCl_3$)

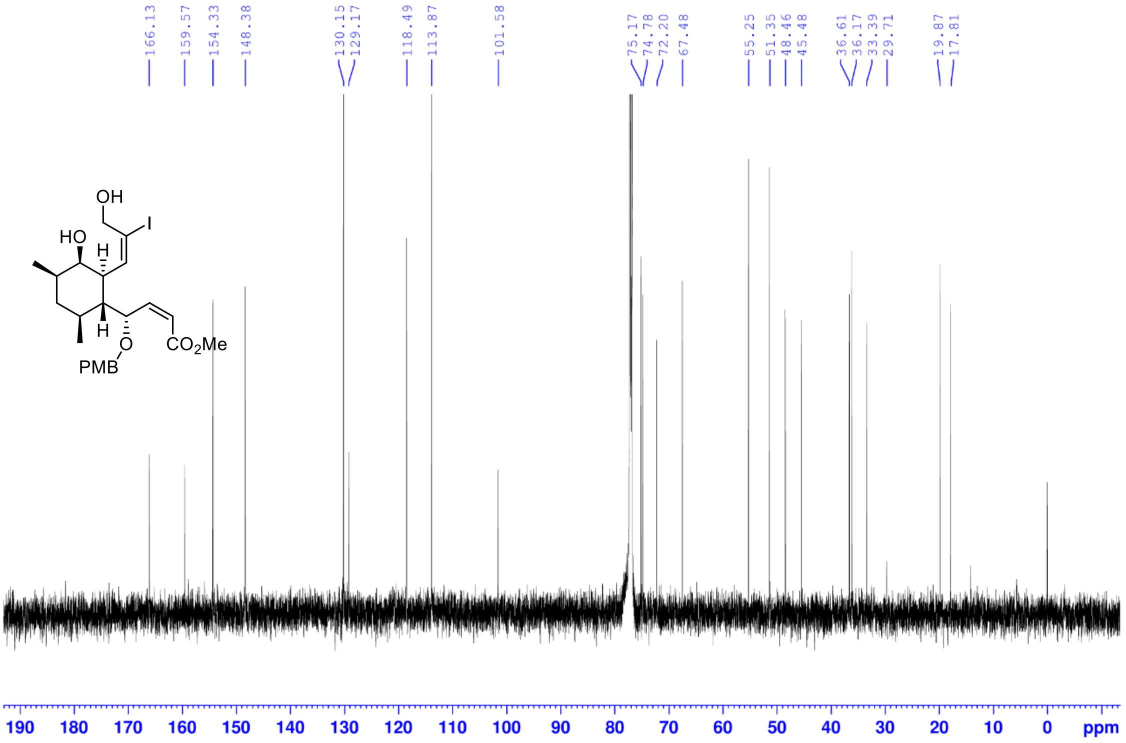

^{1}H NMR spectrum of compound **8** (600 MHz, $CDCl_3$)

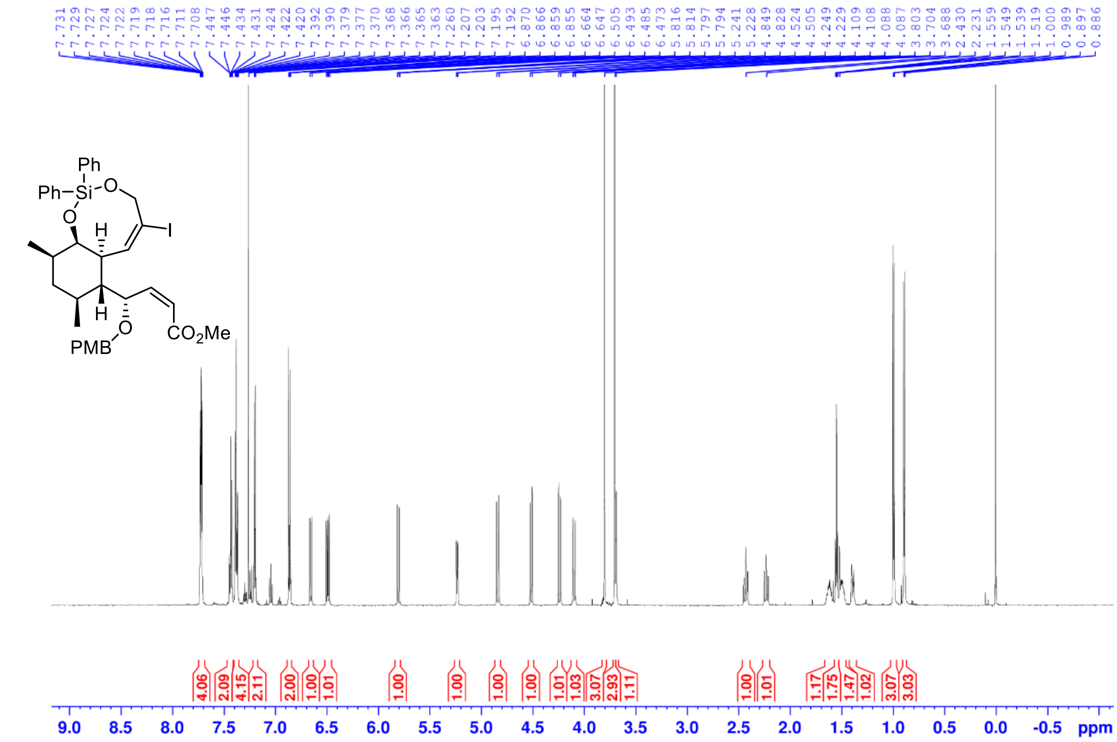

^{13}C NMR spectrum of compound **8** (150 MHz, $CDCl_3$)

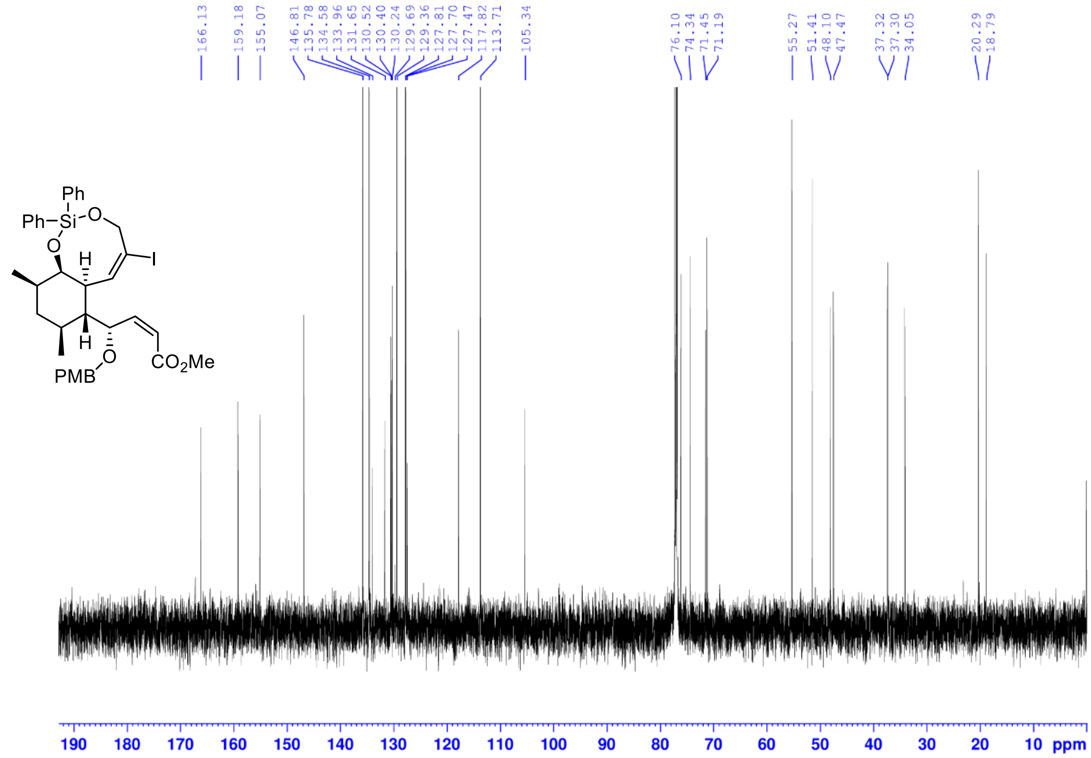

^{1}H NMR spectrum of compound **9** (600 MHz, $CDCl_3$)

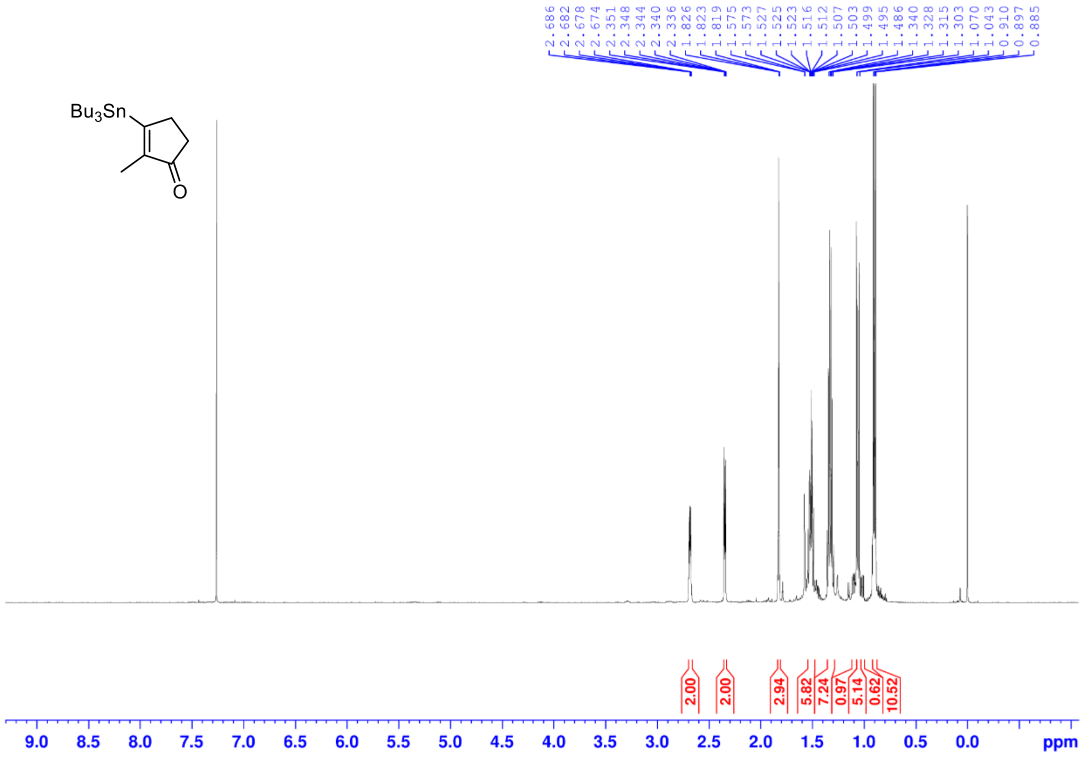

^{13}C NMR spectrum of compound **9** (150 MHz, $CDCl_3$)

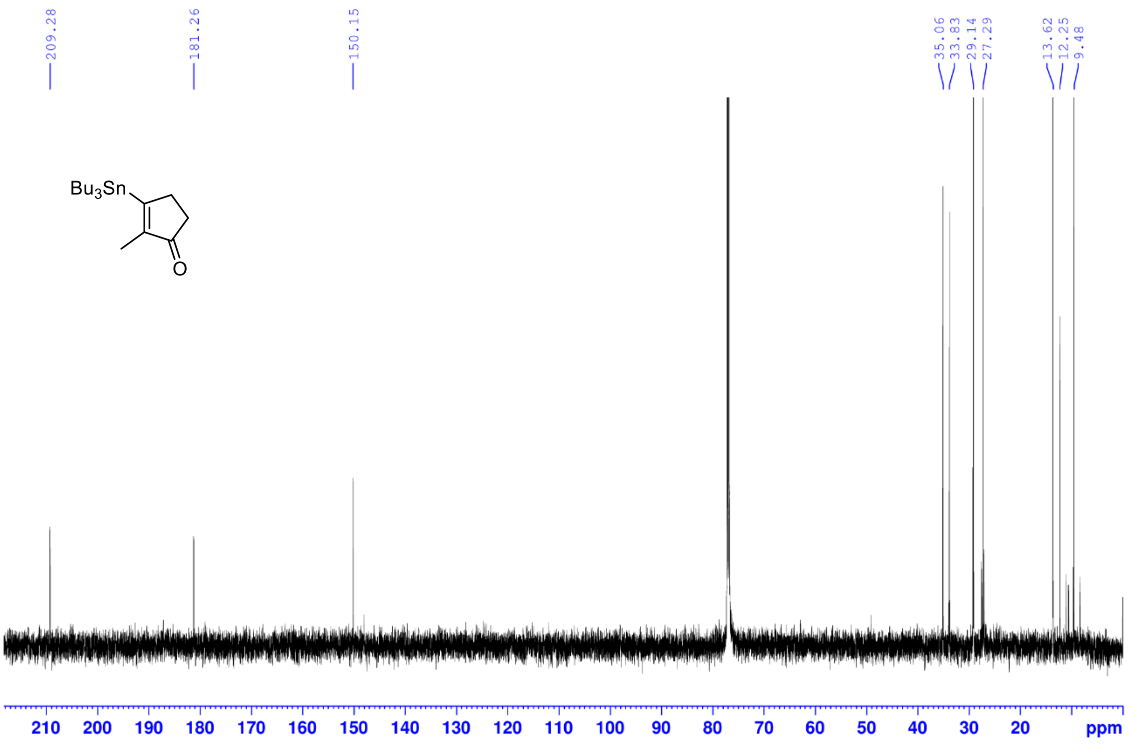

^{1}H NMR spectrum of compound **7** (600 MHz, $CDCl_3$)

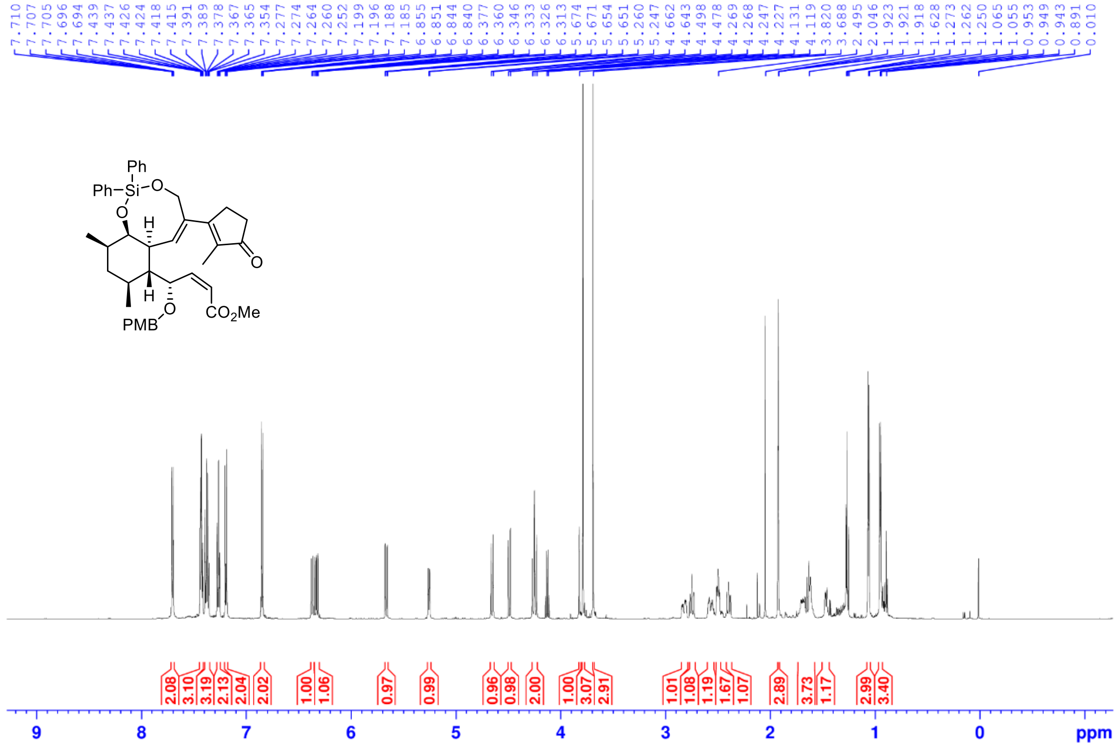

^{13}C NMR spectrum of compound **7** (150 MHz, $CDCl_3$)

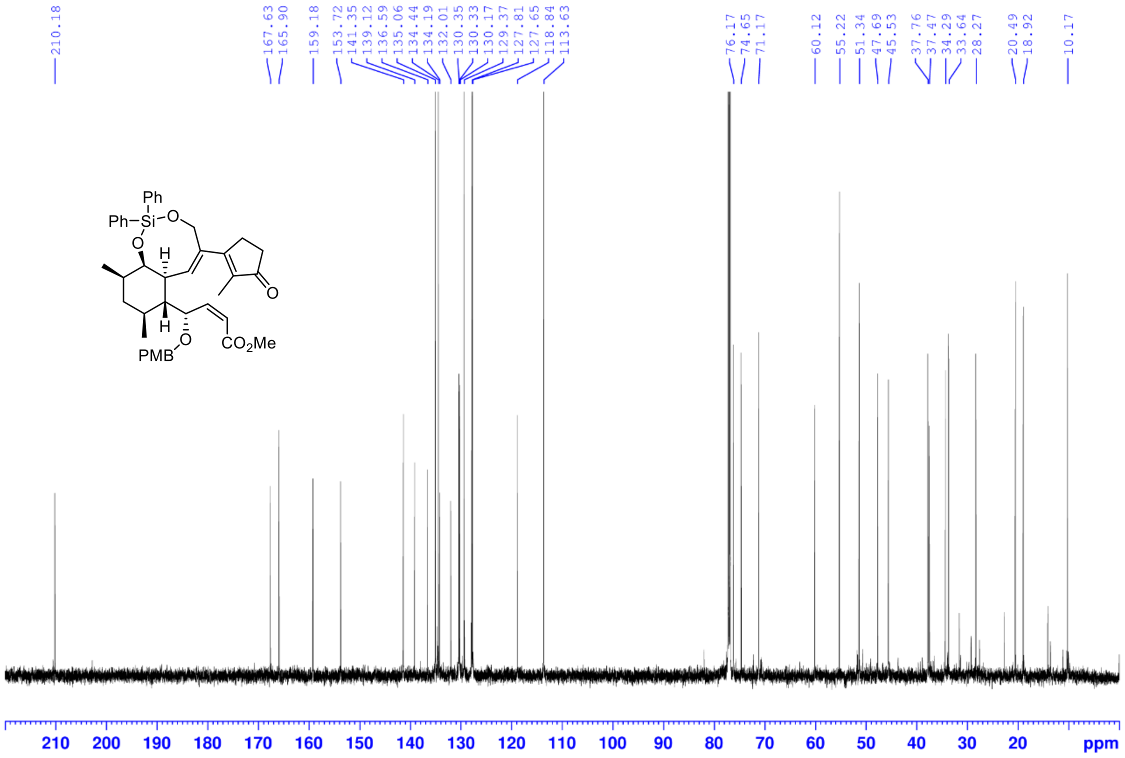

^{1}H NMR spectrum of compound **6** (600 MHz, $CDCl_3$)

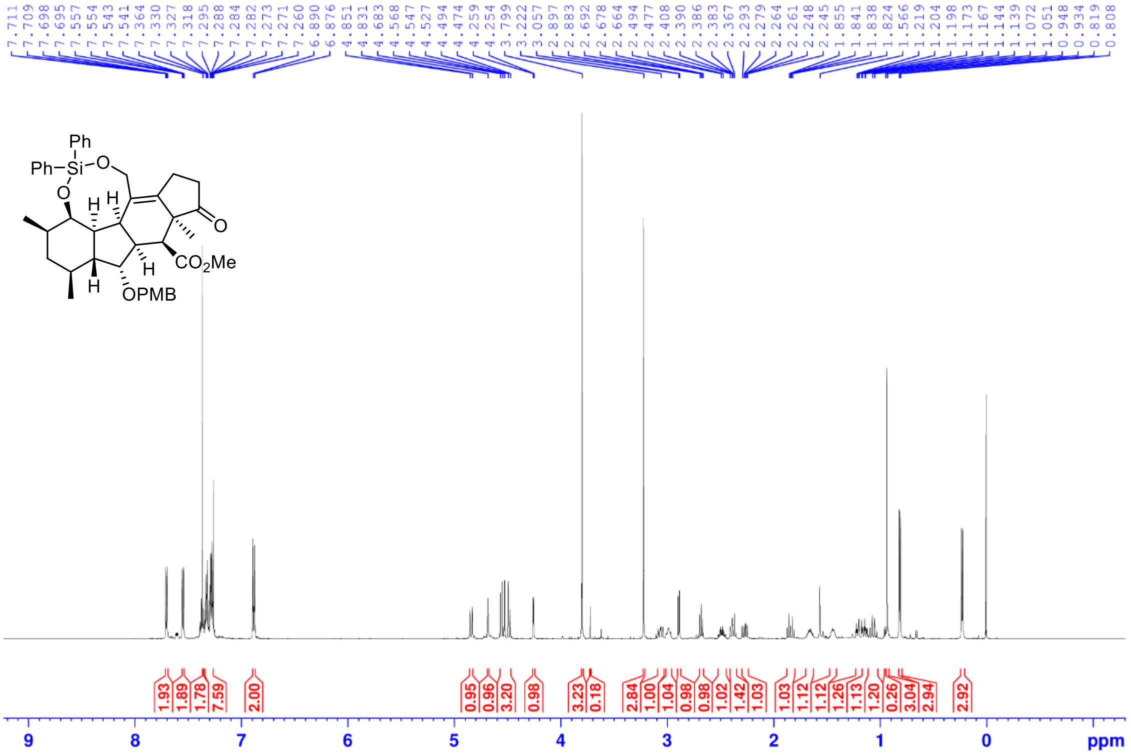

^{13}C NMR spectrum of compound **6** (150 MHz, $CDCl_3$)

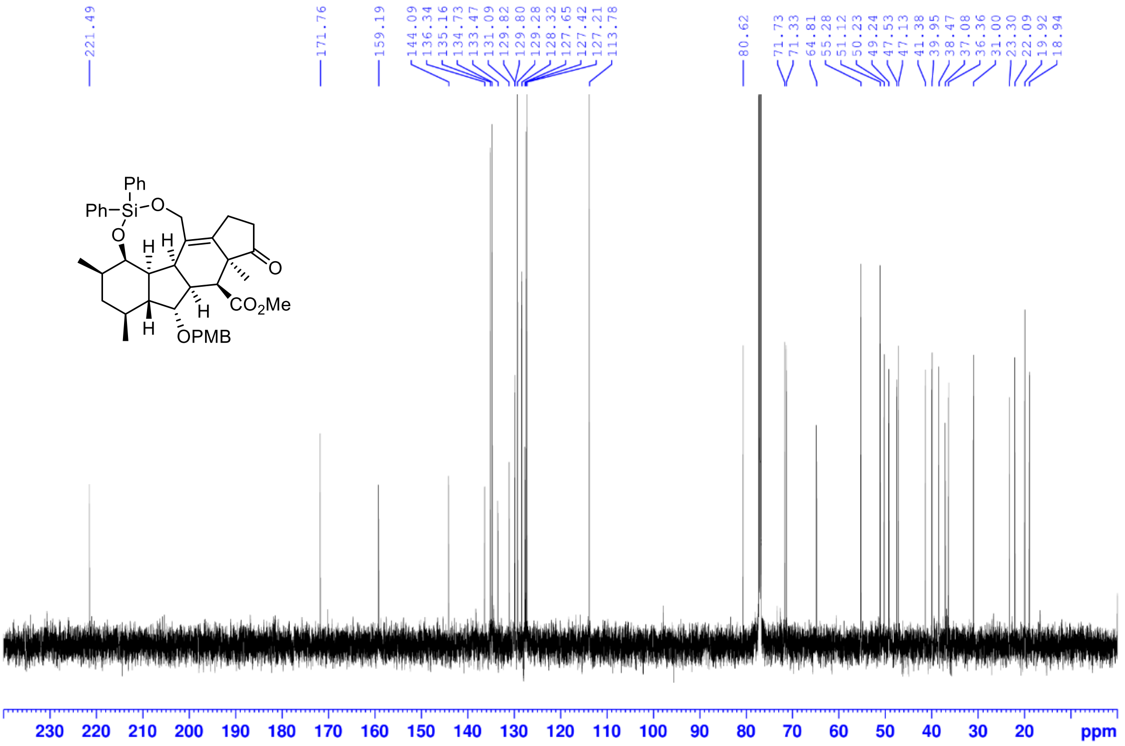

^{1}H NMR spectrum of compound **27** (600 MHz, $CDCl_3$)

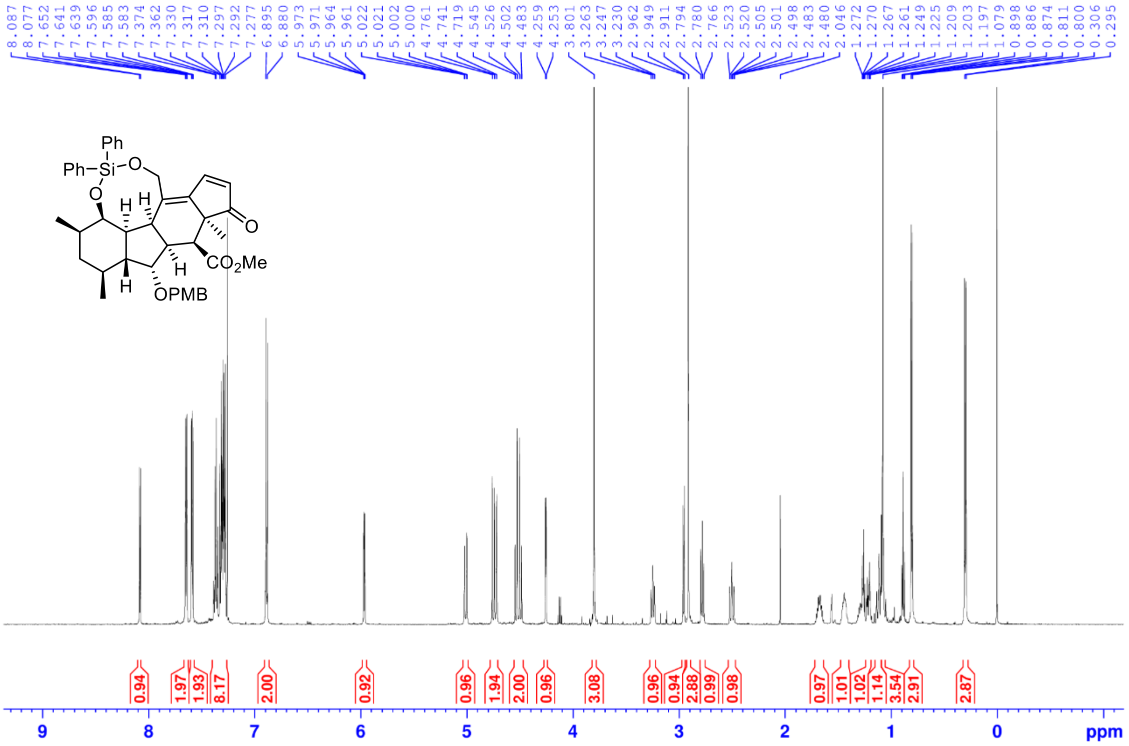

^{13}C NMR spectrum of compound **27** (150 MHz, $CDCl_3$)

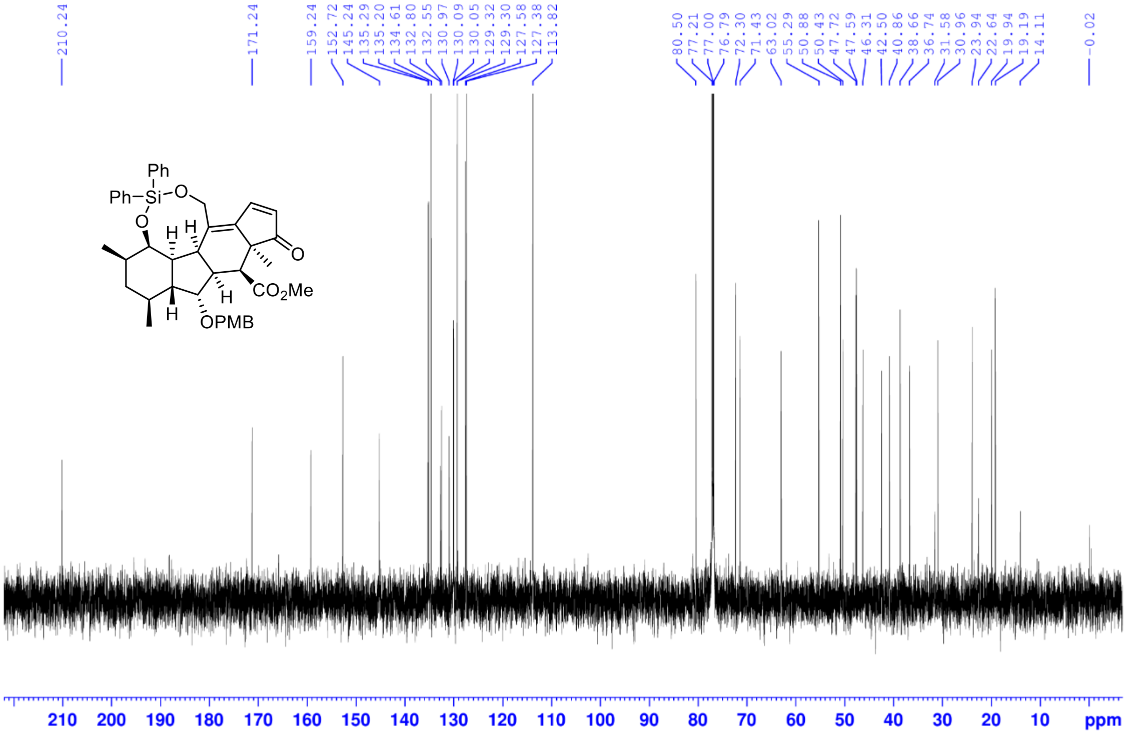

^{1}H NMR spectrum of compound **28** (600 MHz, $CDCl_3$)

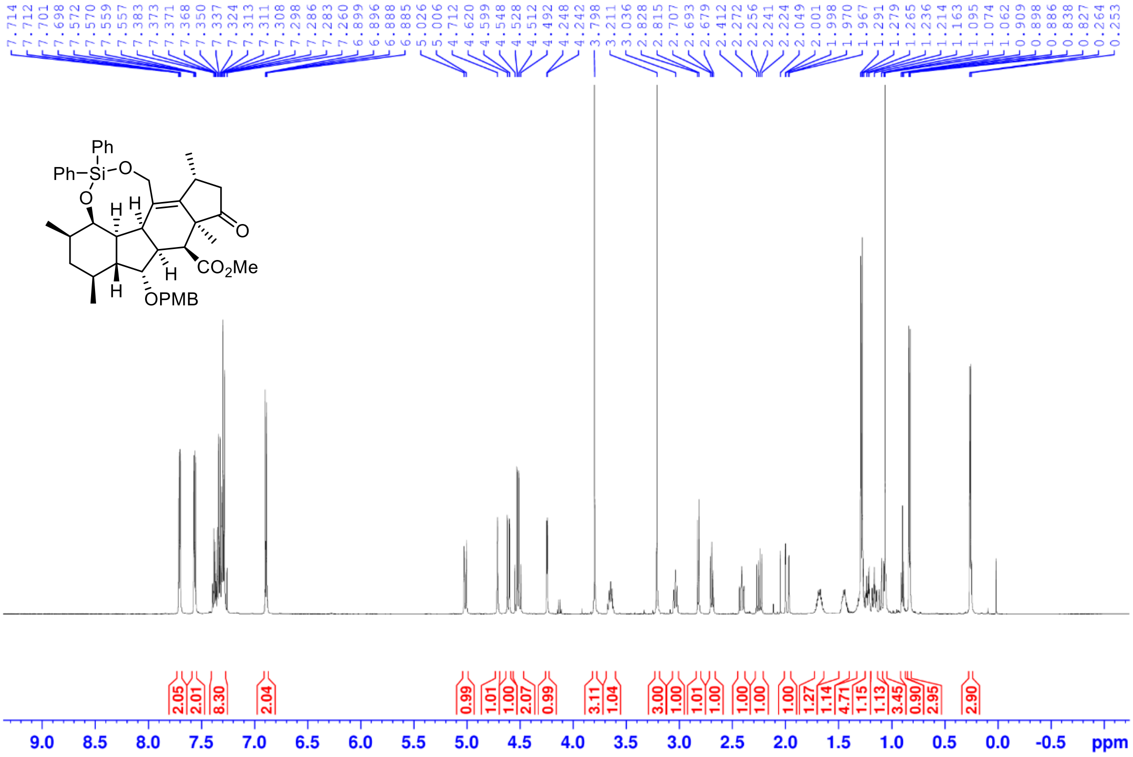

^{13}C NMR spectrum of compound **28** (150 MHz, $CDCl_3$)

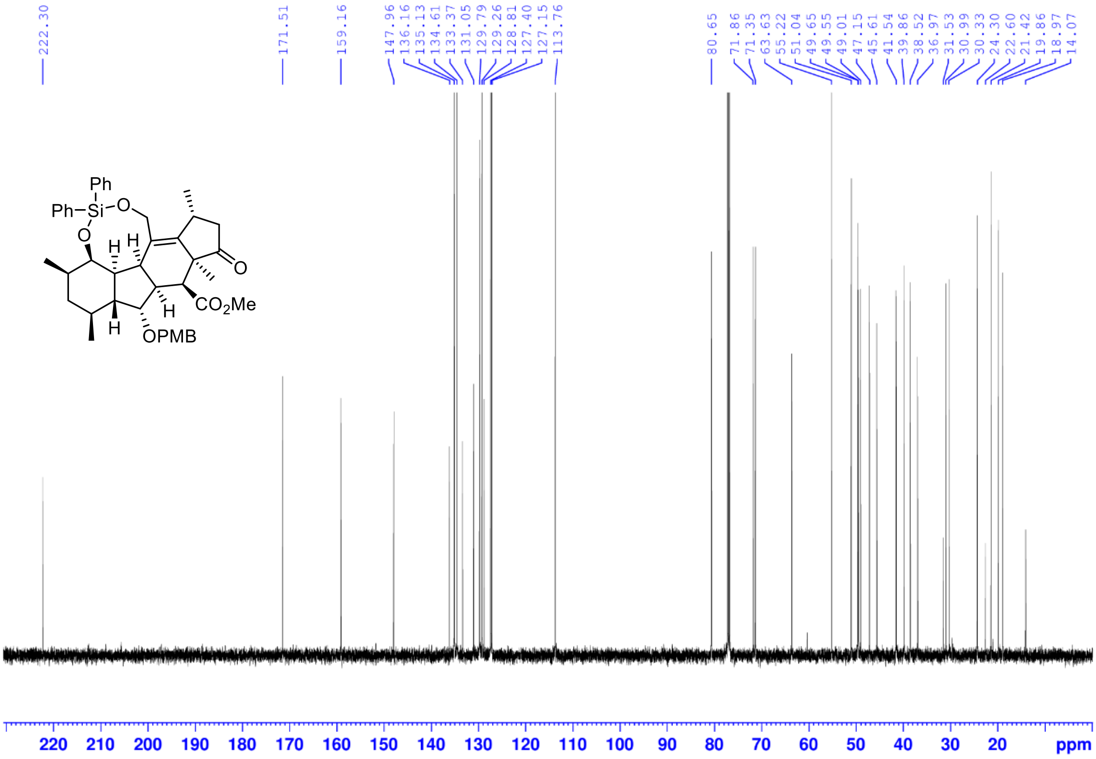

^{1}H NMR spectrum of compound **29** (600 MHz, $CDCl_3$)

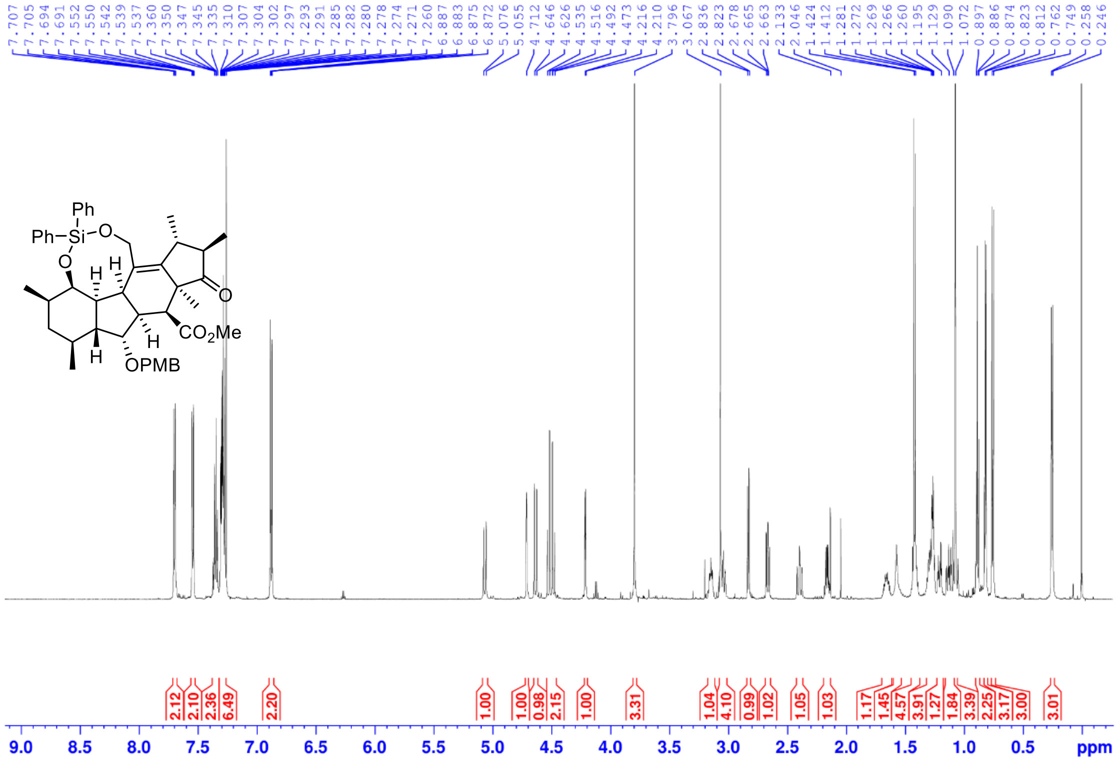

^{13}C NMR spectrum of compound **29** (150 MHz, $CDCl_3$)

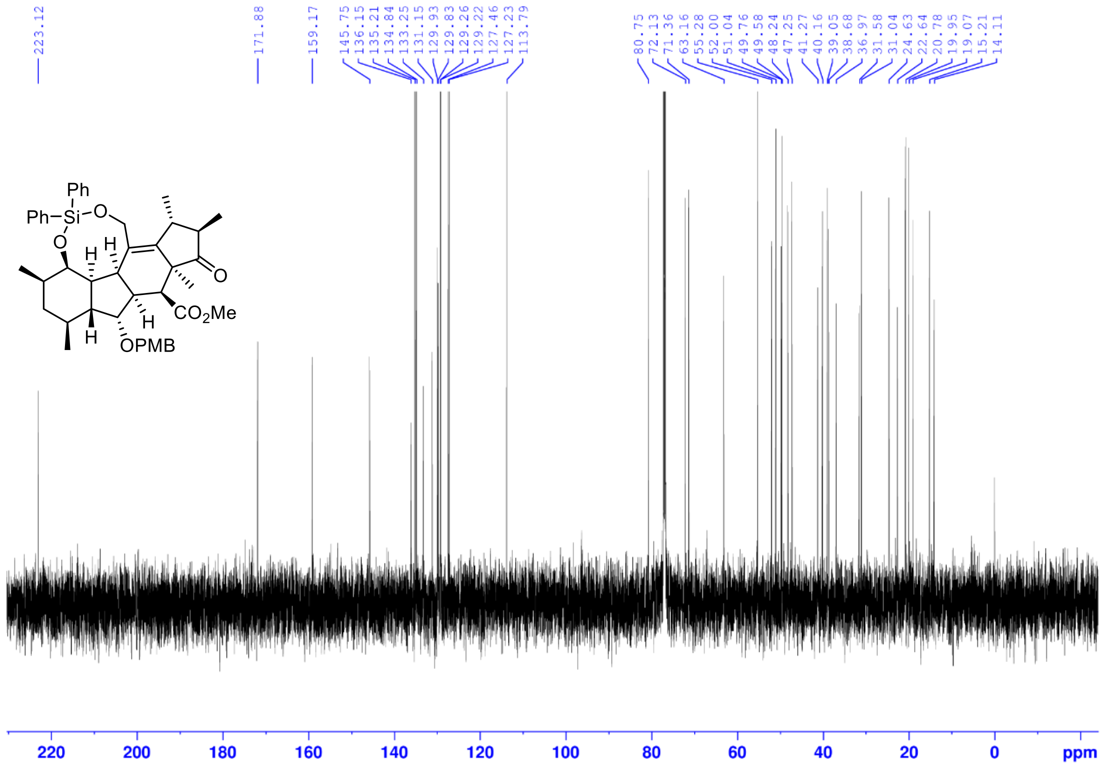

^{1}H NMR spectrum of compound **30** (600 MHz, $CDCl_3$)

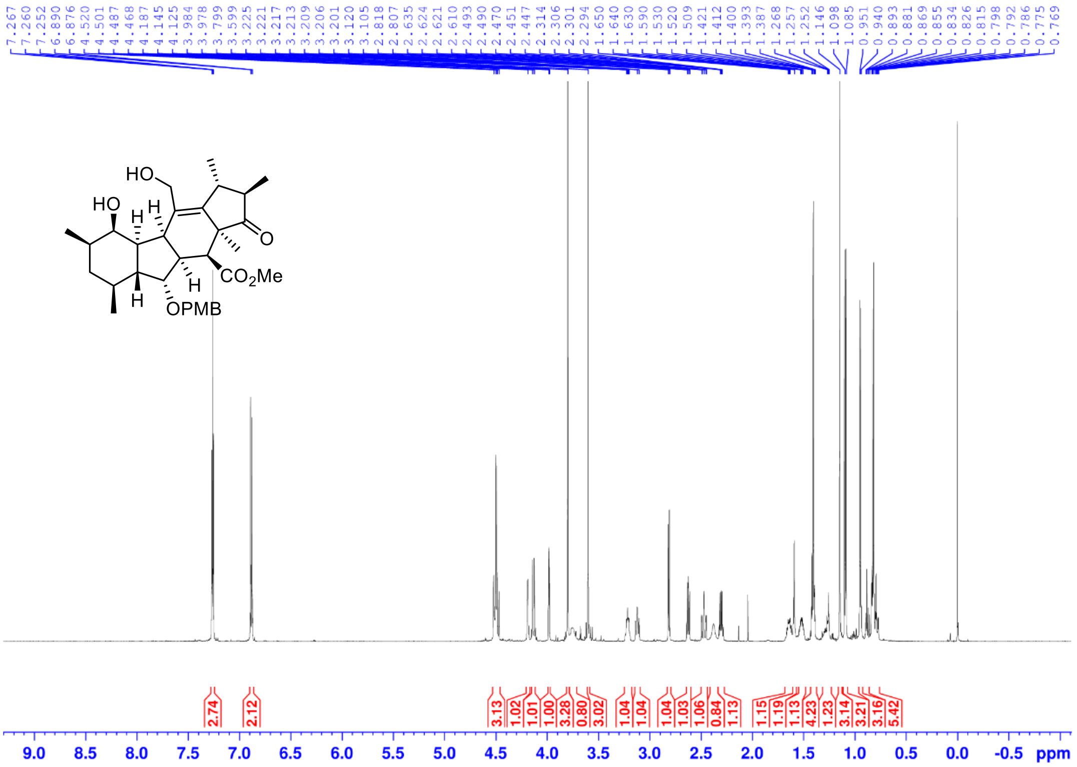

^{13}C NMR spectrum of compound **30** (150 MHz, $CDCl_3$)

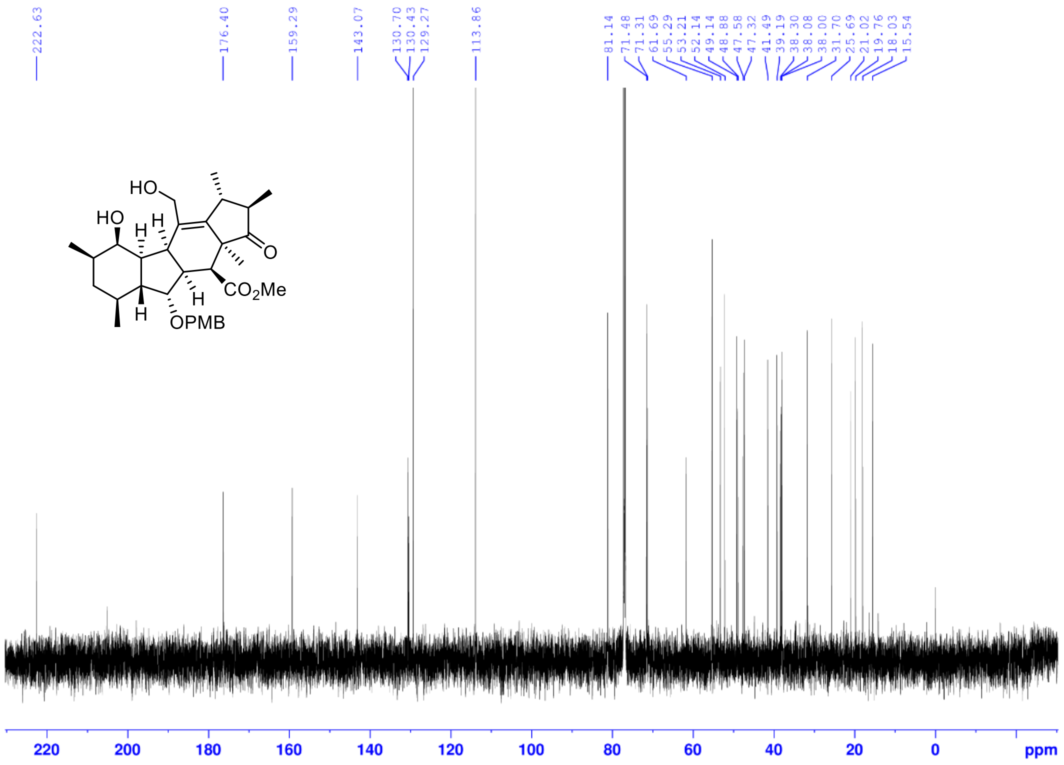

^{1}H NMR spectrum of compound **31** (600 MHz, $CDCl_3$)

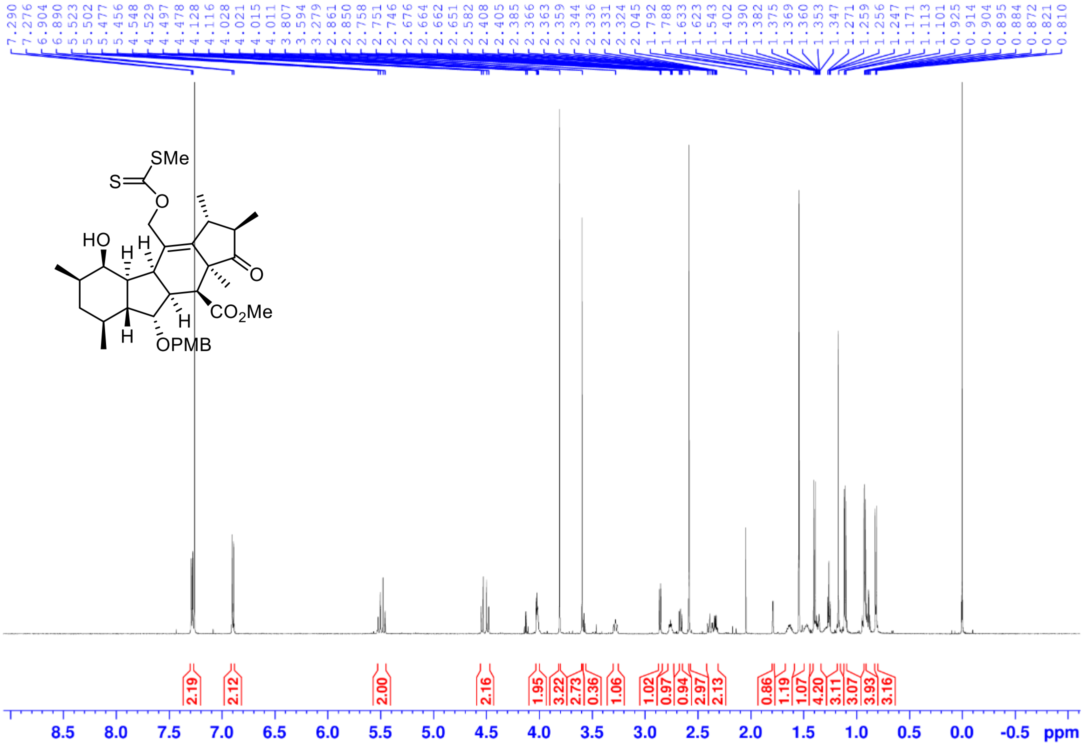

^{13}C NMR spectrum of compound **31** (150 MHz, $CDCl_3$)

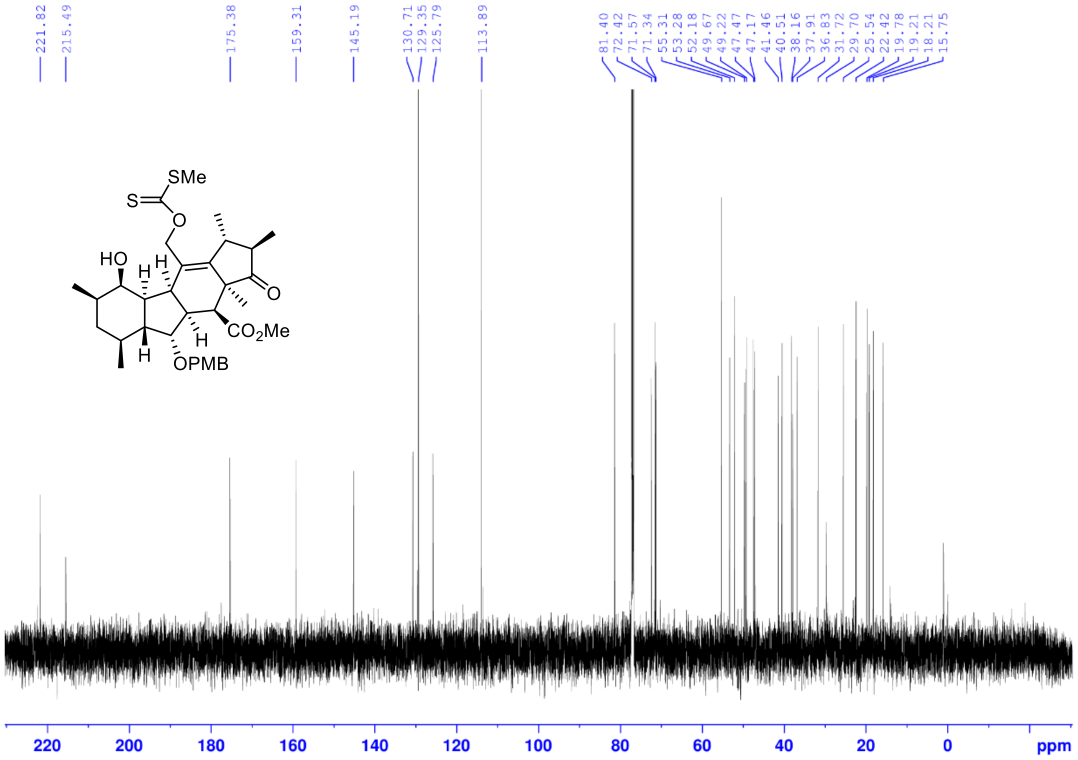

^{1}H NMR spectrum of compound **32** (600 MHz, $CDCl_3$)

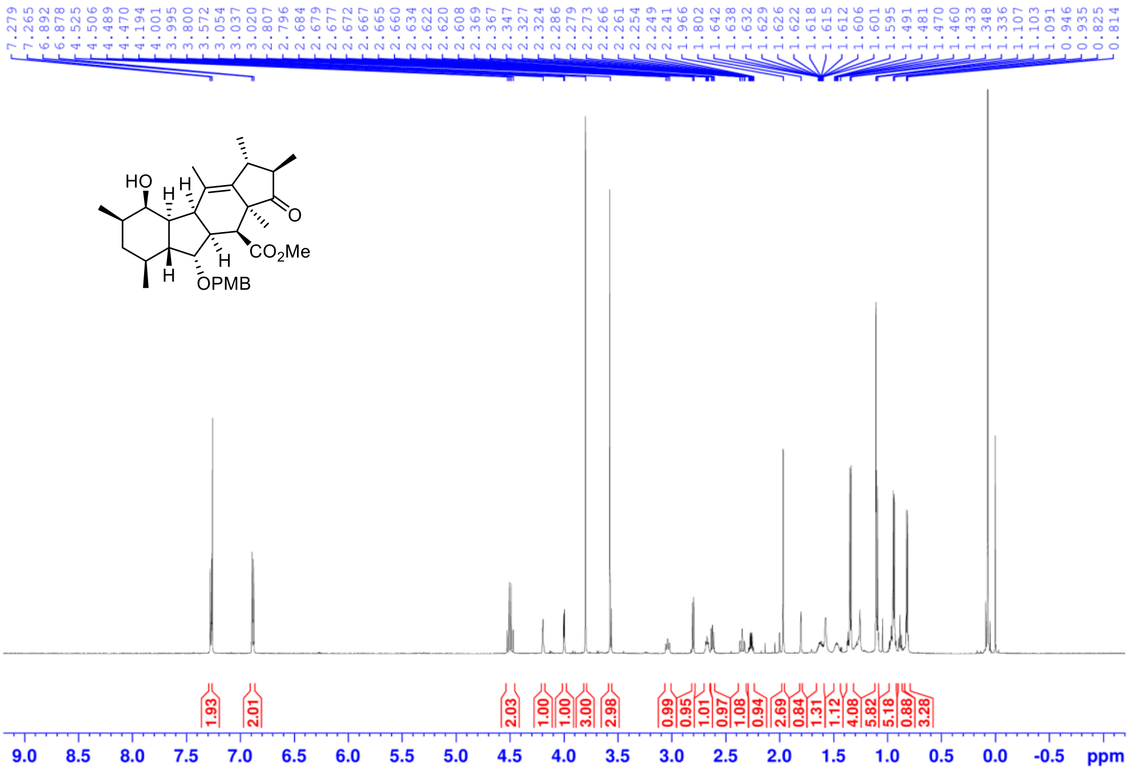

^{13}C NMR spectrum of compound **32** (150 MHz, $CDCl_3$)

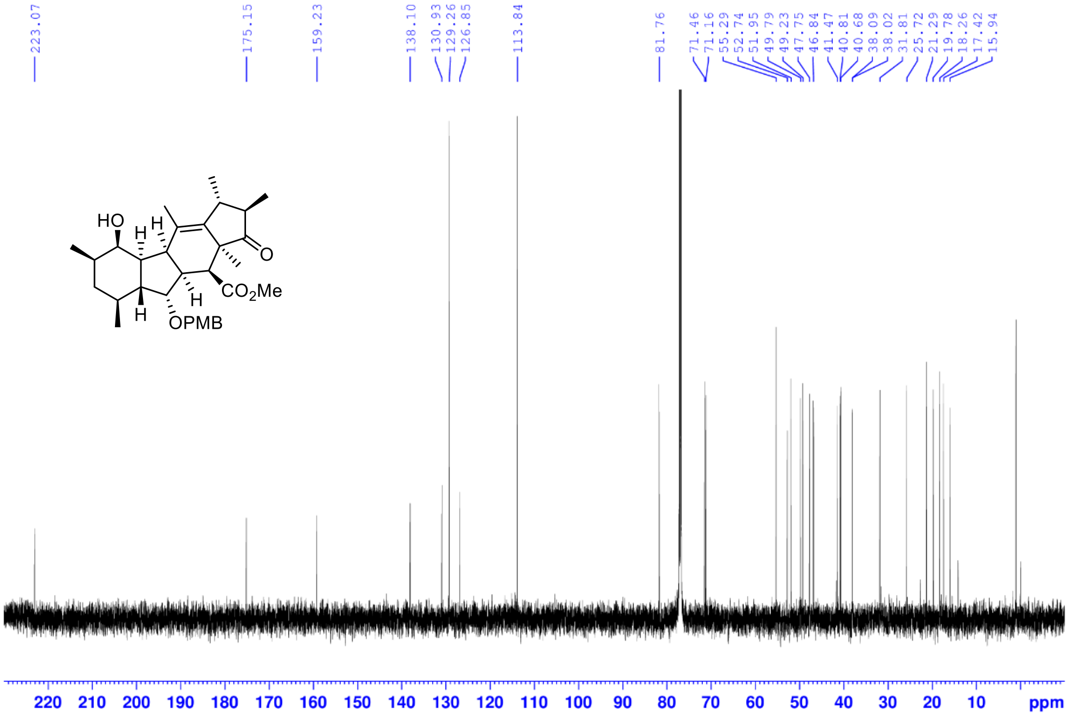

^{1}H NMR spectrum of compound **33** (600 MHz, $CDCl_3$)

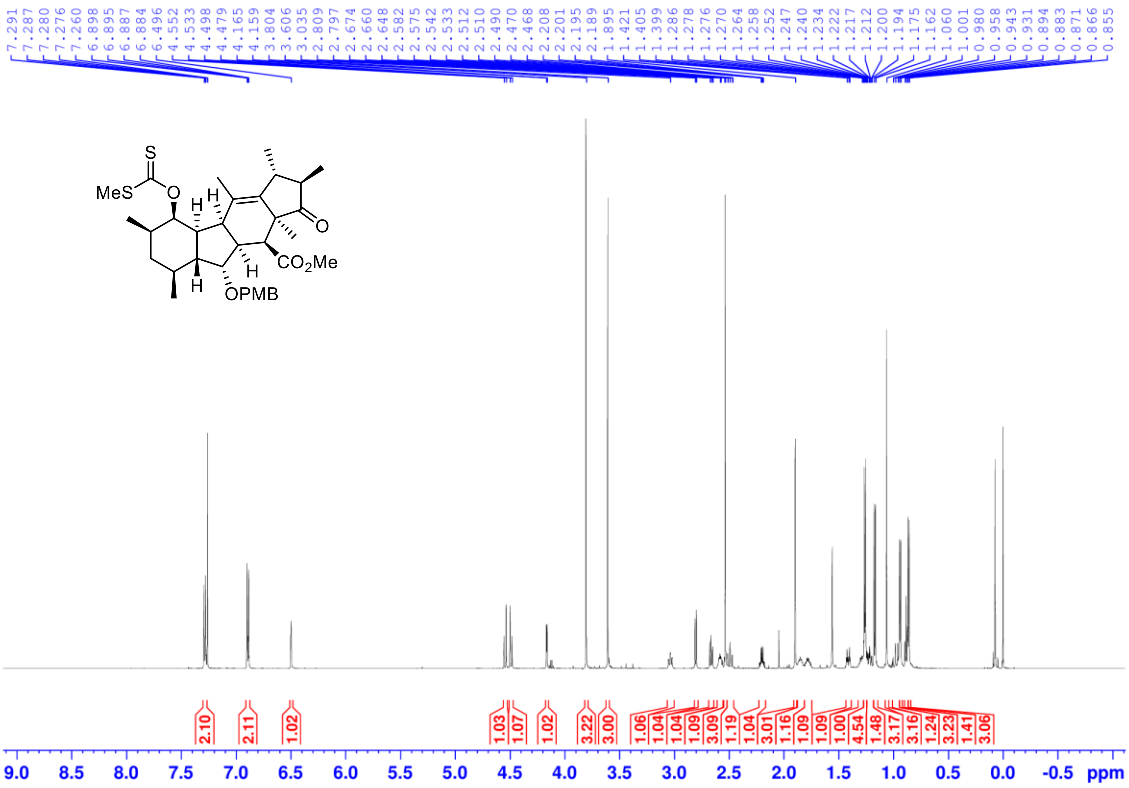

^{13}C NMR spectrum of compound **33** (150 MHz, $CDCl_3$)

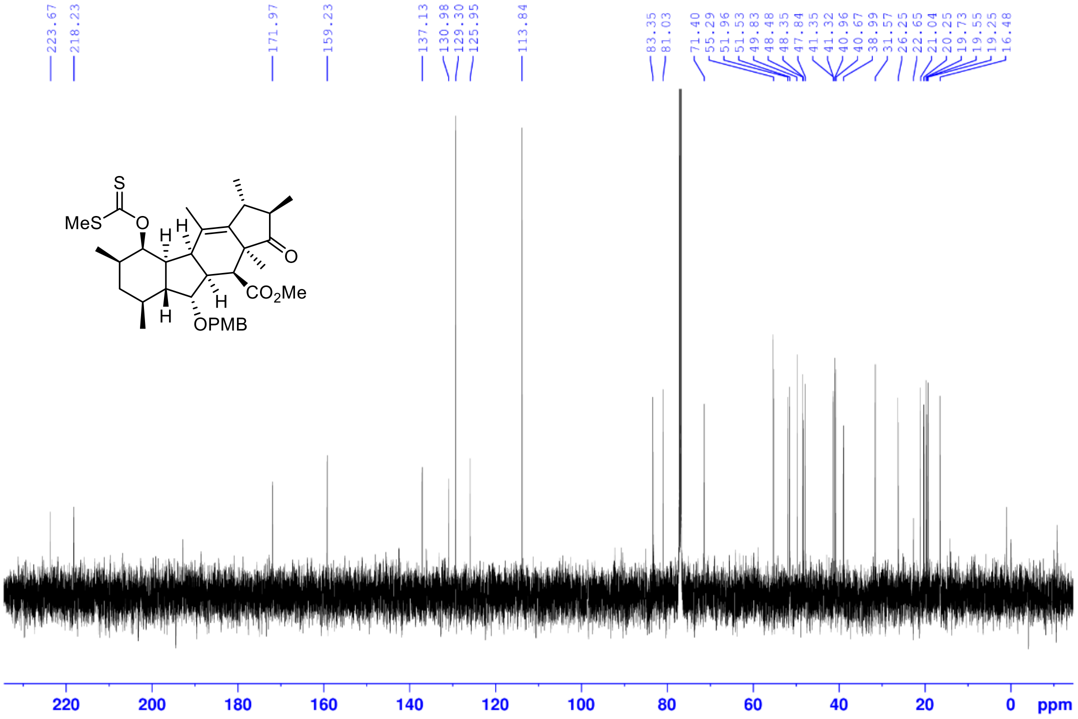

^{1}H NMR spectrum of compound **34** (600 MHz, $CDCl_3$)

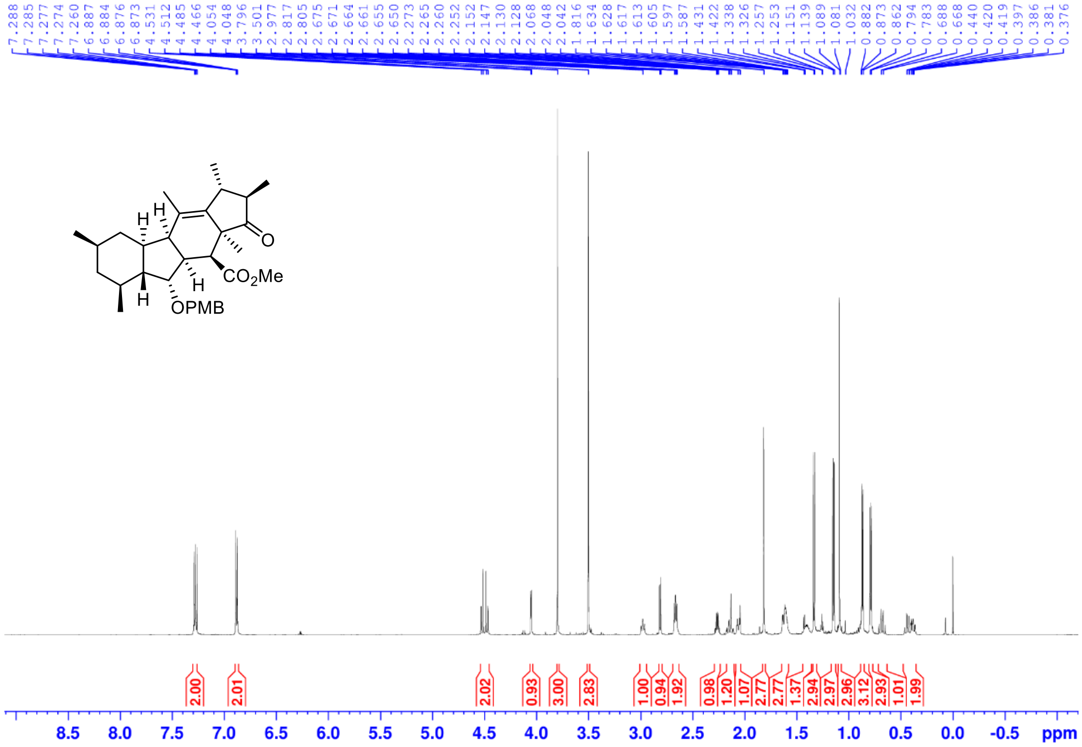

^{13}C NMR spectrum of compound **34** (150 MHz, $CDCl_3$)

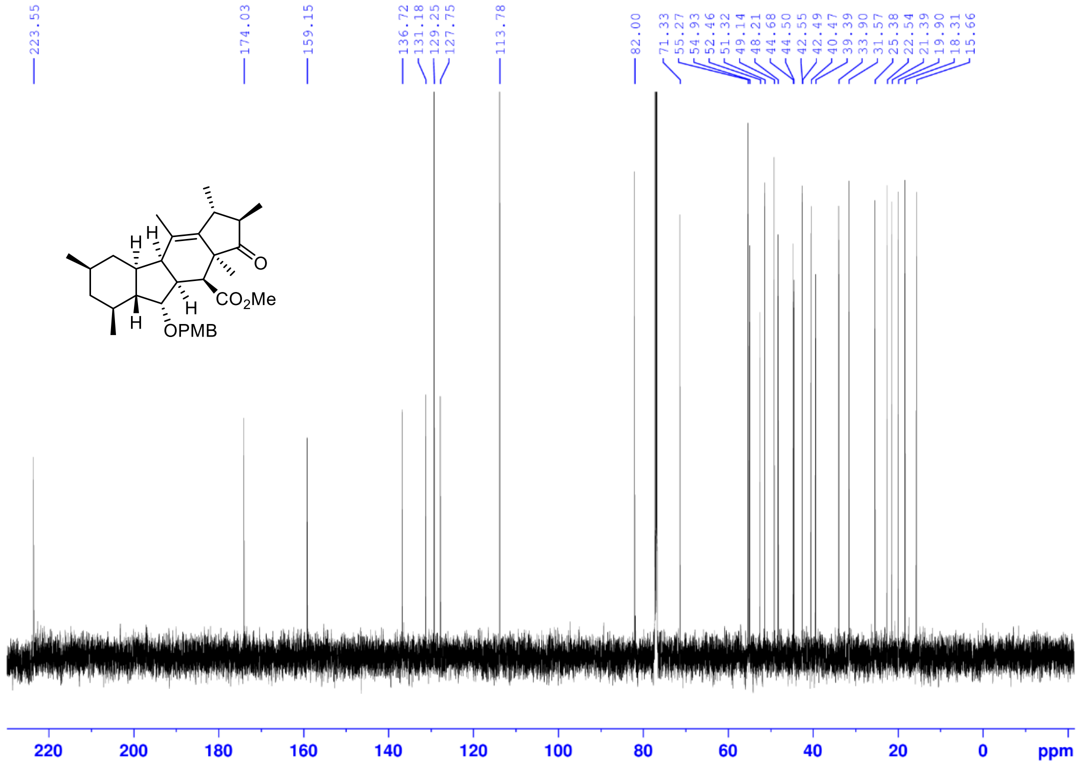

^{1}H NMR spectrum of compound **35** (600 MHz, $CDCl_3$)

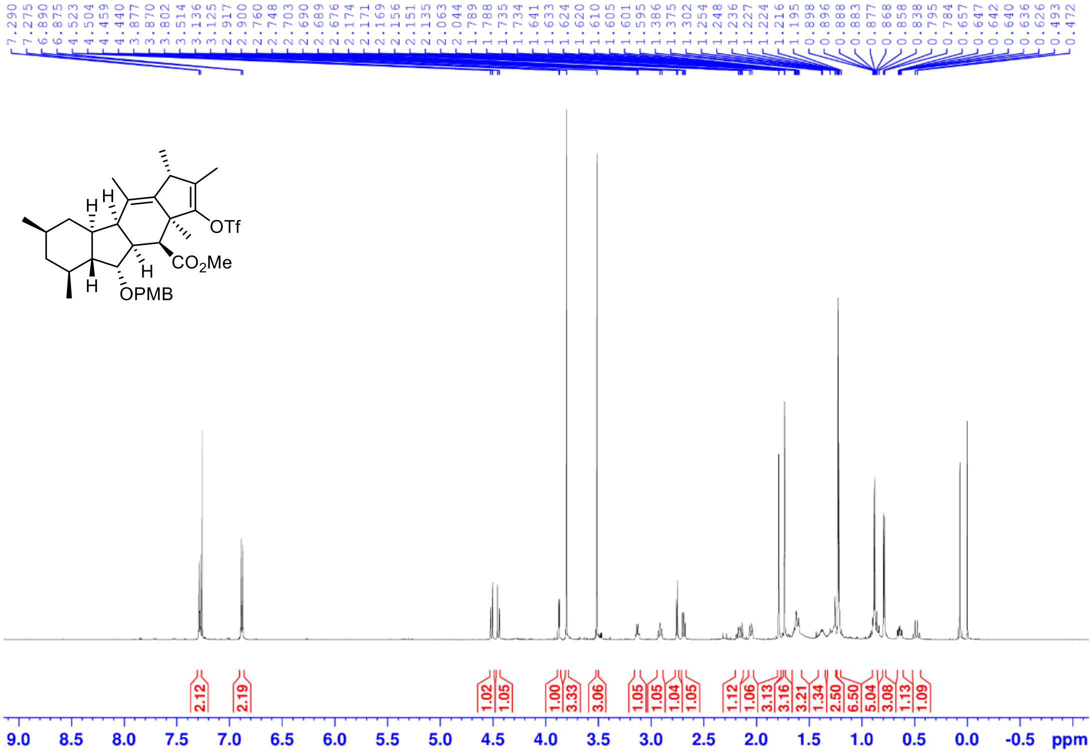

^{13}C NMR spectrum of compound **35** (150 MHz, $CDCl_3$)

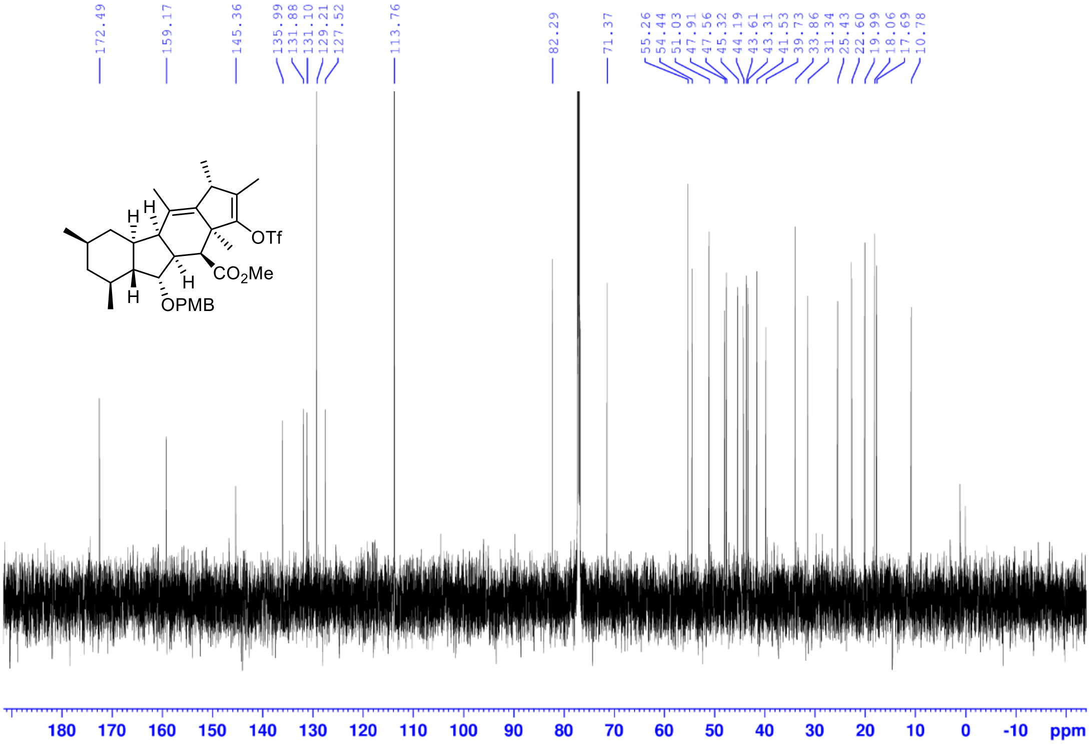

^{1}H NMR spectrum of compound **5** (600 MHz, $CDCl_3$)

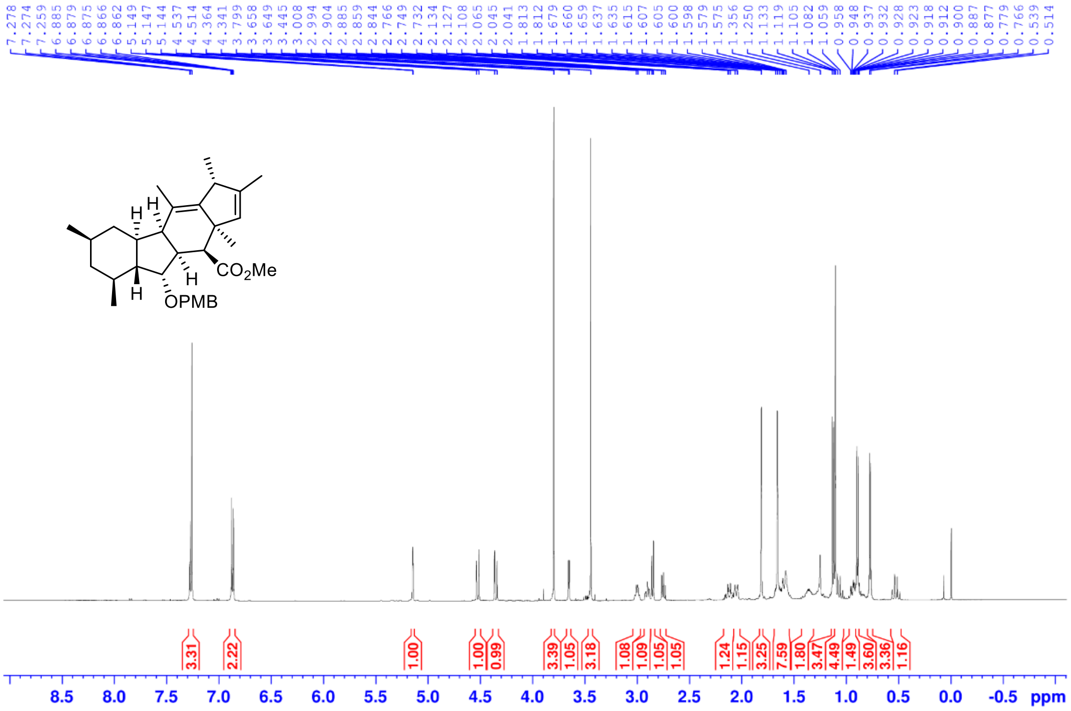

^{13}C NMR spectrum of compound **5** (150 MHz, $CDCl_3$)

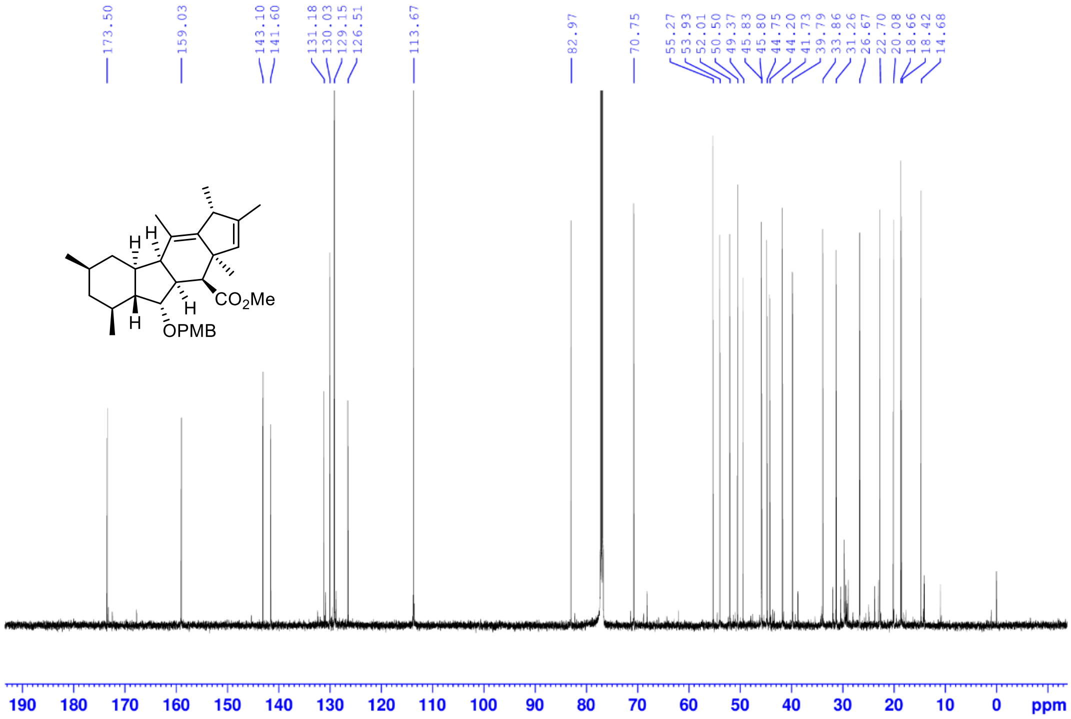

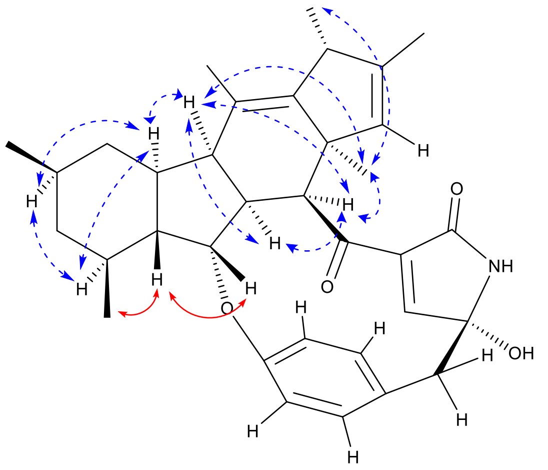

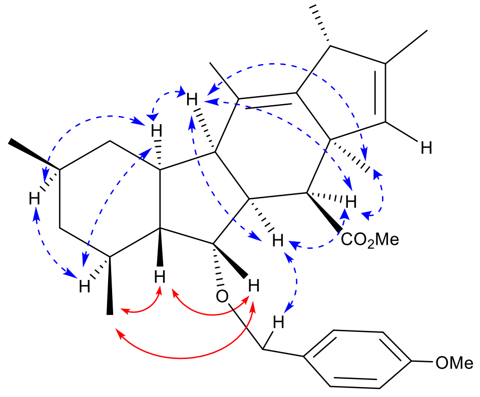

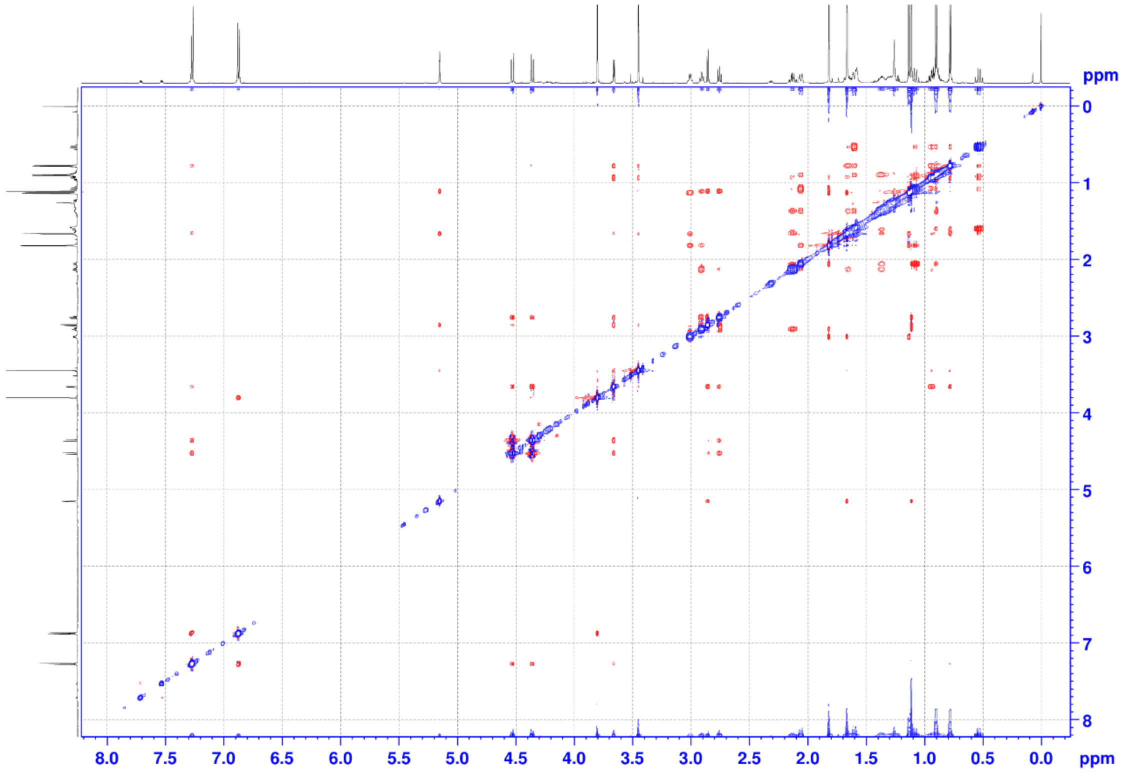

Figure S1. NOESY NMR spectrum of **5** (600 MHz, $CDCl_3$)

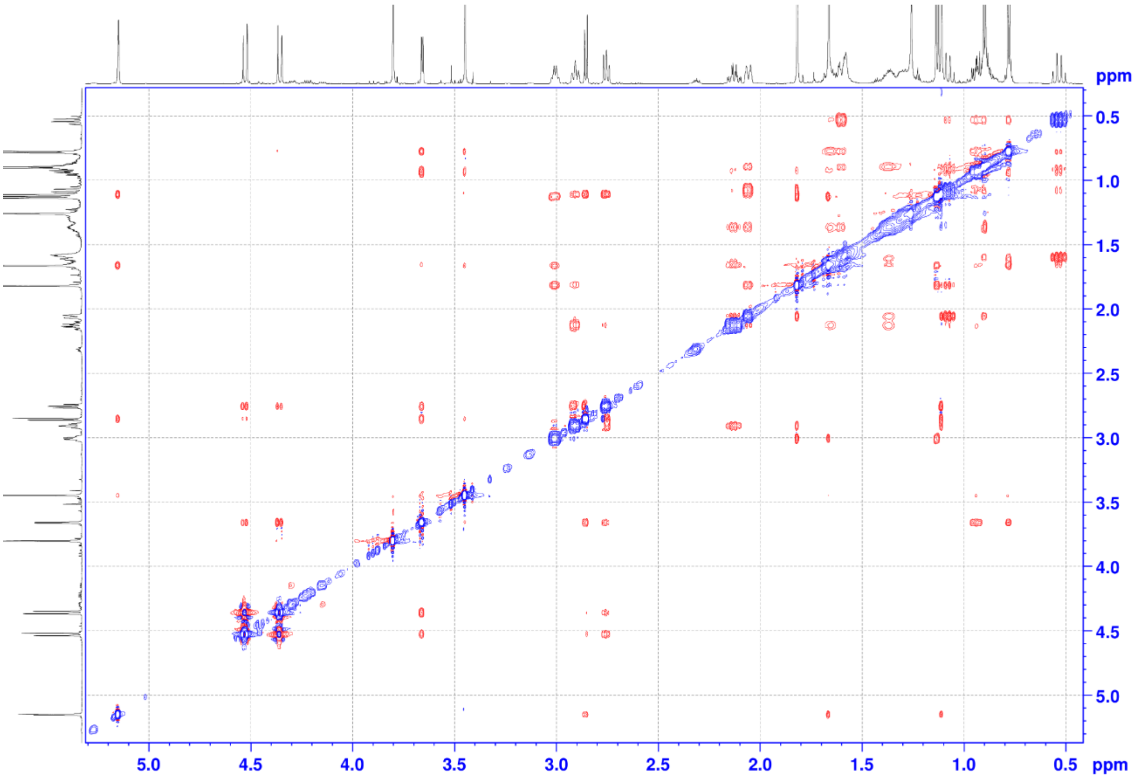

Figure S2. NOESY NMR spectrum of **5** (expansion) [600 MHz, $CDCl_3$]

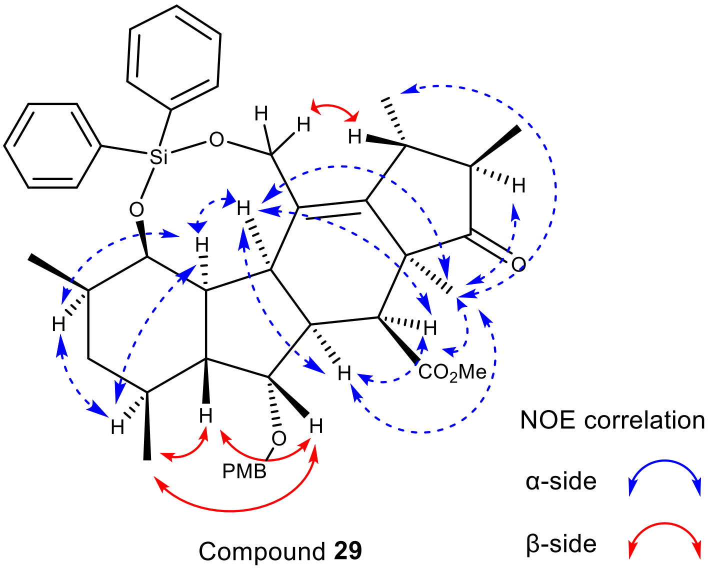

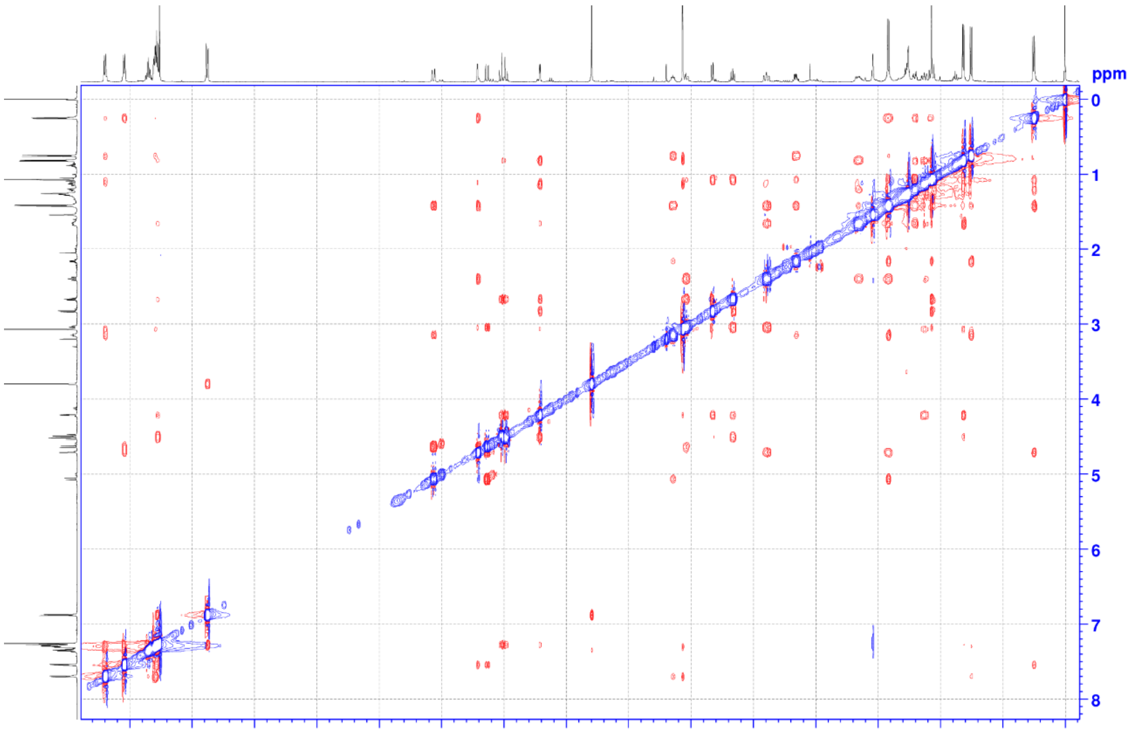

Figure S3. NOESY NMR spectrum of **29** (600 MHz, $CDCl_3$)

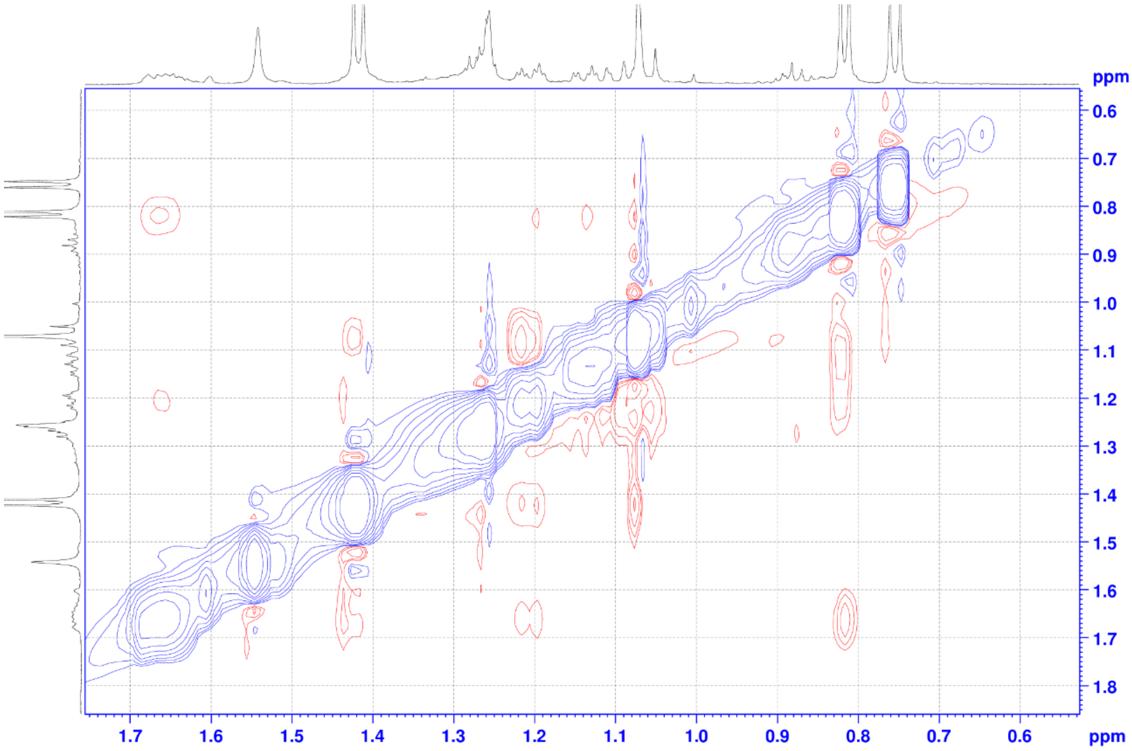

Figure S4. NOESY NMR spectrum of **29** (expansion) 〔600 MHz, $CDCl_3$〕
